# A population-based study on incidence trends of kidney and renal pelvis cancers in the United States over 2000–2020

**DOI:** 10.1038/s41598-024-61748-2

**Published:** 2024-05-17

**Authors:** Seyed Ehsan Mousavi, Morvarid Najafi, Armin Aslani, Asra Fazlollahi, Zahra Yekta, Mohammad Sadri, Seyed Aria Nejadghaderi

**Affiliations:** 1 Neurosciences Research Center, Aging Research Institute, Tabriz University of Medical Sciences, Tabriz, Iran; 2 Department of Community Medicine, Faculty of Medicine, Social Determinants of Health Research Center, Tabriz University of Medical Sciences, Tabriz, Iran; 3 School of Medicine, Tehran University of Medical Sciences, Tehran, Iran; 4 Student Research Committee, Tabriz University of Medical Sciences, Tabriz, Iran; 5 Calaveras County Department of Health, Calaveras County, CA USA; 6 Assistant Professor of Urology, Nephrology and Kidney Transplant Research Center, Clinical Research Institute, Urmia University of Medical Sciences, Urmia, Iran; 7 HIV/STI Surveillance Research Center, and WHO Collaborating Center for HIV Surveillance, Institute for Futures Studies in Health, Kerman University of Medical Sciences, Kerman, Iran; 8 Systematic Review and Meta‑analysis Expert Group (SRMEG), Universal Scientific Education and Research Network (USERN), Tehran, Iran

**Keywords:** Kidney neoplasm, Renal pelvis, Epidemiology, United States, Surveillance, epidemiology, and end results, SEER, Incidence, Cancer epidemiology, Renal cancer

## Abstract

Cancers of the kidney and renal pelvis are among the most prevalent types of urinary cancers. We aimed to outline the incidence trends of kidney and renal pelvis cancers by age, sex, race/ethnicity, and histology in the United States (US) from 2000 to 2020. The data was obtained from the Surveillance, Epidemiology, and End Results (SEER) 22 database. The identification of patients with kidney and renal pelvis cancers with morphologies of renal cell carcinoma, nephroblastoma, sarcoma, and neuroendocrine tumor was conducted utilizing the International Classification of Diseases for Oncology version 3. The average annual percent change (AAPC) were presented. All estimates were given in the form of counts and delayed age-standardized incidence rates (ASIRs) per 100,000 people. From 2000 to 2019, a total of 490,481 cases of kidney and renal pelvic cancer were recorded across all age groups in the US. The majority of them were among Non-Hispanic Whites (NHWs) (69.75%) and those aged 55–69 years (39.96%). The ASIRs per 100,000 for kidney and pelvis cancers were 22.03 for men and 11.14 for women. Non-Hispanic Black men had the highest ASIR (24.53 [24.24, 24.81]), and increase in ASIR over the 2000–2019 period (AAPC: 2.19% [1.84, 2.84]). There was a noticeable increase in incidence of kidney and renal pelvis cancers. Individuals aged 70–84 years had the highest ASIR for kidney and renal pelvis cancers. The COVID-19 era has resulted in a significant reduction in incidence rates across all demographics.

## Introduction

Kidney cancer develops from the cells composing the renal parenchyma, primarily those that line the renal tubules^1^. There are various morphological types of kidney cancer, with renal cell carcinoma (RCC) being the most common one^2^. Cancers of the renal pelvis develop mostly in the transitional epithelium, and are classified as either urothelial or non-urothelial carcinomas^3^.

In 2020, kidney cancer globally ranked 14th and 15th in terms of prevalence and death among all types of cancers, respectively^4^. By sex, kidney cancer ranked as the 9th most prevalent cancer in men and the 14th most prevalent one in females^4^. In 2020, a total of 431,288 new cases and 179,368 deaths attributed to kidney cancer were reported worldwide, with substantial variation in geographical distribution^4^. The incidence of kidney cancer steadily rises with age, and the global median age at which it is diagnosed is around 75 years^5^. Men have a roughly two-fold higher incidence rate of developing the cancer than women^1,6^. Furthermore, there have been association between smoking, hypertension, obesity, chronic kidney disease, occupational exposures, and radiation and the occurrence of kidney cancer^5^, as well as renal pelvis cancer^3^. Although the epidemiology of kidney cancer is extensively documented, little is known about the global epidemiology of renal pelvis cancer, primarily due to the fact that it is frequently reported in conjunction with other cancers of the urinary tract, making it difficult to study independently^3^. However, it is known that the incidence rates of renal pelvis cancer are significantly lower than those of kidney cancer^7^.

In the United States (US), older age groups are at higher risk of getting kidney and renal pelvis cancers, with men showing higher rate of developing them across all age groups^8^. Also, the incidence and mortality rates of these cancers are significantly higher than those of any other racial and ethnic groups^8^. Furthermore, the five-year relative survival rate of the kidney and renal pelvis cancers has increased significantly over the past four decades, rising from 50% over 1975–1977 to 77% for diagnoses over 2012–2018^8^.

A study utilizing data from the Surveillance, Epidemiology, and End Results (SEER) database calculated the incidence and mortality rates of RCC in the US between 1992 and 2015, but did not report other types of kidney and renal pelvis cancers and the estimates that it comprised have since become obsolete^9^. Furthermore, another study identified predictors of mortality and overall survival outcomes among patients with renal medullary carcinoma over 1996–2018^10^. Also, a recent study reported the trends in the incidence and mortality of genitourinary cancers in the US over the period of 1990 to 2020, whereas it did not stratified them by different histological subgroups^11^. Thus, a more exhaustive and precise examination of the most recent incidence trends of kidney and renal pelvis cancers is required. In addition, no prior research has examined how exactly the coronavirus disease 2019 (COVID-19) pandemic affected the incidence rates of kidney and renal pelvis cancers. Therefore, using the SEER data, we sought to provide comprehensive information regarding the incidence trends of kidney and renal pelvis cancers in the US from 2000 to 2020, stratified by age, sex, race/ethnicity, and histological subgroup. The COVID-19 effects on the overall incidence trends of these cancers were also evaluated.

## Methods

### Data source

The SEER Program, developed by the National Cancer Institute, functions as a comprehensive archieve of cancer data within the US. SEER 22 covers about half of the population and provides survival rates, cancer stage, patients' demographics, site and morphology of cancers, and treatments^12^. We estimated the incidence rates and annual percent changes (APCs) of kidney and renal pelvis cancers from the years 2000 to 2020^13,14^. The SEER 22 database was accessed in compliance with the SEER Research Data Agreement for the 1975–2020 Data (November 2022 Submission)^15^, and cancer statistics were published in accordance with the SEER 22 guideline^16^.

### Definitions

The valeus were presented as frequencies and percentages, with the incidence rate expressed as the number of cases per 100,000 individuals. The APCs of kidney and renal pelvis cancers over a specific timeframe exhibit variation at a constant proportion of the preceding year’s rate. The average annual percent changes (AAPCs) denote the arithmetic average of several APCs over a given time period. The patients were classifiedinto three distinct groups based on their ethnicity: Non-Hispanic White (NHW), Non-Hispanic Black (NHB), and Hispanic. Neverthless, other races/ethnicities like American Indian/Alaska Native, Native Hawaiian, and Asian/Pacific Islander were not reported seperately as the numbers of cases were limited. The identification of patients with kidney and renal pelvis cancers was conducted utilizing the International Classification of Diseases for Oncology version 3, as RCC (codes 8260, 8310–8313, 8316, 8317, 8319, 8323, 8480, and 8510), nephroblastoma (code 8960), sarcoma (codes 8800, 8900, 8901, 8910, 8912, 8920, 8964, 9040, 9120, 9180, and 9364), and neuroendocrine tumor (codes 8013, 8041, and 8240).

### Statistical analysis

The present study employed the SEER 22 Research Limited-Field Data with Delay-Adjustment database spanning from 2000 to 2020^13^, which was acquired from SEER*Stat, version 8.4.1.2^17^, with the purpose of determining the delay age-standardized incidence rate (ASIR) of kidney and renal pelvis cancers^18^. These modified counts, along with the corresponding delay model, can be employed to enhance the accuracy in identifying prevailing cancer patterns^18^. The inclusion criteria for this study limited the sample to individuals who had been diagnosed with malignant cancer and for whom the age at diagnosis was available. Subsequently, the delay model was utilized, integrating adjustment factors for various variables like demographic, cancer site, and the year of diagnosis^19,20^. In addition, the ASIRs of renal pelvis and kidney cancer subtypes were determined using the SEER 22 Research Limited-Field Data database for the period of 2000–2020^14^. The ASIRs were estimated using the Tiwari technique, with the 2000 US standard population as the reference, and the corresponding 95% confidence intervals (CIs)^21^, using the SEER*Stat version 8.4.1.2^17^.

The Joinpoint Regression Program, version 5.0.2^22^ was utilized for estimating the APCs and AAPCs^23^, joinpoint regression modelling, parallelism test, and coincident test^24^ for ASIR^25^. The utilization of the parallelism test involved conducting a pairwise comparison in order to assess whether the trends exhibited by the two groups demonstrated similarity throughout the course of time^24^. Additionally, a pairwise comparison was conducted using the coincidence test to ascertain whether the rates of the two groups remained identical throughout the duration of the study. The year 2020 marked the onset of the COVID-19 pandemic. Consequently, the inclusion of the 2020 incidence data has the potential to introduce bias into the estimates of cancer incidence. Therefore, it was deliberately omitted from the Joinpoint trends analysis and is solely presented in illustrations. The APC of kidney and renal pelvis cancers ASIRs were calculated by fitting the least-squares regression lines to the natural logarithm of the ASIR, with the year of diagnosis as the independent variable. The minimum number of observations required between two joinpoints, as well as the minimum number of observations required from each joinpoint to either end of the data, were both specified as two. The weighted Bayesian Information Criteria approach was employed for model selection^26^. The empirical quantile method was used to compute the 95% CIs of AAPCs^27^.

## Results

The examination of SEER data uncovered clear patterns across three defined categories: “Kidney and renal pelvis cancers” section, “Kidney cancer” section, “Histopathological variants in kidney cancers” section, including “RCC” section, “Nephroblastoma” section, “Sarcoma” section, and “Neuroendocrine tumor” section, and “Renal pelvis” cancer section. Each category displayed distinct features and trends, as discussed in the following sections:

### Kidney and renal pelvis cancers

#### Overall incidence

**Figure 1:**
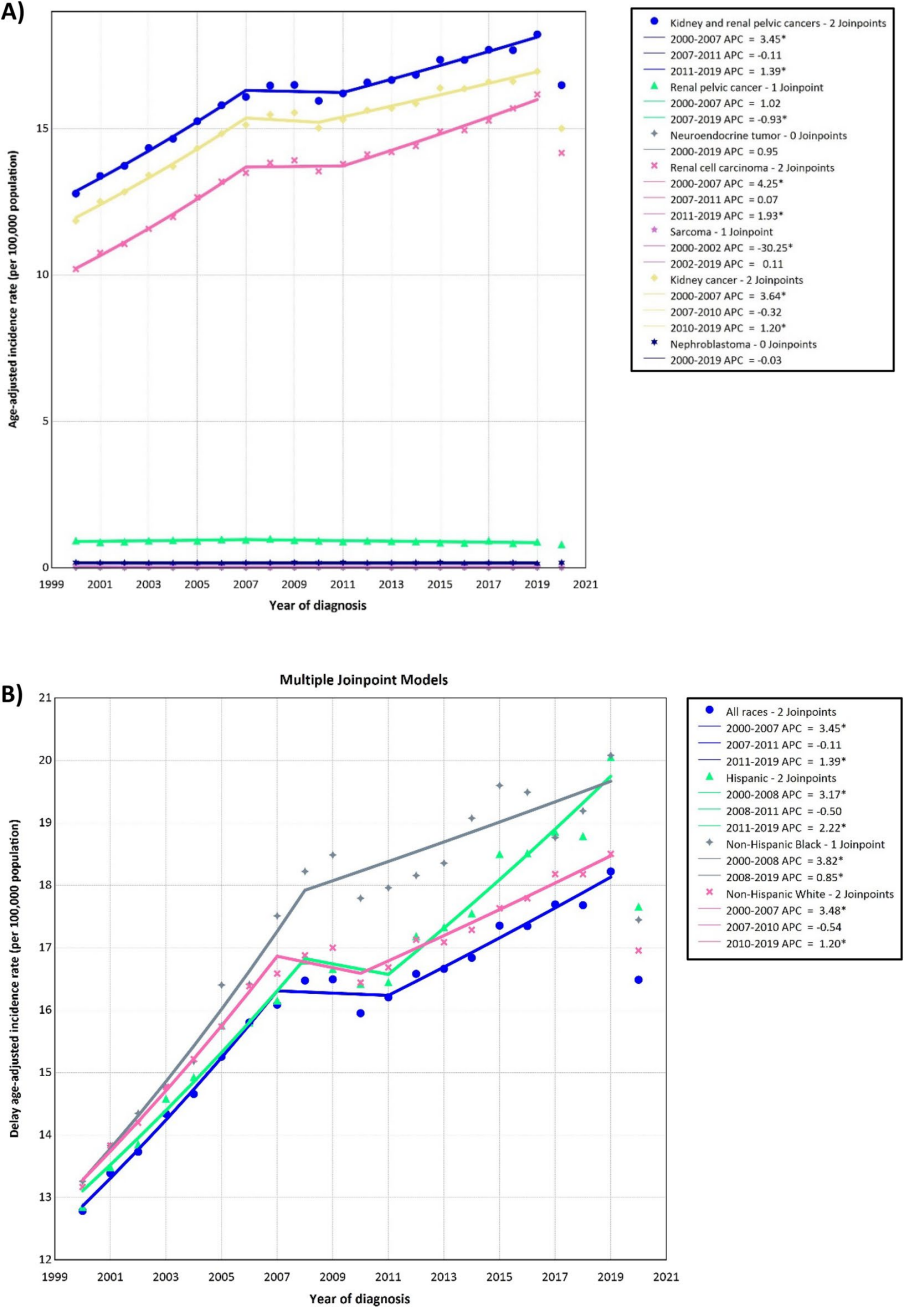

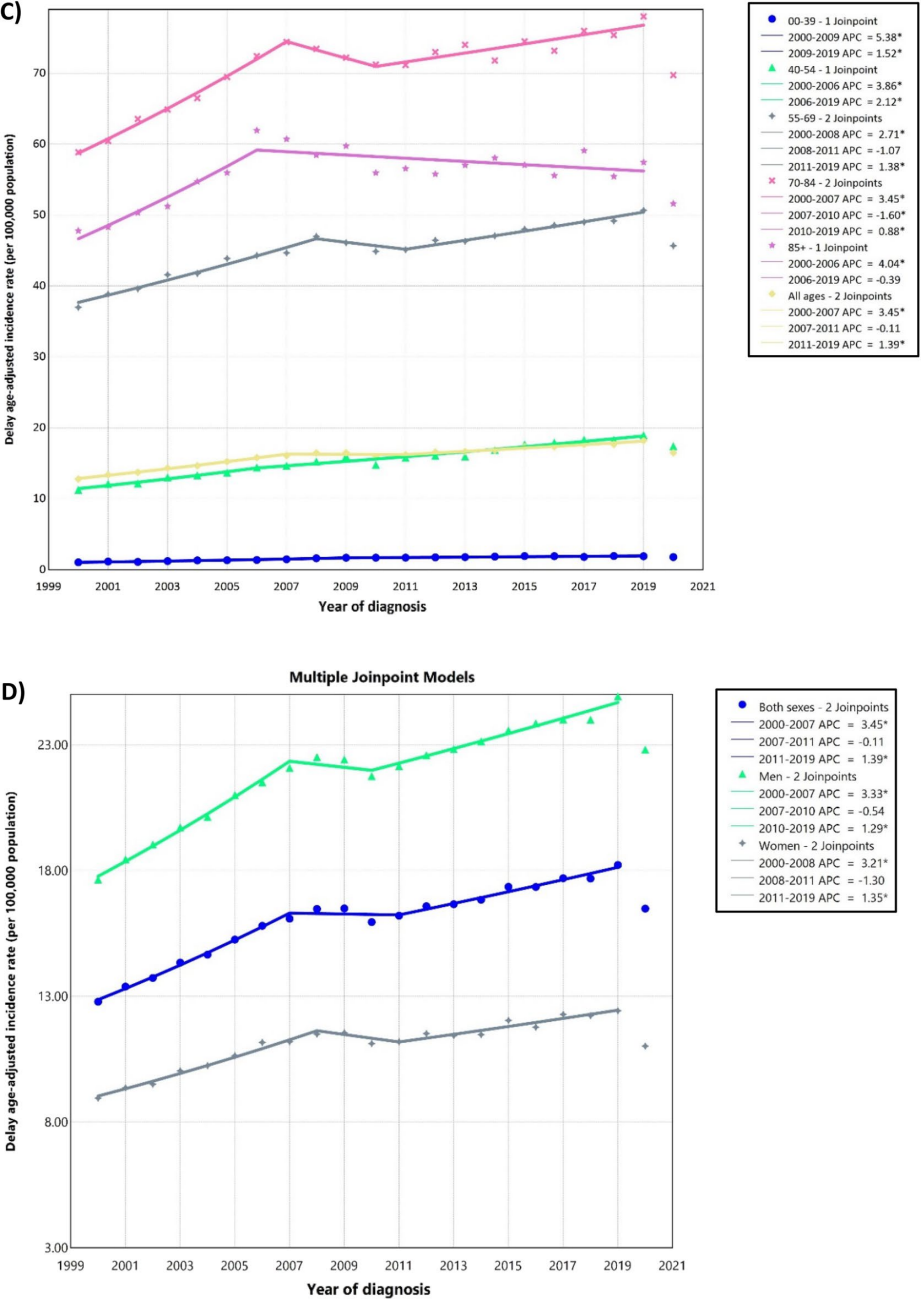
Delayed age-adjusted incidence rate of kidney and renal pelvis cancers over 2000–2019 and in 2020 in the United States, by cancer subtypes (**A**), race (**B**), age (**C**), and sex (**D**). APC: annual percent change. * Represent *p*-value less than 0.05.

Between 2000 and 2019, there were a total of 490,481 cases of kidney and renal pelvis cancers across all age groups. The majority of these cases were found among NHWs (69.75%) and individuals aged 55–69 years (39.96%). The ASIRs per 100,000 population were 22.03 (21.95, 22.11) for men and 11.14 (11.09, 11.19) for women. NHB men had the highest ASIR (24.53 [24.24, 24.81]). Both sexes experienced an increase in ASIRs over 2000–2019 (AAPC: 1.74% [1.66, 1.87] for men and AAPC: 1.70% [1.56, 1.90] for women) with NHB men showed the most significant increase in ASIR over the 2000–2019 period (AAPC: 2.19% [1.84, 2.84]) (Table 1, Fig. 1A–D).

**Table 1:** Counts and age-standardized rate of kidney and renal pelvis cancers incidence per 100,000 and average annual percent change from 2000 to 2019 in the United States by age, sex, and race.

Age group (years)	Men			Women		
	Case (%)	Delayed ASIR (95% CI)	AAPC (95% CI)	Case (%)	Delayed ASIR (95% CI)	AAPC (95% CI)
All race/ethnicities						
All	307,397 (62.67)	22.03 (21.95, 22.11)	1.74 (1.66, 1.87)	183,084(37.33)	11.14 (11.09, 11.19)	1.7 (1.56, 1.9)
0–39	13,841 (2.82)	1.77 (1.74, 1.8)	3.34 (3.01, 3.73)	10,889 (2.22)	1.4 (1.37, 1.42)	3.19 (2.62, 3.9)
40–54	62,686 (12.78)	20.27 (20.12, 20.43)	2.69 (2.29, 3.11)	33,483 (6.83)	10.56 (10.45, 10.68)	2.68 (1.91, 3.51)
55–69	128,634 (26.23)	62.9 (62.56, 63.25)	1.54 (1.42, 1.73)	67,347 (13.73)	29.93 (29.71, 30.16)	1.47 (1.34, 1.68)
70–84	88,723 (18.09)	100.1(99.44, 100.76)	1.38 (1.26, 1.54)	57,641 (11.75)	49.15 (48.75, 49.55)	1.32 (1, 1.62)
+ 85	13,513 (2.76)	85.05 (83.62, 86.5)	0.78 (0.48, 1.23)	13,724 (2.80)	42.08 (41.38, 42.79)	0.75 (0.37, 1.21)
Hispanic						
All	43,250 (59.80)	22.33 (22.1, 22.56)	2.04 (1.85, 2.41)	29,070 (40.20)	12.62 (12.47, 12.77)	2 (1.65, 2.46)
0–39	3444 (4.76)	1.59 (1.54, 1.65)	4.24 (3.62, 5)	3015 (4.17)	1.44 (1.39, 1.5)	4.45 (3.33, 5.87)
40–54	11,855(16.39)	20.06 (19.7, 20.42)	2.55 (2.25, 2.9)	7358 (10.17)	12.46 (12.17, 12.74)	3.19 (2.62, 3.89)
55–69	17,168 (23.74)	63.41 (62.46, 64.38)	2.2 (1.68, 2.86)	10,693 (14.79)	34.95 (34.29, 35.62)	2.08 (1.63, 2.65)
70–84	9564 (13.22)	104.24(102.15,106.37)	1.62 (1.37, 2.04)	6859 (9.48)	54.14 (52.86, 55.44)	0.89 (0.25, 1.65)
+ 85	1219 (1.69)	89.18 (84.24, 94.33)	0.08 (− 1, 1.52)	1145 (1.58)	45.07 (42.5, 47.76)	0.87 (− 0.02, 2.04)
NHB						
All	32,229 (60.35)	24.53 (24.24, 24.81)	2.19 (1.84, 2.84)	21,174 (39.65)	12.43 (12.26, 12.6)	2.03 (1.63, 2.58)
0–39	1907 (3.57)	2.06 (1.97, 2.15)	2.46 (1.34, 3.7)	1537 (2.88)	1.52 (1.45, 1.6)	1.54 (0.48, 2.7)
40–54	8053 (15.08)	23.79 (23.27, 24.31)	2.56 (1.9, 3.3)	4341 (8.13)	11.34 (11, 11.68)	1.74 (0.97, 2.58)
55–69	14,691 (27.51)	75.96 (74.73, 77.2)	1.91 (1.46, 2.48)	8550 (16.01)	35.38 (34.63, 36.14)	2.68 (2.39, 3.12)
70–84	6874 (12.87)	102.05(99.63, 104.52)	1.83 (1.41, 2.39)	5650 (10.58)	53.48 (52.1, 54.9)	2.03 (1.12, 3.34)
+ 85	704 (1.32)	74.23 (68.85, 79.92)	− 0.37 (− 2.24, 1.91)	1096 (2.05)	45.63 (42.97, 48.42)	0.35 (− 1.33, 2.36)
NHW						
All	217,513 (63.58)	22.6 (22.5, 22.7)	1.71 (1.62, 1.85)	124,618(36.42)	11.2 (11.13, 11.26)	1.64 (1.48, 1.81)
0–39	7548 (2.21)	1.89 (1.85, 1.93)	3.31 (2.66, 4.03)	5677 (1.66)	1.46 (1.42, 1.5)	3.04 (2.35, 3.78)
40–54	39,427 (11.52)	20.74 (20.54, 20.95)	2.69 (2.1, 3.25)	20,073 (5.87)	10.53 (10.39, 10.68)	2.69 (2.23, 3.25)
55–69	90,806 (26.54)	63.79 (63.38, 64.21)	1.39 (1.16, 1.7)	45,063 (13.17)	29.69 (29.42, 29.97)	1.19 (1.05, 1.35)
70–84	68,658 (20.07)	103.2(102.42, 103.97)	1.4 (1.27, 1.58)	42,802 (12.51)	49.83 (49.36, 50.31)	1.17 (0.91, 1.49)
+ 85	11,074 (3.24)	88.42 (86.78, 90.08)	0.89 (0.62, 1.28)	11,003 (3.22)	42.48 (41.69, 43.28)	0.86 (0.28, 1.61)

*NHW* Non-Hispanic White, *NHB* Non-Hispanic Black, *ASIR* Age-standardized incidence rate, *CI* Confidence interval, *AAPC* Average annual percent change.

In the more recent period of 2015–2019, a total of 153,758 cases of kidney and renal pelvis cancers were reported in the US. Similarly, the majority of cases were among NHWs (65.47%) and individuals aged 55 to 69 years (42.34%). The ASIRs per 100,000 population during this period were 24.08 (23.93, 24.24) for men and 12.15 (12.05, 12.26) for women. Among all races and ethnicities, NHB men had the highest reported ASIRs (26.98 [26.44, 27.52]). There was a significant increase in ASIRs over the 2015–2019 period in both sexes, with an AAPC of 1.29% (1.09, 1.80) in men and an AAPC of 1.35% (0.93, 2.34) in women (Table S1).

The results of identical and parallel trends for kidney and renal pelvis cancers are shown in Table S2 and Table S3, respectively.

##### Men

From 2000 to 2019, a total of 307,397 (62.67%) cases of kidney and renal pelvis cancers were diagnosed in men. Most cases were diagnosed at the age of 50–69 years (41.85%) and among NHWs (70.76%). Cases between 70 and 84 years had the highest ASIR among all age groups (100.10 [99.44, 100.76]). All age groups showed a significant increase from 2000 to 2019, with individuals below 39 years experiencing the greatest increase among all other age groups (3.34% [3.01, 3.73]) (Table 1).

Of all the reported cases, 7.11% were among Hispanic men. The majority of cases fell within the age range of 55–69 years (39.69%). The overall delayed ASIR per 100,000 population was 22.33 (22.10, 22.56), with cases between 70 and 84 years having the highest delayed ASIR (104.24 [102.15, 106.37]). The overall AAPC for Hispanic men was 2.04% (1.85, 2.41) (Table 1).

Of all men, 32,229 (10.48%) cases were NHBs. Most cases (45.58%) were between 55 and 69 years old. The delayed ASIR per 100,000 population was 24.53 (24.24, 24.81), with those between 70 and 84 years having the highest ASIR (102.05 [99.63, 104.52). NHBs had a substantial increase in ASIR with an AAPC of 2.19% (1.84, 2.84) (Table 1).

The majority of NHW men were between 55 and 69 years (41.75%). The overall delayed ASIR per 100,000 population was 22.60 (22.50, 22.70), with cases between 70 and 84 years having the highest delayed ASIR over the period from 2000 to 2019 (103.20 [102.42, 103.97]). The overall AAPC for NHWs was 1.71% (1.62, 1.85), indicating a significant increase in incidence rate from 2000 to 2019 (Table 1).

##### Women

A total of 183,084 cases of kidney and renal pelvis cancers were reported over 2000–2019 among women in the US. The majority of cases occurred in NHWs (68.10%) and between 55 and 69 years (36.78%). Cases between 70 and 84 years had the highest delayed ASIR among all age groups (49.15 [48.75, 49.55]). All ages had a steady increase in ASIR from 2000 to 2019 with individuals under the age of 39 years having the greatest increase in ASIR (AAPC: 3.19% [2.62, 3.90]) (Table 1).

Of all the women 15.88% were Hispanics and the majority of cases were between 50 and 69 years (39.78%). The delayed ASIR per 100,000 population in Hispanic women was 12.62 (12.47, 12.77) with individuals between 70 and 84 years having the highest ASIR (54.14 [52.86, 55.44]). Over 2000–2019, there was a significant increase in the ASIR of Hispanic women (AAPC: 2.00% [1.65, 2.46]) (Table 1).

Among women, 11.57% of reported cases were NHBs and the majority of cases (40.38%) were between 50 and 69 years old. The delayed ASIR per 100,000 population in NHB women was 12.43 (12.26, 12.60). Cases between 70 and 84 years had the highest ASIR (53.48 [52.10, 54.90]). The overall AAPC was 2.03% (1.63, 2.58) (Table 1).

The majority of NHW cases were between 50 and 69 years (36.16%) with the delayed ASIR per 100,000 population in NHW women was 11.20 (11.13, 11.26). Those between 70 and 84 years had the highest ASIR (49.83 [49.36, 50.31]). Over 2000–2019, there was a significant increase in ASIR of NHW women (AAPC: 1.64% [1.48, 1.81]) (Table 1).

#### Age and sex patterns

Between 2000 and 2019, across all racial and ethnic groups, men showed higher incident cases in most age groups with the highest incident cases in the 65–69 age group for both men and women. The delay-adjusted incidence rate of kidney and renal pelvis cancers showed a slight decrease from ages 0–4 to 20–24. However, starting at age 20–24, both sexes underwent a significant increase, peaking at the age of 75–79 years for both sexes (Figure S1).

#### COVID-19 impacts

There was a significant decrease in the ASIR of kidney and renal pelvis cancers across all races/ethnicities in both sexes within all age groups (AAPC: − 9.52 [− 10.98, − 8.06]) and for males (AAPC: − 8.49 [− 10.34, − 6.65]) and females (AAPC: − 11.31 [− 13.72, − 8.90]) from 2019 to November 2020 (Table 2).

**Table 2:** Percent change in delay-adjusted age-standardized incidence rates of kidney and renal pelvis cancers from 2019 to 2020 by race and sex, using the November 2022 data submission.

Races/ethnicities	Sex	Age	2019 Delayed ASIR (95% CI)	2020 Delayed ASIR (95% CI)	PC (95% CI)
All	Female	All ages	12.43 (12.2, 12.66)	11.02 (10.8, 11.24)	− 11.31 (− 13.72, − 8.9)
All	Male	All ages	24.93 (24.58, 25.28)	22.81 (22.48, 23.15)	− 8.49 (− 10.34, − 6.65)
All	Both	All ages	18.22 (18.02, 18.43)	16.49 (16.29, 16.68)	− 9.52 (− 10.98, − 8.06)
Hispanic	Female	All ages	15.1 (14.49, 15.73)	13 (12.44, 13.58)	− 13.91 (− 19.05, − 8.77)
Hispanic	Male	All ages	25.92 (25.03, 26.84)	23.13 (22.3, 23.98)	− 10.79 (− 15.25, − 6.32)
Hispanic	Both	All ages	20.05 (19.53, 20.59)	17.66 (17.17, 18.15)	− 11.95 (− 15.3, − 8.6)
NHB	Female	All ages	14.03 (13.31, 14.78)	12.24 (11.56, 12.95)	− 12.77 (− 19.45, − 6.08)
NHB	Male	All ages	27.95 (26.77, 29.18)	24.11 (23.02, 25.24)	− 13.75 (− 19.16, − 8.33)
NHB	Both	All ages	20.08 (19.43, 20.75)	17.45 (16.84, 18.07)	− 13.12 (− 17.31, − 8.94)
NHW	Female	All ages	12.19 (11.9, 12.49)	11.02 (10.73, 11.31)	− 9.64 (− 12.88, − 6.39)
NHW	Male	All ages	25.59 (25.15, 26.04)	23.64 (23.21, 24.08)	− 7.64 (− 9.97, − 5.3)
NHW	Both	All ages	18.5 (18.24, 18.77)	16.96 (16.7, 17.21)	− 8.37 (− 10.27, − 6.48)

*NHW* Non-Hispanic White, *NHB* Non-Hispanic Black, *ASIR* Age-standardized incidence rate, *CI* Confidence interval, *PC* percent change.

### Kidney cancer

#### Overall incidence

From 2000 to 2019, there were 463,401 kidney cancer cases in all age groups in the US. The most commonly reported subtype was RCC (90.31%). The majority of cases were men (62.95%), NHWs (69.06%), and between 55 and 69 years (40.59%). The ASIR per 100,000 population was 20.72 (20.64, 20.79) for men and 10.42 (10.37, 10.47) for women. NHB men had the highest ASIR (23.77 [23.49, 24.04]). The AAPCs for men and women were 1.78% (1.68, 1.94) and 1.70% (1.50, 1.97), respectively. Hispanic women had the greatest rise in ASIRs compared to other groups over 2000–2019 (2.20% [1.59, 3.06]) (Table 3, Figure S2, Figure S3, and Figure S4).

**Table 3:** Counts and age-standardized rate of all morphologies of kidney cancer incidence per 100,000 and average annual percent change from 2000 to 2019 in the United States, by age, sex, and race.

Age group (years)	Men			Women		
	Case (%)	ASIR (95% CI)	AAPC (95% CI)	Case (%)	ASIR (95% CI)	AAPC (95% CI)
All race/ethnicities						
All	291,701(62.95)	20.72 (20.64, 20.79)	1.78 (1.68, 1.94)	171,700 (37.05)	10.42 (10.37, 10.47)	1.7 (1.5, 1.97)
0–39	13,735 (2.96)	1.75 (1.72, 1.78)	3.34 (2.99, 3.74)	10,837 (2.34)	1.39 (1.36, 1.41)	3.15 (2.6, 3.81)
40–54	61,469 (13.26)	19.82 (19.66, 19.98)	2.7 (2.38, 3.11)	32,861 (7.09)	10.33 (10.22, 10.45)	2.69 (2, 3.4)
55–69	123,547(26.66)	60.12 (59.79, 60.46)	1.58 (1.43, 1.78)	64,531 (13.93)	28.55 (28.33, 28.77)	1.55 (1.36, 1.81)
70–84	81,111 (17.50)	90.98 (90.35, 91.61)	1.41 (1.31, 1.55)	51,661 (11.15)	43.9 (43.52, 44.28)	1.29 (1.03, 1.55)
+ 85	11,839 (2.55)	74.18 (72.85, 75.53)	0.49 (0, 1.36)	11,810 (2.55)	36.06 (35.41, 36.72)	0.5 (0.04, 1.1)
Hispanic						
All	41,916 (59.80)	21.3 (21.07, 21.52)	2.07 (1.72, 2.55)	28,182 (40.20)	12.07 (11.92, 12.21)	2.2 (1.59, 3.06)
0–39	3419 (4.88)	1.57 (1.52, 1.63)	4.24 (3.61, 5)	3002 (4.28)	1.43 (1.38, 1.48)	4.15 (2.9, 6.46)
40–54	11,693 (16.68)	19.67 (19.31, 20.03)	2.47 (2.17, 2.82)	7290 (10.40)	12.27 (11.99, 12.55)	3.12 (2.56, 3.81)
55–69	16,711 (23.84)	61.28 (60.34, 62.22)	2.21 (1.67, 2.86)	10,448 (14.90)	33.91 (33.26, 34.57)	2.04 (1.56, 2.67)
70–84	8978 (12.81)	97.07 (95.05, 99.11)	1.69 (1.37, 2.2)	6415 (9.15)	50.27 (49.04, 51.52)	0.84 (0.24, 1.56)
+ 85	1115 (1.59)	80.99 (76.31, 85.89)	− 0.09 (− 1.26, 1.48)	1027 (1.47)	40.15 (37.73, 42.68)	0.71 (− 0.17, 1.87)
NHB						
All	31,541 (60.56)	23.77 (23.49, 24.04)	2.00 (1.67, 2.48)	20,538 (39.44)	11.95 (11.79, 12.12)	1.98 (1.6, 2.49)
0–39	1897 (3.64)	2.03 (1.94, 2.13)	2.37 (1.23, 3.66)	1527 (2.93)	1.51 (1.43, 1.58)	1.45 (0.42, 2.58)
40–54	7947 (15.26)	23.34 (22.83, 23.86)	2.47 (1.78, 3.28)	4270 (8.20)	11.09 (10.76, 11.43)	1.69 (0.8, 2.66)
55–69	14,398 (27.65)	73.9 (72.69, 75.13)	1.9 (1.45, 2.52)	8349 (16.03)	34.3 (33.57, 35.04)	2.58 (2.13, 3.12)
70–84	6623 (12.72)	97.53 (95.17, 99.93)	1.79 (1.27, 2.56)	5361 (10.29)	50.39 (49.04, 51.76)	2 (0.83, 3.69)
+ 85	676 (1.30)	70.81 (65.57, 76.35)	− 0.56 (− 2.47, 1.77)	1031 (1.98)	42.67 (40.1, 45.36)	0.03 (− 1.84, 2.2)
NHW						
All	204,633(63.94)	21.14 (21.05, 21.23)	1.72 (1.59, 1.87)	115,391 (36.06)	10.4 (10.34, 10.46)	1.54 (1.34, 1.82)
0–39	7485 (2.34)	1.87 (1.83, 1.91)	3.28 (2.65, 3.98)	5653 (1.77)	1.45 (1.41, 1.49)	2.99 (2.31, 3.74)
40–54	38,536 (12.04)	20.22 (20.02, 20.43)	2.76 (2.35, 3.22)	19,642 (6.14)	10.28 (10.14, 10.43)	2.7 (2.3, 3.11)
55–69	86,741 (27.10)	60.68 (60.28, 61.09)	1.37 (1.04, 1.84)	42,858 (13.39)	28.13 (27.86, 28.4)	1.31 (1.15, 1.52)
70–84	62,255 (19.45)	93.13 (92.4, 93.86)	1.44 (1.31, 1.62)	37,870 (11.83)	44 (43.56, 44.45)	0.95 (0.67, 1.27)
+ 85	9616 (3.00)	76.48 (74.96, 78.03)	0.52 (− 0.06, 1.43)	9368 (2.93)	36.04 (35.32, 36.78)	0.59 (0, 1.4)

*NHW* Non-Hispanic White, *NHB* Non-Hispanic Black, *ASIR* Age-standardized incidence rate, *CI* Confidence interval, *AAPC* Average annual percent change.

From 2015 to 2019, a total of 146,184 cases of kidney cancer were reported in the US. The majority of cases were among NHWs (64.82%) and individuals aged 55–69 years (43.03%). The ASIRs per 100,000 population during this period were 22.66 (22.51, 22.81) for men and 11.35 (11.26, 11.45) for women. Among all races and ethnicities, NHB men had the highest ASIR (25.95 [25.43, 26.48]). There was a significant increase in ASIR over the 2015–2019 period in both sexes, with an AAPC of 1.20% (0.94, 1.82) in men and 0.76% (0.44, 1.03) in women (Table S4).

##### Men

A total of 291,701 cases of kidney cancer were reported in men from 2000 to 2019. The most common subtype was RCC (90.97%). The majority of cases were NHWs (70.15%) and between 55 and 69 years (26.66%). NHB men had the highest ASIR (23.77 [23.49, 24.04]). Individuals in the 70 to 84 age group had the highest ASIR (90.98 [90.35, 91.61]). Those below 39 years had the highest AAPC (3.34% [2.99, 3.74]) (Table 3).

Of all the reported cases, 41,916 (14.37%) were Hispanics. The majority of them were between 55 and 69 years (39.87%). The overall ASIR per 100,000 population was 21.30 (21.07, 21.52). Those aged 70–84 years had the highest ASIR (97.07 [95.05, 99.11]). There was a significant increase in ASIR of Hispanic cases over 2000–2019 (2.07% [1.72, 2.55]) (Table 3).

NHBs consisted 10.81% of the men with kidney cancer. The majority of NHBs were between 55 and 69 years (45.65%). The overall ASIR per 100,000 population was 23.77 (23.49, 24.04) and the cases between 70 and 84 years had the highest ASIR (97.53 [95.17, 99.93]). The overall AAPC in NHBs was 2.00% (1.67, 2.48) (Table 3).

There were 204,633 reported cases of NHWs, consisting of 70.15% of the men with kidney cancer. Most cases were between 55 and 69 years (42.39%). The overall ASIR per 100,000 population was 21.14 (21.05, 21.23) with cases between 70 and 84 years having the highest ASIR (93.13 [92.40, 93.86]). There was a significant increase in overall ASIR over 2000–2019 in NHW men (AAPC: 1.72% [1.59, 1.87]) (Table 3).

##### Women

Between 2000 and 2019, a total of 171,700 cases of kidney cancer were reported in women. The most common tumor subtype was RCC (89.20%). The majority of them were among NHWs (67.21%) and those aged 55–69 years (37.58%). The overall ASIR per 100,000 population was 10.42 (10.37, 10.47). Hispanic women had the greatest ASIR (12.07 [11.92, 12.21]). There was a significant rise in the ASIR from 2000 to 2019 (AAPC: 1.70% [1.50, 1.97]). All age groups experienced a significant increase in ASIR with those < 39 years exhibited the highest increase (AAPC: 3.15%; [2.60, 3.81]) (Table 3).

Among all reported cases, 28,182 (16.41%) were among Hispanic individuals. The majority of cases were between 50 and 69 years (37.07%). The overall ASIR per 100,000 population was 12.07 (11.92, 12.21). Individuals between 70 and 84 years had the highest ASIR (50.27 [49.04, 51.52]). There was a significant increase in the ASIR of Hispanic women over 2000–2019 (AAPC: 2.20% [1.59, 3.06]) (Table 3).

NHBs constituted 11.98% of the cases of kidney cancer in women. A significant portion of NHB cases fell within the 55 to 69-year age group (40.65%). The overall ASIR per 100,000 population for NHB women was 11.95 (11.79, 12.12). There was a significant increase in ASIR over 2000–2019 (AAPC: 1.98% [1.60, 2.49]) (Table 3).

The majority of NHW cases were in the 55 to 69 (37.14%) age group. The overall ASIR per 100,000 population was 10.40 (10.34, 10.46). Over the 2000–2019 period, there was a significant increase in the overall ASIR for NHW women (AAPC: 1.54% [1.34, 1.82]) (Table 3).

#### Age and sex patterns

Following a decrease in the 0–4 age group, the incidence rates increased with age and peaked at the 75–79 age group. The incidence rates were higher among men. The incident cases were highest in the 65–69 age group for both men and women (Figure S5).

### Histopathological variants in kidney cancers

#### RCC

Over 2000–2019, there were a total of 418,526 cases of RCC in the US across all age groups. The majority of cases were in men (63.41%), NHWs (69.03%), and individuals aged 55–69 years (41.85%). The ASIR per 100,000 population was 18.75 (18.68, 18.83) for men and 9.29 (9.24, 9.34) for women. The AAPC for men and women were 2.30% (2.20, 2.44) and 2.34% (2.14, 2.60), respectively. NHB men had the highest ASIR (21.16 [20.90, 21.42]). Hispanic men experienced the greatest increase over 2000–2019 (AAPC: 2.58% [2.42, 2.86]) (Table 4, Figure S6, Figure S7, and Figure S8). The ASIRs and changes over 2000–2019 for men and women are provided in Appendix 1.

**Table 4:** Counts and age-standardized rate of renal cell carcinoma incidence per 100,000 and average annual percent change from 2000 to 2019 in the United States, by age, sex, and race.

Age group (years)	Men			Women		
	Case (%)	ASIR (95% CI)	AAPC (95% CI)	Case (%)	ASIR (95% CI)	AAPC (95% CI)
All race/ethnicities						
All	265,367 (63.41)	18.75 (18.68, 18.83)	2.3 (2.2, 2.44)	153,159 (36.59)	9.29 (9.24, 9.34)	2.34 (2.14, 2.6)
0–39	10,730 (2.56)	1.38 (1.35, 1.41)	4.57 (4.18, 5.07)	7631 (1.82)	0.98 (0.96, 1)	4.63 (3.9, 5.68)
40–54	57,803 (13.81)	18.64 (18.49, 18.79)	3.06 (2.65, 3.54)	30,874 (7.38)	9.71 (9.6, 9.82)	3.13 (2.7, 3.61)
55–69	114,933 (27.46)	55.92 (55.59, 56.24)	1.95 (1.8, 2.22)	60,220 (14.39)	26.64 (26.43, 26.85)	2 (1.82, 2.27)
70–84	72,733 (17.38)	81.49 (80.9, 82.08)	2.08 (1.9, 2.3)	45,742 (10.93)	38.92 (38.57, 39.28)	2.08 (1.76, 2.4)
+ 85	9168 (2.19)	57.44 (56.27, 58.63)	1.28 (0.66, 2.37)	8692 (2.08)	26.54 (25.99, 27.1)	1.4 (0.8, 2.24)
Hispanic						
All	38,496 (60.16)	19.53 (19.32, 19.74)	2.58 (2.42, 2.86)	25,491 (39.84)	10.98 (10.84, 11.11)	2.43 (2.08, 2.89)
0–39	2627 (4.11)	1.26 (1.22, 1.31)	6.81 (5.28, 8.79)	2127 (3.32)	1.08 (1.03, 1.12)	5.77 (4.62, 7.33)
40–54	11,075 (17.31)	18.63 (18.28, 18.98)	2.84 (2.56, 3.19)	6927 (10.83)	11.66 (11.38, 11.93)	3.46 (2.87, 4.22)
55–69	15,707 (24.55)	57.57 (56.67, 58.49)	2.44 (1.9, 3.24)	9857 (15.40)	31.97 (31.34, 32.62)	2.4 (1.96, 2.92)
70–84	8199 (12.81)	88.43 (86.51, 90.38)	2.32 (2.05, 2.75)	5809 (9.08)	45.48 (44.31, 46.66)	1.25 (0.61, 2.04)
+ 85	888 (1.39)	64.5 (60.33, 68.89)	0.54 (-1.4, 3.4)	771 (1.20)	30.14 (28.05, 32.35)	1.57 (0.13, 3.58)
NHB						
All	28,250 (61.09)	21.16 (20.9, 21.42)	2.41 (2.04, 2.96)	17,994 (38.91)	10.44 (10.29, 10.6)	2.42 (1.75, 3.38)
0–39	1377 (2.98)	1.53 (1.45, 1.61)	2.13 (0.63, 4.17)	979 (2.12)	0.98 (0.92, 1.04)	2.09 (0.6, 3.78)
40–54	7346 (15.89)	21.57 (21.08, 22.07)	2.81 (2.15, 3.55)	3899 (8.43)	10.13 (9.81, 10.45)	1.88 (0.96, 2.93)
55–69	13,160 (28.46)	67.51 (66.35, 68.68)	2.15 (1.58, 3.02)	7641 (16.52)	31.39 (30.69, 32.1)	3.03 (2.54, 3.74)
70–84	5867 (12.69)	86.11 (83.89, 88.36)	2.66 (2.14, 3.17)	4712 (10.19)	44.23 (42.97, 45.51)	2.69 (1.52, 4.5)
+ 85	500 (1.08)	52.37 (47.88, 57.17)	0.2 (-1.93, 2.86)	763 (1.65)	31.58 (29.38, 33.9)	1.02 (-1.11, 3.56)
NHW						
All	186,071 (64.40)	19.12 (19.03, 19.21)	2.26 (2.1, 2.5)	102,848 (35.60)	9.26 (9.2, 9.32)	2.18 (2.02, 2.4)
0–39	5984 (2.07)	1.48 (1.44, 1.52)	4.34 (3.87, 4.86)	4073 (1.41)	1.02 (0.99, 1.05)	4.49 (3.56, 5.69)
40–54	36,256 (12.55)	19.04 (18.84, 19.24)	3.07 (2.6, 3.59)	18,493 (6.40)	9.68 (9.54, 9.83)	3.17 (2.83, 3.54)
55–69	80,728 (27.94)	56.47 (56.08, 56.86)	1.81 (1.65, 2.07)	40,021 (13.85)	26.27 (26.01, 26.53)	1.67 (1.52, 1.87)
70–84	55,674 (19.27)	83.23 (82.54, 83.93)	2.09 (1.93, 2.3)	33,394 (11.56)	38.9 (38.48, 39.32)	1.97 (1.61, 2.32)
+ 85	7429 (2.57)	59.09 (57.75, 60.45)	1.56 (1.02, 2.25)	6867 (2.38)	26.42 (25.8, 27.05)	1.78 (1.3, 2.41)

*NHW* Non-Hispanic White, *NHB* Non-Hispanic Black, *ASIR* Age-standardized incidence rate, *CI* Confidence interval, *AAPC* Average annual percent change.

The incidence rate of RCC between 2000 and 2019 increased with advancing age up to 75–79 age group and decreased then after. It was higher in men than women after the 30–34 age group. In terms of incident cases, it increased with age and reached to the highest level in the 65–69 years (Figure S9).

#### Nephroblastoma

Over 2000–2019, 4,645 cases of nephroblastoma in all age groups were reported in the US. The majority of them were women (53.89%), NHWs (51.07%), and aged between 0 and 4 years (70.78%). The ASIR per 100,000 population was 0.15 (0.14, 0.16) for men and 0.18 (0.17, 0.19) for women. NHB women had the highest ASIR (0.24 [0.22, 0.26]). None of the sexes exhibited a substantial alternation in ASIRs over 2000–2019 (Table 5, Figure S10, Figure S11, and Figure S12). The ASIRs and changes over 2000–2019 for men and women are provided in Appendix 1.

**Table 5:** Counts and age-standardized rate of nephroblastoma incidence per 100,000 and average annual percent change from 2000 to 2019 in the United States, by age, sex, and race.

Age group (years)	Men			Women		
	Case (%)	ASIR (95% CI)	AAPC (95% CI)	Case (%)	ASIR (95% CI)	AAPC (95% CI)
All race/ethnicities						
All	2142 (46.11)	0.15 (0.14, 0.16)	− 0.16 (− 1.22, 0.92)	2503 (53.89)	0.18 (0.17, 0.19)	0.06 (− 0.68, 0.8)
0–4	1573 (33.86)	1.57 (1.49, 1.64)	− 0.36 (− 1.41, 0.66)	1715 (36.92)	1.78 (1.7, 1.87)	− 0.7 (− 1.75, 0.34)
5–9	417 (8.98)	0.41 (0.37, 0.45)	2.04 (0.47, 3.79)	574 (12.36)	0.59 (0.55, 0.64)	0.45 (− 2.27, 4.61)
10–14	53 (1.14)	0.05 (0.04, 0.07)	N/A	79 (1.70)	0.08 (0.06, 0.1)	1.14 (− 2.22, 4.83)
15+	99 (2.13)	0.01 (0.01, 0.01)	− 4.51 (− 8.17, − 1.24)	135 (2.91)	0.01 (0.01, 0.01)	0.13 (− 4, 4.43)
Hispanic						
All	558 (45.37)	0.12 (0.11, 0.13)	0.13 (− 1.86, 2.3)	672 (54.63)	0.15 (0.14, 0.16)	0.45 (− 1.19, 2.26)
0–4	412 (33.50)	1.24 (1.12, 1.37)	0.17 (− 2.36, 2.81)	503 (40.89)	1.58 (1.44, 1.72)	− 0.57 (− 2.36, 1.27)
5–9	115 (9.35)	0.36 (0.3, 0.43)	1.15 (− 3.53, 6.94)	135 (10.89)	0.44 (0.37, 0.52)	3.55 (− 0.18, 8.72)
10–14	9 (0.73)	0.03 (0.01, 0.05)	N/A	14 (1.14)	0.05 (0.03, 0.08)	N/A
15+	22 (1.79)	0.01 (0, 0.01)	N/A	20 (1.63)	0.01 (0, 0.01)	N/A
NHB						
All	361 (45.47)	0.19 (0.17, 0.21)	0.81 (− 1.58, 3.36)	433 (54.53)	0.24 (0.22, 0.26)	− 0.08 (− 2.58, 2.54)
0–4	262 (33.00)	2 (1.77, 2.26)	− 0.13 (− 3.18, 2.92)	264 (33.25)	2.09 (1.84, 2.36)	− 0.89 (− 4.55, 2.94)
5–9	74 (9.32)	0.55 (0.43, 0.69)	4.58 (0.62, 9.92)	131 (16.50)	1 (0.84, 1.19)	1 (− 2.46, 4.77)
10–14	10 (1.26)	0.07 (0.03, 0.13)	N/A	21 (2.64)	0.15 (0.09, 0.23)	N/A
15+	15 (1.89)	0.01 (0.01, 0.02)	N/A	17 (2.14)	0.01 (0.01, 0.02)	N/A
NHW						
All	1105 (46.59)	0.17 (0.16, 0.18)	0.11 (− 1.32, 1.5)	1267 (53.41)	0.2 (0.19, 0.21)	0.11 (− 1.14, 1.34)
0–4	808 (34.06)	1.76 (1.64, 1.89)	− 0.05 (− 1.98, 1.82)	848 (35.74)	1.95 (1.82, 2.08)	− 0.5 (− 1.65, 0.61)
5–9	211 (8.90)	0.44 (0.39, 0.51)	2.09 (0.05, 4.27)	285 (12.02)	0.63 (0.56, 0.71)	− 2.1 (− 6.07, 1.88)
10–14	31 (1.31)	0.06 (0.04, 0.09)	N/A	40 (1.69)	0.08 (0.06, 0.11)	N/A
15+	55 (2.32)	0.01 (0.01, 0.01)	N/A	94 (3.96)	0.01 (0.01, 0.02)	0.92 (− 3.16, 5.02)

*NHW* Non-Hispanic White, *NHB* Non-Hispanic Black, *ASIR* Age-standardized incidence rate, *CI* Confidence interval, *AAPC* Average annual percent change, *N/A* Not available.

The highest incidence rates were in those aged < 4 years and it decreased with advancing age. There were not substantial differences between males and females in terms of incidence rates. The highest number of incident cases were among females and in the 0–4-year age group (Figure S13).

#### Sarcoma

From 2000 to 2019, there were 556 sarcoma cases in all age groups in the US. The majority of cases were men (63.43%), NHWs (64.03%), and under 39 years (46.82%). The ASIR per 100,000 population was 0.03 (0.02, 0.03) for men and 0.01 (0.01, 0.02) for women. NHW men had the highest ASIR (0.03 [0.02, 0.03]). There was a significant decrease in the ASIR for men (AAPC: − 2.66% [− 4.70, − 0.21]) (Table 6, Figure S14, Figure S15, and Figure S16). The ASIRs and changes over 2000–2019 for men and women are provided in Appendix 1.

**Table 6:** Counts and age-standardized rate of sarcoma incidence per 100,000 and average annual percent change from 2000 to 2019 in the United States, by age, sex, and race.

Age group (years)	Men			Women		
	Case (%)	ASIR (95% CI)	AAPC (95% CI)	Case (%)	ASIR (95% CI)	AAPC (95% CI)
All race/ethnicities						
All	359 (63.43)	0.03 (0.02, 0.03)	− 2.66 (− 4.7, − 0.21)	207 (36.57)	0.01 (0.01, 0.02)	− 1.73 (− 4.33, 0.82)
0–39	172 (30.39)	0.02 (0.02, 0.02)	− 2.62 (− 6.52, 1.52)	93 (16.43)	0.01 (0.01, 0.01)	N/A
40–54	44 (7.77)	0.01 (0.01, 0.02)	N/A	31 (5.48)	0.01 (0.01, 0.01)	N/A
55–69	82 (14.49)	0.04 (0.03, 0.05)	− 6.65 (− 9.1, − 2.64)	43 (7.60)	0.02 (0.01, 0.03)	N/A
70–84	50 (8.83)	0.06 (0.04, 0.07)	− 1.49 (− 6.32, 3.58)	29 (5.12)	0.03 (0.02, 0.04)	N/A
+ 85	11 (1.94)	0.07 (0.03, 0.12)	N/A	11 (1.94)	0.03 (0.02, 0.06)	N/A
Hispanic						
All	74 (62.71)	0.02 (0.02, 0.03)	− 1.81 (− 5.01, 1.58)	44 (37.29)	0.01 (0.01, 0.02)	N/A
0–39	48 (40.68)	0.02 (0.01, 0.02)	N/A	27 (22.88)	0.01 (0.01, 0.02)	N/A
40–54	11 (9.32)	0.02 (0.01, 0.03)	N/A	5 (4.24)	0.01 (0, 0.02)	N/A
55–69	10 (8.47)	0.04 (0.02, 0.07)	N/A	9 (7.63)	0.03 (0.01, 0.06)	N/A
70–84	5 (4.24)	0.05 (0.02, 0.12)	N/A	3 (2.54)	0.02 (0, 0.07)	N/A
+ 85	0 (0.00)	0 (0, 0.27)	N/A	0 (0.00)	0 (0, 0.14)	N/A
NHB						
All	37 (66.07)	0.02 (0.01, 0.03)	N/A	19 (33.93)	0.01 (0.01, 0.02)	N/A
0–39	26 (46.43)	0.03 (0.02, 0.04)	N/A	9 (16.07)	0.01 (0, 0.02)	N/A
40–54	3 (5.36)	0.01 (0, 0.03)	N/A	2 (3.57)	0.01 (0, 0.02)	N/A
55–69	8 (14.29)	0.04 (0.02, 0.08)	N/A	6 (10.71)	0.02 (0.01, 0.05)	N/A
70–84	0 (0.00)	0 (0, 0.06)	N/A	2 (3.57)	0.02 (0, 0.07)	N/A
+ 85	0 (0.00)	0 (0, 0.39)	N/A	0 (0.00)	0 (0, 0.15)	N/A
NHW						
All	229 (64.33)	0.03 (0.02, 0.03)	− 2.74 (− 5.6, 0.56)	127 (35.67)	0.01 (0.01, 0.02)	-3.7 (-8.03, -0.19)
0–39	87 (24.44)	0.02 (0.02, 0.03)	− 2.51 (− 8.59, 3.12)	47 (13.20)	0.01 (0.01, 0.02)	N/A
40–54	27 (7.58)	0.01 (0.01, 0.02)	N/A	18 (5.006)	0.01 (0.01, 0.02)	N/A
55–69	60 (16.85)	0.04 (0.03, 0.05)	− 5.17 (− 8.94, − 1.67)	28 (7.87)	0.02 (0.01, 0.03)	N/A
70–84	44 (12.36)	0.07 (0.05, 0.09)	N/A	23 (6.46)	0.03 (0.02, 0.04)	N/A
+ 85	11 (3.09)	0.09 (0.04, 0.16)	N/A	11 (3.09)	0.04 (0.02, 0.08)	N/A

*NHW* Non-Hispanic White, *NHB* Non-Hispanic Black, *ASIR* Age-standardized incidence rate, *CI* Confidence interval, *AAPC* Average annual percent change, *N/A* Not available.

There were fluctuations in the incidence rates of sarcoma among men and women. While, the 0–4-year age group had the largest incidence rate. There were not substantial differences between men and women in incidence rates of sarcoma. In terms of incident cases, those < 4 years had the highest number of cases (Figure S17).

#### Neuroendocrine tumor

Over 2000–2019, there were a total of 300 cases of neuroendocrine tumor in the US across all age groups. The majority of cases were observed in men (54.76%), NHWs (74.33%), and between 70 and 84 years (33.67%). The ASIRs per 100,000 population were 0.01 (0.01, 0.01) for men and women. None of the age groups experienced significant alternation in ASIRs over 2000–2019 (Table 7, Figure S18, Figure S19, and Figure S20). The ASIRs and changes over 2000–2019 for men and women are provided in Appendix 1.

**Table 7:** Counts and age-standardized rate of neuroendocrine tumor incidence per 100,000 and average annual percent change from 2000 to 2019 in the United States, by age, sex, and race.

Age group (years)	Men			Women		
	Case (%)	ASIR (95% CI)	AAPC (95% CI)	Case (%)	ASIR (95% CI)	AAPC (95% CI)
All race/ethnicities						
All	164(54.67)	0.01 (0.01, 0.01)	0.74 (− 2.27, 4.26)	136(54.67)	0.01 (0.01, 0.01)	1.53 (− 2.37, 6.42)
0–39	13 (4.33)	0 (0, 0)	N/A	13 (4.33)	0 (0, 0)	N/A
40–54	29 (9.67)	0.01 (0.01, 0.01)	N/A	35 (11.67)	0.01 (0.01, 0.02)	N/A
55–69	47 (15.67)	0.02 (0.02, 0.03)	3.04 (− 3.55, 13.66)	40 (13.33)	0.02 (0.01, 0.02)	N/A
70–84	62 (20.67)	0.07 (0.05, 0.09)	N/A	39 (13.00)	0.03 (0.02, 0.04)	N/A
+ 85	13 (4.33)	0.08 (0.04, 0.14)	N/A	9 (3.00)	0.03 (0.01, 0.05)	N/A
Hispanic						
All	13(50.00)	0.01 (0, 0.01)	N/A	13 (50.00)	0.01 (0, 0.01)	N/A
0–39	2 (7.69)	0 (0, 0)	N/A	2 (7.69)	0 (0, 0)	N/A
40–54	3 (11.54)	0.01 (0, 0.01)	N/A	2 (7.69)	0 (0, 0.01)	N/A
55–69	4 (15.38)	0.01 (0, 0.04)	N/A	3 (11.54)	0.01 (0, 0.03)	N/A
70–84	4 (15.38)	0.05 (0.01, 0.12)	N/A	4 (15.38)	0.03 (0.01, 0.08)	N/A
+ 85	0 (0.00)	0 (0, 0.27)	N/A	2 (7.69)	0.08 (0.01, 0.28)	N/A
NHB						
All	15 (45.45)	0.01 (0.01, 0.02)	N/A	18 (54.55)	0.01 (0.01, 0.02)	N/A
0–39	2 (6.06)	0 (0, 0.01)	N/A	2 (6.06)	0 (0, 0.01)	N/A
40–54	2 (6.06)	0.01 (0, 0.02)	N/A	6 (18.18)	0.02 (0.01, 0.03)	N/A
55–69	5 (15.15)	0.03 (0.01, 0.06)	N/A	7 (21.21)	0.03 (0.01, 0.06)	N/A
70–84	5 (15.15)	0.07 (0.02, 0.16)	N/A	2 (6.06)	0.02 (0, 0.07)	N/A
+ 85	1 (3.03)	0.1 (0, 0.58)	N/A	1 (3.03)	0.04 (0, 0.23)	N/A
NHW						
All	127(56.95)	0.01 (0.01, 0.02)	2.19 (− 2.06, 7.29)	96 (43.05)	0.01 (0.01, 0.01)	− 1.03 (− 9.06, 9.02)
0–39	8 (3.59)	0 (0, 0)	N/A	7 (3.14)	0 (0, 0)	N/A
40–54	20 (8.97)	0.01 (0.01, 0.02)	N/A	25 (11.21)	0.01 (0.01, 0.02)	N/A
55–69	36 (16.14)	0.03 (0.02, 0.03)	N/A	27 (12.11)	0.02 (0.01, 0.03)	N/A
70–84	51 (22.87)	0.08 (0.06, 0.1)	N/A	32 (14.35)	0.04 (0.02, 0.05)	N/A
+ 85	12 (5.38)	0.1 (0.05, 0.17)	N/A	5 (2.24)	0.02 (0.01, 0.04)	N/A

*NHW* Non-Hispanic White, *NHB* Non-Hispanic Black, *ASIR* Age-standardized incidence rate, *CI* Confidence interval, *AAPC* Average annual percent change, *N/A* Not available.

The incidence rates of neuroendocrine tumors generally increased with advancing age, while there were fluctuations. It peaked at the 80–84-year age group for both men and women. There were not substantial changes in incidence rates between males and females. The highest number of incident cases were in 70–74 and 50–54 age groups for males and females, respectively (Figure S21).

### Renal pelvis

#### Overall incidence

Between 2000 and 2019, there were a total of 27,080 cases of renal pelvis cancer across all age groups in the US. The majority of them were among NHWs (81.64%) and individuals aged 70–84 years (50.19%). The ASIR per 100,000 population was 1.22 (1.20, 1.24) for men and 0.67 (0.66, 0.69) for women. NHW men had the highest ASIR (1.38 [1.35, 1.40]) (Table 8, Figure S22, Figure S23, and Figure S24).

**Table 8:** Counts and age-standardized rate of renal pelvis cancer incidence per 100,000 and average annual percent change from 2000 to 2019 in the United States by age, sex, and race.

Age group (years)	Men			Women		
	Case (%)	ASIR (95% CI)	AAPC (95% CI)	Case (%)	ASIR (95% CI)	AAPC (95% CI)
All race/ethnicities						
All	15,696 (57.96)	1.22 (1.2, 1.24)	− 0.5 (− 1.46, 0.46)	11,384 (42.04)	0.67 (0.66, 0.69)	− 0.12 (− 0.55, 0.33)
0–39	106 (0.93)	0.01 (0.01, 0.02)	− 2.94 (− 7.15, 0.61)	52 (0.19)	0.01 (0.01, 0.01)	− 0.72 (− 4.46, 3.25)
40–54	1217 (4.49)	0.39 (0.36, 0.41)	− 2.19 (− 3.33, − 1.09)	622 (2.30)	0.19 (0.18, 0.21)	− 0.71 (− 1.96, 0.54)
55–69	5087 (18.79)	2.51 (2.44, 2.58)	− 1.65 (− 2.35, − 0.9)	2816 (10.40)	1.25 (1.21, 1.3)	− 1.9 (− 2.73, − 1.01)
70–84	7612 (28.11)	8.66 (8.47, 8.86)	− 0.11 (− 0.92, 0.73)	5980 (22.08)	5.04 (4.91, 5.17)	0.28 (− 0.41, 1.02)
+ 85	1674 (6.18)	10.49 (9.99, 11)	1.23 (0.04, 2.6)	1914 (7.07)	5.84 (5.59, 6.11)	1.47 (0.23, 2.91)
Hispanic						
All	1334 (60.04)	0.89 (0.84, 0.94)	− 0.86 (− 2.3, 0.89)	888 (39.96)	0.47 (0.44, 0.5)	− 0.24 (− 1.67, 1.47)
0–39	25 (1.13)	0.01 (0.01, 0.02)	N/A	13 (0.59)	0.01 (0, 0.01)	N/A
40–54	162 (7.29)	0.27 (0.23, 0.32)	− 3.55 (− 5.88, 0.47)	68 (3.06)	0.11 (0.09, 0.15)	− 0.89 (− 5.21, 4.04)
55–69	457 (20.57)	1.72 (1.56, 1.88)	− 1.64 (− 3.99, 1.26)	245 (11.03)	0.81 (0.71, 0.92)	− 1.17 (− 3.15, 1.16)
70–84	586 (26.37)	6.51 (5.99, 7.06)	− 1 (− 3.14, 1.53)	444 (19.98)	3.53 (3.21, 3.88)	− 0.14 (− 1.67, 1.66)
+ 85	104 (4.68)	7.55 (6.17, 9.15)	N/A	118 (5.31)	4.61 (3.82, 5.52)	N/A
NHB						
All	688 (51.96)	0.6 (0.55, 0.65)	− 0.66 (− 2.16, 0.99)	636 (48.04)	0.4 (0.37, 0.43)	0.03 (− 1.9, 2.21)
0–39	10 (0.76)	0.01 (0.01, 0.02)	N/A	10 (0.76)	0.01 (0, 0.02)	N/A
40–54	106 (8.01)	0.31 (0.25, 0.37)	− 0.75 (− 3.85, 2.56)	71 (5.36)	0.18 (0.14, 0.23)	N/A
55–69	293 (22.13)	1.53 (1.36, 1.71)	− 2.18 (− 4.69, 0.56)	201 (15.18)	0.83 (0.72, 0.95)	− 1.9 (− 5.98, 3.05)
70–84	251 (18.96)	3.81 (3.35, 4.32)	0.06 (− 2.05, 2.52)	289 (21.83)	2.75 (2.44, 3.08)	0.34 (− 1.47, 2.41)
+ 85	28 (2.11)	2.93 (1.95, 4.24)	N/A	65 (4.91)	2.69 (2.08, 3.43)	N/A
NHW						
All	12,880 (58.26)	1.38 (1.35, 1.4)	− 0.25 (− 1.55, 1.04)	9227 (41.74)	0.76 (0.74, 0.77)	0.09 (− 0.53, 0.75)
0–39	63 (0.28)	0.02 (0.01, 0.02)	N/A	24 (0.11)	0.01 (0, 0.01)	N/A
40–54	891 (4.03)	0.45 (0.43, 0.49)	− 2.17 (− 3.43, − 1.07)	431 (1.95)	0.22 (0.2, 0.24)	− 0.11 (− 1.83, 1.57)
55–69	4065 (18.39)	2.86 (2.78, 2.95)	− 1.45 (− 2.19, − 0.67)	2205 (9.97)	1.45 (1.39, 1.51)	− 2.08 (− 2.97, − 1.17)
70–84	6403 (28.96)	9.66 (9.43, 9.9)	0.12 (− 0.78, 1.06)	4932 (22.31)	5.65 (5.49, 5.81)	0.65 (− 0.1, 1.45)
+ 85	1458 (6.60)	11.6 (11.01, 12.21)	1.81 (− 0.33, 4.27)	1635 (7.40)	6.29 (5.99, 6.6)	1.79 (0.7, 3.05)

*NHW* Non-Hispanic White, *NHB* Non-Hispanic Black, *ASIR* Age-standardized incidence rate, *CI* Confidence interval, *AAPC* Average annual percent change, *N/A* Not available.

From 2015 to 2019, a total of 7,574 cases of renal pelvis cancer were reported. The majority of cases were among NHW (78.10%), and most cases occurred in individuals aged 55 to 69 years (50.05%). The ASIR per 100,000 population during this period was 1.14 (1.11, 1.18) for men and 0.66 (0.64, 0.68) for women. There was a significant decrease in ASIR over the 2015–2019 period in men (AAPC: − 1.31% [− 5.40, − 0.49]) (Table S5).

##### Men

Over 2000–2019, a total of 15,696 (57.96%) cases of renal pelvis cancer were diagnosed in men. Most cases were at the age of 70 to 84 years (48.50%) and among NHWs (82.06%). Cases over 85 years had the highest ASIR among all age groups (10.49 [9.99, 11.00]). Among all age groups cases aged 40–54 and 55–69 years had a significant decline in ASIRs, whereas those > 85 years experienced a significant increase over 2000–2019 (AAPC: 1.23%; [0.04, 2.60]) (Table 8).

Of all the reported cases 8.50% were among Hispanic men. The majority of cases were between 70 and 84 years (43.92%). The overall ASIR per 100,000 population was 0.89 (0.84, 0.94). Also, of all men, 688 (4.38%) of cases were NHBs. The majority of cases (42.59%) were between 55 and 69 years. The ASIR per 100,000 population was 0.60 (0.55, 0.65). The majority of NHW men were between 70 and 84 years (49.71%). The overall ASIR per 100,000 population was 1.38 (1.35, 1.40) (Table 8).

##### Women

A total of 11,384 cases of renal pelvis cancer were reported over 2000–2019 among women in the US. The majority of cases occurred in NHWs (81.05%) and between 70 and 84 years (52.53%). Cases over 85 years had the highest ASIR among all age groups (5.84 [5.59, 6.11]). Of all the age groups, cases between 55 and 69 years showed a significant decrease in ASIR (AAPC: − 1.90%; [− 2.73, − 1.01]) and individuals > 85 years had a significant increase between 2000 and 2019 (1.47% [0.23, 2.91]) (Table 8).

Of all the women, 7.80% were Hispanics. The majority of cases were between 70 and 84 years (50.00%). The ASIR per 100,000 population in Hispanic women was 0.47 (0.44, 0.50). Among women, 5.59% of reported cases were NHBs. The majority of cases (45.44%) were between 70 and 84 years. The ASIR per 100,000 population in NHB women was 0.40 (0.37, 0.43). The majority of NHW cases were between 70 and 84 years (53.45%). The ASIR per 100,000 population in NHW women was 0.76 (0.74, 0.77) (Table 8).

#### Age and sex patterns

The incidence rates increased with ageing and peaked at the 80–84-year age group for both men and women. There was a similar pattern for incident cases and they peaked at the 75–79-year age group for both men and women. Men had higher incidence rates than women in almost all age groups (Figure S25).

## Discussion

### Summary of findings

From 2000 to 2019, the ASIR per 100,000 population for kidney cancer were almost double in men compared to women (20.72 vs. 10.42 per 100,000). For renal pelvis cancer over the same period, the ASIRs were 1.22 and 0.67 for men and women, respectively. The most common subtype, RCC, showed higher rates in men. Nephroblastoma displayed distinct patterns, with the highest ASIR among NHB women. For renal pelvis cancer, the ASIR per 100,000 individuals remained relatively constant over 2000–2019. By age, individuals aged 70–84 years consistently had the highest ASIR for kidney cancer, while those 85 years or older had the highest ASIR for renal pelvis cancers. By sex, the ASIRs for both kidney and renal pelvis cancers were higher in men. By race, the majority of cases were among NHW individuals, with NHB men having the highest ASIR for renal and pelvis cancers. In the 2015–2019 period, there were general similar patterns to the period of 2000–2019. The COVID-19 led to a significant decrease in ASIRs of renal and pelvis cancers.

### Histological subtypes

Regarding temporal trends, it is noteworthy to emphasize a significant upswing in ASIR for both kidney and renal pelvis cancers in men from 2015 to 2019. Consistent with prior research, other studies affirm these findings, indicating an escalation in incidence rates for all four genitourinary cancers, especially advanced-stage prostate cancer, across all racial and ethnic groups in over the period 2015–2019, except for bladder cancer, which witnessed an increase solely among American Indian and Alaska Native individuals^11^. Existing literature also posits that the surge in kidney cancer incidence is partly attributable to the obesity epidemic^28^ and the growing prevalence of type 2 diabetes^29^. The Global Burden of Diseases Study 2019 have reported an elevation in the ASIR for kidney, bladder, prostate, and testicular cancers over the period from 1990 to 2019 by 29.1%, 4.0%, 22.0%, and 45.5%, respectively^30^.

The analysis of kidney cancer incidence trends based on histological subtypes revealed notable patterns over the 2000–2019 period. The most common subtype, RCC, demonstrated a general rise in incidence rates, consistently presenting higher rates in men compared to women. This finding is congruent with a study by Palumbo et al. based on the SEER database, demonstrating that regarding RCC, the male incidence rate per 100,000 person years was double that of females (15.5 vs. 7.7)^31^. Several risk factors, such as being male, chronic kidney disease, smoking, hypertension, and high body mass index contribute to an increased susceptibility to RCC^32^. Hence, the rationale behind our observations might be attributed to the presence of certain risk factors within our sampled population. The age-specific analysis for individuals with RCC indicated that cases between 70 and 84 years had the highest ASIR in both men and women. One potential hypothesis is that this age group may be more susceptible to certain risk factors associated with kidney and renal pelvis cancers, such as chronic kidney disease, which is more common in individuals 65 years or older^33^.

Nephroblastoma displayed distinct patterns with a higher incidence rate among NHB women and a peak in the 0–4 age group. This finding is similar to what previous literature has stated, reporting that in the US, the majority of nephroblastoma or Wilms tumor cases are identified before the age of five years, constituting two-thirds of diagnoses^34^. Our results demonstrate that NHB females had the highest ASIR (0.24 [0.22, 0.26] per 100,000). Similar to our findings, a study by Geris et al. in 2020 regarding racial differences in the incidence of pediatric embryonal tumors in the US, reported that most tumors showed lower occurrence rates in non-white children when compared to NHW children. Nevertheless, NHB children exhibited a higher incidence of Wilms tumor than their NHW counterparts^35^. Wilms tumor exhibits a strong genetic component, as evidenced by genome-wide association studies identifying specific genetic variations associated with its susceptibility^36^. These variations are more prevalent in individuals of African ancestry compared to those of European descent. Furthermore, mutations in *WT1* and *WT2* genes, linked to specific syndromes and congenital anomalies, occur more frequently in black children than in white children, suggesting a predisposition to Wilms tumor development among the former group^37,38^. Less common forms of kidney cancer, such as neuroendocrine tumors and sarcoma, have not been extensively documented in recent reports. Consequently, these discoveries can serve as valuable information for the development of health strategies and the planning of effective management approaches for individuals affected by these types of kidney cancers.

Our findings indicate that ASIRs for renal pelvis cancer per 100,000 individuals from 2000 to 2019 were 1.22 and 0.67 for males and females, respectively. Also, there were not significant changes in ASIRs of renal pelvis cancer over 2000–2019. Additionally, in terms of racial differences, our results revealed that NHWs had the highest ASIR compared to NHBs and Hispanics. A study by Noone et al. analyzing the SEER data for 1992–2013^39^, highlighted that renal pelvis cancer consistently displayed a lower incidence rate compared to kidney cancer and has maintained relative stability in incidence rates since 1992. The study also affirmed, in alignment with our results, that while the incidence pattern is comparable between men and women, women generally exhibited a lower rate of incidence^39^. Furthermore, it reiterated a similarity to our findings, noting that the highest incidence rates for renal pelvis cancer are observed among individuals of white ethnicity, irrespective of sex.

### Incidence of renal and pelvis cancers by age and sex

The incidence trends of kidney cancer from 2000 to 2019 revealed that individuals aged 70–84 years consistently had the highest ASIR in both men and women while individuals 85 years or older had the highest ASIR regarding renal pelvis cancers. The research conducted by Noone et al. on cancer incidence trends by subtype similarly illustrated the trend of a rising incidence rate with age for both kidney and renal pelvis cancers^39^. Previous literature indicated that RCC typically manifests predominantly in individuals aged between their sixth and eighth decades, with the median age at diagnosis around 64 years; and also stated that the occurrence of RCC is uncommon in patients < 40 years and is rare in pediatric populations^40–42^. Upon examining the AAPCs for kidney and renal pelvis cancers from 2000 to 2019, it was noted that there was a consistent upward trend across all age groups for both men and women. Notably, those aged < 39 years and the 40–54 age group demonstrated the largest increases. Educational attainment, race, and childhood socioeconomic status impact immune aging in older US adults. Lower education, Hispanic ethnicity, and lower parental education correlate with more immune aging^43^. Additionally, the US's aging population faces challenges due to disparities in economic status, aspirations, and access to resources within a wealthy society^44^. As the elderly population grows, there is a need to address these multifaceted factors to ensure the well-being and independence of older adults in the US^45^. By understanding and addressing these diverse factors, targeted interventions can be developed to improve the health and overall quality of life for the elderly population in the US.

In terms of sex, overall, the ASIRs for both kidney and renal pelvis cancers from 2000 to 2019 were generally higher in men. Previous literature has also reported congruent results regarding the sex patterns of incidence trends; a study by Dong et al. on the SEER data from 2001 to 2016 reported that the incidence of kidney and renal pelvis cancers was consistently higher in males across all age groups, except for the age group 0–14 years^46^. Schafer et al. observed a similar sex-related pattern in kidney, renal pelvis, and bladder cancers in the US^11^. According to their study, the incidence rates were higher in men than in women, with male-to-female ratios of 4.04 and 1.98 for kidney and renal pelvis cancer and bladder cancer, respectively^11^. The differences in cancer incidence and survival rates between sexes can be attributed to a combination of behavioral and environmental factors, along with biological distinctions^47^. Additionally, variations in immune system functions^48^, genetic factors, and hormonal differences^49^ are likely to play a pivotal role in these observed disparities between sexes. Cigarette smoking, contributing to around half of the cases in men and 40% in women in the US^50^, might partially elucidate the sex-based disparity in incidence.

### Incidence trends by race/ethnicity

Between 2000 and 2019, kidney and renal pelvis cancers showed distinctive patterns across ethnicities in the US. The majority of cases were among NHW individuals and NHB men had the highest ASIR of 24.53 per 100,000. In this regard, Schafer et al.'s research indicated an elevated occurrence of kidney and renal pelvis cancers in Black men compared to White or Hispanic individuals^11^. Notably, the study highlighted that the highest incidence rate across all races was found among American Indian and Alaska Native individuals^11^. Furthermore, the research noted that the highest incidence rate for bladder cancer was observed in white participants^11^. Additionally, the data of the Centers for Disease Control and Prevention of the US in 2014 showed Black men exhibited the highest likelihood of developing kidney and renal pelvis cancers at a rate of 24.7 per 100,000, followed by White men with rate of 22.0 per 100,000^51^. The observed variations in cancer outcomes have been linked to several contributing factors. Several studies pointed to racial disparities in healthcare access and treatment availability as one of the key elements^53–58^. Additionally, the quality of care^54,55,57^, patients' attitudes toward and beliefs in treatment decisions^54,55,57^, and the presence of comorbid conditions, along with the impact of stressful life events associated with socioeconomic status, have all been identified as influencing factors in this inequality. In the 2015–2019 period, NHW individuals still constituted the majority of kidney and renal pelvis cancers cases and NHB men had the highest reported ASIR. Remarkable variations emerged in Hispanic men, displaying a substantial rise in ASIR between 2015 and 2019, recording an AAPC of 3.19%. Kidney cancer was considered the fourth most common cancer in Hispanic men in 2019^58^. The escalation in incidence rates of kidney cancer among the Hispanic population has been also documented in prior scholarly literature^59^. Some studies have shown a nearly three-fold increase in the incidence of renal RCC among Hispanics^60,61^. This rise may be linked to disparities in disease presentation, with Hispanics showing higher rates of metastatic disease at diagnosis and more advanced stages of RCC compared to non-Hispanic/Latino individuals^62,63^. Additionally, differences in access to care, including insurance coverage and country of birth, have been noted, impacting the age of diagnosis, treatment choices, and outcomes among Hispanic RCC patients^64^.

### COVID-19 and incidence of kidney and renal pelvis cancers

The essential role of cancer screening in cancer identification has been significantly impeded by the disruption caused by COVID-19 to the cancer screening infrastructure. The disruption in cancer screening protocols contributes to a diminished reported incidence rate^65^. The impact of the COVID-19 era on kidney and renal pelvis cancers is evident in a substantial decline in the ASIR. From 2019 to November 2020, there was a noteworthy reduction in ASIR across all races/ethnicities, sexes, and age groups, with an overall 9.52% decrease. This decline was observed consistently in both males (− 8.49%) and females (− 11.31%). A study by Mariotto et al. on the SEER 2000–2020 data also reported that kidney and renal pelvis cancers had an incidence rate change of − 8.7% and − 11.5% in men and women, respectively, in 2020 compared with 2019^66^. The findings underscore the profound influence of the COVID-19 pandemic on the incidence trends of kidney and renal pelvis cancers, revealing a significant downturn during this specific period.

### Strengths and limitations

Our strength is reporting the incidence trends of kidney and renal pelvis cancers by histological subtypes using a high-quality population-based data, enabling a thorough examination of disparities and trends in the US. Also, case definition, statistical methods, and reporting estimates were according to SEER guidelines. The current study also has certain constraints. Firstly, SEER 22 database covers about half of the US population and our study does not encompass the incidence rates, APCs, and AAPCs for kidney and renal pelvis cancers across whole the population of the US and different geographical regions. Secondly, there is a possibility of slight misclassification in terms of patients' race or ethnicity. This misclassification could be due to the fact that healthcare practitioners carried out the initial data collection. Furthermore, the findings of our study, which were based on the US population, may not be applicable or generalizable to other Western countries. Moreover, when examining cancer rates across broad racial/ethnic categories, like Hispanics, there is a potential for overlooking significant variations based on the country of origin. Additionally, the inability to consider certain well-established risk factors, such as smoking and obesity, should be noted, as these data are not accessible in the SEER database. Also, consideration should be given to the statistical and clinical relevance of findings when p-values are greater than 0.05. This approach could offer greater insight into trends that might lack statistical significance but could still be clinically relevant.

Future studies could investigate the possible link between trends of kidney and pelvic cancers and their risk factors in the US. Furthermore, in future years, the SEER database should cover a larger population in the US and allow a more extensive examination into the influence of the COVID-19 pandemic on the identification of kidney and pelvic cancers.

## Conclusions

Overall, there is an observable upward trend in the incidence of kidney and renal pelvis cancer. Between 2000 and 2019, ASIRs of kidney cancer were nearly double in men compared to women. A notable increase in ASIR for both kidney and renal pelvis cancers in men occurred from 2015 to 2019. RCC emerged as the most common subtype, particularly impacting men, with the highest ASIR observed in individuals 70–84 years old. Regarding age, individuals aged 70–84 consistently had the highest ASIR for kidney cancer, while those 85 years or older had the highest ASIR for renal pelvis cancers. Racial variations highlighted that NHB men exhibited the highest incidence rates for both kidney and renal pelvis cancers. The impact of the COVID-19 era was evident in a substantial reduction in incidence rates across all demographics. To address these trends, it is imperative to implement targeted public health strategies and measures. In addition to establishing genetical screening programs for early detection, health education and awareness campaigns can empower individuals to adopt healthier lifestyles and seek timely medical care. Ensuring equitable access to healthcare services among all racial groups and promoting research initiatives to better understand the underlying causes of kidney cancer are critical steps in addressing disparities in cancer outcomes. Environmental policies aimed at reducing exposure to carcinogens can also play a role in mitigating the risk of developing kidney cancer. Furthermore, providing support services for individuals diagnosed with kidney and renal pelvis cancers and their families can improve overall quality of life and facilitate access to resources for managing the disease effectively. By adopting a comprehensive approach that integrates preventive measures and supportive services, public health efforts can effectively address the growing burden of kidney and renal pelvis cancers and improve outcomes for affected individuals.

### Supplementary Information

Supplementary Information 1.

Supplementary Information 2.

## Data Availability

The data used in this study are available from the Surveillance, Epidemiology, and End Results Program (SEER) database.
