# Supplementary material for: A population-based study on incidence trends of kidney and renal pelvis cancers in the United States over 2000–2020
Source: Sci Rep. 2024 May 17;14:11294. doi: 10.1038/s41598-024-61748-2 (PMC11101446; doi:10.1038/s41598-024-61748-2)
Supplement: Supplementary file 1 — Supplementary Information 1. [file 41598_2024_61748_MOESM1_ESM.docx]

**Appendix 1. Results**

**Renal cell carcinoma (RCC)**

**Men**

A total of 265,367 cases of RCC were reported in men during the period of 2000 to 2019. The majority of them were among NHWs (70.12%) and in individuals aged 55 to 69 years (43.31%). Individuals in the 70 to 84 age group had the highest ASIR (81.49 [80.90, 82.08]). All age groups showed a significant increase in ASIR from 2000 to 2019, with cases <39 years old having the highest AAPC (4.57%; [4.18, 5.07]) (Table 4).

Out of all the reported cases, 38,496 (14.51%) were among Hispanic individuals. The majority of cases were between 55 and 69 (40.80%). The overall ASIR per 100,000 population was 19.53 (19.32, 19.74). Cases between 70 and 84 years had the highest ASIR among all age groups (88.43 [86.51, 90.38]). Unlike those >85 which remained unchanged, all other age groups showed a significant increase in ASIR throughout 2000 to 2019 with cases <39 having the highest AAPC (6.81% [5.28, 8.79]) (Table 4).

NHBs accounted for 10.65% of the cases of RCC in men. The majority of NHB cases occurred in individuals aged 55 to 69 years (46.58%). The overall ASIR per 100,000 population for NHBs was 21.16 (20.90, 21.42), with cases between 70 and 84 years having the highest ASIR (86.11 [83.89, 88.36]). The overall AAPC for NHB over 2000-2019 was 2.41% (2.04, 2.96). unlike individuals >85, which remained unchanged, all other age groups had a significant rise in incidence rate moreover, those in the 40 to 54 age group had the highest AAPC of 2.81% (2.15, 3.55) (Table 4).

The majority of NHW cases occurred in individuals aged 55 to 69 years (43.39%). The overall ASIR per 100,000 population for NHW men was 19.12 (19.03, 19.21), with cases between 70 and 84 years having the highest ASIR (83.23 [82.54, 83.93]). There was a significant increase in the overall ASIR over the period of 2000 to 2019 in NHW men (AAPC: 2.26% [2.10, 2.50]). All individuals of all ages experienced a significant increase in ASIR from 2000-2019, with those <39 age group having the largest AAPC (4.34% [3.87, 4.86]) (Table 4).

**Women**

Between 2000 and 2019, a total of 153,159 cases of RCC were reported in women. The majority of these cases were among NHWs (67.15%) and between 55 and 69 years (39.32%). Individuals between 70 and 84 years had the highest ASIR among all age groups (38.92 [38.57, 39.28]). All cases in all age groups exhibited a significant increase, with cases <39 years having the highest rise in ASIR over 2000 to 2019 (4.63% [3.90, 5.68]) (Table 4).

Among all reported cases, 25,491 (16.64%) were among Hispanic individuals. The majority of cases were individuals between 55 and 69 years old (38.67%). The overall ASIR per 100,000 population was 10.98 (10.84, 11.11). Individuals between 70 and 84 years had the highest ASIR compared to other age groups (45.48 [44.31, 46.66]). The overall AAPC for Hispanic women was 2.43% (2.08, 2.89). All of the age groups showed a significant increase in ASIR over 2000-2019 with cases <39 having the highest rise compared to the other groups (5.77% [4.62, 7.33]) (Table 4).

NHBs constituted 11.75% of the cases of RCC in women. The major portion of NHB cases fell within the 55 to 69-year age group (42.46%). The overall ASIR per 100,000 population for NHB women was 10.44 (10.29, 10.60), with cases between 70 and 84 years old having the highest ASIR (44.23 [42.97, 45.51]). NHB women showed a significant increase in ASIR over 2000-2019 (2.42% [1.75, 3.38]) and cases between 55 to 69 years had the highest AAPC (3.03% [2.54, 3.74]) (Table 4).

The majority of NHW cases were in the 55 to 69 (38.91%) age group. The overall ASIR per 100,000 population was 9.26 (9.20, 9.32), with cases between 70 and 84 years old having the highest ASIR (38.90 [38.48, 39.32]). Over 2000-2019, there was a significant increase in the overall ASIR for NHW women, with an AAPC of 2.18% (2.02, 2.40) and individuals <39 years had the greatest increase compared to other age groups (4.49% [3.56, 5.69]) (Table 4).

**Nephroblastoma**

**Men**

A total of 2,142 cases of nephroblastoma were reported in men between 2000 and 2019. The majority of these cases were among NHWs (51.59%), and the largest portion of them occurred in individuals aged 0 to 4 years (73.44%). Among men with nephroblastoma, those aged <4 years had the highest ASIR (1.57 [1.49, 1.64]). Between 2000 and 2019, there was a notable rise in the incidence rate for cases aged between 5 and 9 years (AAPC: 2.04% [0.47, 3.79]) and a decrease for cases over 15 years (AAPC: -4.51% [-8.17, -1.24]) (Table 5).

Of the reported cases, 558 (26.05%) were among Hispanic individuals. The majority of cases were below 4 years (73.84%). The overall ASIR per 100,000 population for this group was 0.12 (0.11, 0.13). Cases <4 years had the highest ASIR among all age groups (1.24 [1.12, 1.37]). There were no significant changes in ASIRs in any of the age groups (Table 5).

NHBs accounted for 16.85% of the cases of nephroblastoma in men. A majority of NHB cases occurred in individuals under 4 years (72.58%). The overall ASIR per 100,000 population for NHBs was 0.19 (0.17, 0.21), and cases below 4 years had the highest ASIR among all age groups (2.00 [1.77, 2.26]). Individuals in the 5-9 age group exhibited a remarkable rise in the ASIR over 2000-2019 (AAPC: 4.58% [0.62, 9.92]) (Table 5).

The majority of NHWs were under 4 years (73.12%). The overall ASIR per 100,000 population for NHW men was 0.17 (0.16, 0.18), with cases below 4 years having the highest ASIR (1.76 [1.64, 1.89]). There was not significant changes in ASIRs over 2000-2019 (Table 5).

**Women**

Between 2000 and 2019, a total of 2,503 cases of nephroblastoma were reported in women. The majority of these cases were among NHWs (50.62%), and most of them were under 4 year (68.52%). NHBs had the highest ASIR per 100,000 population (0.24 [0.22, 0.26]) (Table 5).

Hispanics constituted 26.85% of the cases of nephroblastoma in women, with the major proportion in the 0 to 4-year age group (74.85%). The overall ASIR per 100,000 population for Hispanic women was 0.15 (0.14, 0.16), with cases <4 years having the highest ASIR among other age groups (1.58 [1.44, 1.72]). There were no significant changes in ASIRs over the period of 2000-2019 (Table 5).

Among all reported cases, 17.30% were NHBs. The majority of cases were <4 years (60.97%). The overall ASIR per 100,000 population was 0.24 (0.22, 0.26) with cases between 0 and 4 years having the highest ASIR [2.09 (1.84, 2.36]). There were no significant variations in ASIRs over 2000-2019 (Table 5).

The majority of NHW cases were in the 0 to 4 age group (66.93%). The overall ASIR per 100,000 population was 0.20 (0.19, 0.21), with cases under 4 years old having the highest ASIR (1.95 [1.82, 2.08]). Over 2000-2019, there were no significant changes in the overall ASIRs for NHW women (Table 5).

**Sarcoma**

**Men**

A total of 359 cases of sarcoma were reported in men over 2000-2019. The majority of the cases were NHWs (63.79%) and under 39 years (47.91%). Cases over 85 years had the highest ASIR per 100,000 population (0.07 [0.03, 0.12]). Between 2000 and 2019, cases between 55 and 69 years had the most substantial decline in ASIR with an AAPC of -6.65% (-9.10, -2.64) (Table 6).

Of all the reported cases, 74 (20.61%) were Hispanics. The majority of cases were under 39 (64.86%). The overall ASIR per 100,000 population was 0.02 (0.02, 0.03). Cases between 70 and 84 years had the highest ASIR among other age groups (0.05 [0.02, 0.12]) (Table 6).

NHBs consisted of 10.31% of the men with sarcoma. The majority of NHBs were below 39 years old (70.27%). The overall ASIR per 100,000 population was 0.02 (0.01, 0.03) and the cases between 55 to 69 years had the highest ASIR among other age groups (0.04 [0.02, 0.08]) (Table 6).

There were 229 reported cases of NHWs. The majority of the cases were under 39 years (37.99%). The overall ASIR per 100,000 population was 0.03 (0.02, 0.03), with cases over 85 years having the highest ASIR (0.09 [0.04, 0.16]). There was a significant decrease in individuals between 55 and 69 years over 2000-2019 (AAPC: -5.17% [-8.94, -1.67]) (Table 6).

**Women**

Between 2000 and 2019, a total of 207 cases of sarcoma were reported in women. The majority of these cases were among NHWs (61.35%) and those below 39 years (44.93%) (Table 6).

Among all reported cases, 44 (21.26%) were among Hispanic individuals. The majority of reported cases were under 39 years old (61.36%). The overall ASIR per 100,000 population for this group was 0.01 (0.01, 0.02). Cases among individuals between 55 and 69 years old had the highest ASIR compared to other age groups (0.03 [0.01, 0.06]) (Table 6).

NHBs constituted 9.18% of the cases of sarcoma in women. A significant portion of NHB cases fell below 39-years age group (47.37%). The overall ASIR per 100,000 population for NHB women was 0.01 (0.01, 0.02) (Table 6).

The majority of NHW cases were blow 39 years (37.01%). The overall ASIR per 100,000 population for this group was 0.01 (0.01, 0.02), with cases over 85 years having the highest ASIR (0.04 [0.02, 0.08]). Over the 2000-2019, there was a significant decline in ASIR for NHW women with an AAPC of -3.70% (-8.03, -0.19) (Table 6).

**Neuroendocrine tumor**

**Men**

A total of 164 cases of neuroendocrine tumor were reported in men between 2000 and 2019. The majority of these cases were among NHWs (77.44%) and those aged 70 to 84 years (37.80%). Those over 85 years old had the highest ASIR (0.08 [0.04, 0.14]) (Table 7).

Out of all the reported cases, 7.93% were among Hispanic individuals. The majority of cases were between 55 and 84 years (61.54%). The overall ASIR per 100,000 population for this group was 0.01 (0.00, 0.01). Cases between 70 and 84 years had the highest ASIR among all age groups (0.05 [0.01, 0.12]). There were no changes in AAPC among age groups (Table 7).

NHBs accounted for 9.15% of the cases of neuroendocrine tumor. The majority of NHB cases occurred between 55 and 84 years (66.67%). The overall ASIR per 100,000 population was 0.01 (0.01, 0.02). There were no changes in AAPC among age groups (Table 7).

The majority of NHWs were in individuals aged 70 to 84 years (40.16%). The overall ASIR per 100,000 population for NHW men was 0.01 (0.01, 0.02). There were no significant changes in AAPCs (Table 7).

**Women**

Between 2000 and 2019, a total of 136 cases of neuroendocrine tumors were reported in women. The majority of these cases were among NHWs (70.59%) and between 55 and 69 years (29.41%) (Table 7).

Among all reported cases, 9.56% were among Hispanic individuals. The majority of cases were individuals between 70 and 84 years old (30.77%). The overall ASIR per 100,000 population was 0.01 (0.00, 0.01). Cases over 85 years had the highest ASIR per 100,000 population compared to other age groups (0.08 [0.01, 0.28]). There were no changes in the ASIRs among any of the age groups over 2000-2019 (Table 7).

NHBs constituted 13.24% of the cases of neuroendocrine tumors in women. A significant portion of NHB cases were among 55 to 69-year age group (38.88%). The overall ASIR per 100,000 population for NHB women was 0.01 (0.01, 0.02), with cases over 85 years having the highest ASIR among other age groups (0.04 [0.00, 0.23]). There were no change in ASIRs among any of the age groups (Table 7).

The majority of NHW cases were in the 70 to 84 age group (33.33%). The overall ASIR per 100,000 population was 0.01 (0.01, 0.01), with cases between 70 and 84 years having the highest ASIR (0.04 [0.02, 0.05]). From 2000-2019, there were no significant change in ASIRs (Table 7).
