# Supplementary material for: A population-based study on incidence trends of kidney and renal pelvis cancers in the United States over 2000–2020
Source: Sci Rep. 2024 May 17;14:11294. doi: 10.1038/s41598-024-61748-2 (PMC11101446; doi:10.1038/s41598-024-61748-2)
Supplement: Supplementary file 2 — Supplementary Information 2. [file 41598_2024_61748_MOESM2_ESM.docx]

**Table S1.** Counts and age-standardized rate of kidney and renal pelvis cancers incidence per 100,000 and average annual percent change from 2015 to 2019 in the United States, by age, sex, and race.

| **All race/ethnicities** | | | | | | |
| --- | --- | --- | --- | --- | --- | --- |
| **Age group (years)** | Men | | | Women | | |
|  | **Case (%)** | **ASIR (95% CI)** | **AAPC (95% CI)** | **Case (%)** | **ASIR (95% CI)** | **AAPC (95% CI)** |
| All | 97605(63.48) | 24.08 (23.93, 24.24) | 1.29 (1.09, 1.8) | 56153(36.52) | 12.15 (12.05, 12.26) | 1.35 (0.93, 2.34) |
| 0 to 39 | 4266 (2.77) | 2.13 (2.06, 2.19) | 1.32 (0.31, 2.04) | 3306 (2.15) | 1.67 (1.61, 1.72) | 1.39 (-0.51, 2.41) |
| 40 to 54 | 18310 (11.91) | 24.04 (23.69, 24.39) | 2.16 (0.98, 2.47) | 9763 (6.35) | 12.59 (12.34, 12.84) | 2.13 (-1.07, 3.09) |
| 55 to 69 | 42821 (27.85) | 67.6 (66.96, 68.24) | 1.36 (1.01, 2.11) | 22287 (14.49) | 32.18 (31.76, 32.61) | 1.38 (1.02, 2.19) |
| 70 to 84 | 27974 (18.19) | 106.26 (105, 107.52) | 1.04 (0.72, 1.81) | 16868 (10.97) | 51.07 (50.3, 51.85) | 1.56 (-0.03, 2.75) |
| +85 | 4234 (2.75) | 84.23 (81.71, 86.81) | -0.02 (-0.78, 1.55) | 3929 (2.56) | 42.2 (40.89, 43.54) | -0.69 (-1.47, -0.13) |
| Hispanic | | | | | | |
| Age groups | Men | | | Women | | |
|  | Case (%) | ASIR (95% CI) | AAPC (95% CI) | Case (%) | ASIR (95% CI) | AAPC (95% CI) |
| All | 16228 (60.01) | 24.72 (24.31, 25.13) | 2.16 (1.63, 3.35) | 10812 (39.99) | 14.21 (13.94, 14.49) | 2 (1.65, 2.46) |
| 0 to 39 | 1189 (4.40) | 2.03 (1.92, 2.15) | 4.24 (3.62, 5) | 1042 (3.85) | 1.86 (1.74, 1.97) | 4.45 (3.33, 5.87) |
| 40 to 54 | 4294 (15.88) | 23.66 (22.96, 24.38) | 2.55 (2.25, 2.9) | 2754 (10.18) | 15.35 (14.78, 15.93) | 3.19 (2.62, 3.89) |
| 55 to 69 | 6750 (24.96) | 69.67 (68, 71.36) | 1.54 (0.03, 2.01) | 4192 (15.50) | 39.36 (38.17, 40.57) | 2.08 (1.63, 2.65) |
| 70 to 84 | 3501 (12.95) | 111.53 (107.83, 115.33) | 2.33 (1.5, 4.01) | 2388 (8.83) | 56.68 (54.42, 59) | 0.89 (0.25, 1.65) |
| +85 | 494 (1.85) | 91.56 (83.66, 100) | 0.08 (-1, 1.52) | 436 (1.61) | 45.44 (41.28, 49.91) | 0.87 (-0.02, 2.04) |
| NHB | | | | | | |
| Age groups | Men | | | Women | | |
|  | Case (%) | ASIR (95% CI) | AAPC (95% CI) | Case (%) | ASIR (95% CI) | AAPC (95% CI) |
| All | 10698 (60.76) | 26.98 (26.44, 27.52) | 1.16 (0.61, 1.56) | 6910 (39.24) | 13.66 (13.33, 13.99) | 0.62 (-0.33, 1.28) |
| 0 to 39 | 574 (3.26) | 2.37 (2.18, 2.58) | 2.46 (1.34, 3.7) | 427 (2.43) | 1.66 (1.51, 1.83) | 1.54 (0.48, 2.7) |
| 40 to 54 | 2406 (13.66) | 27.94 (26.82, 29.09) | 2.56 (1.9, 3.3) | 1229 (6.98) | 12.74 (12.03, 13.48) | 1.74 (0.97, 2.58) |
| 55 to 69 | 5150 (29.25) | 81.41 (79.2, 83.68) | 0.53 (-0.59, 1.2) | 3085 (17.52) | 39.65 (38.26, 41.08) | 2.27 (1.35, 3.93) |
| 70 to 84 | 2336 (13.27) | 111.46 (106.93, 116.13) | 0.8 (-0.21, 1.35) | 1842 (10.46) | 57.96 (55.34, 60.68) | 0.06 (-3.06, 1.28) |
| +85 | 232 (1.32) | 73.69 (64.51, 83.8) | -0.37 (-2.24, 1.91) | 327 (1.86) | 44.46 (39.77, 49.55) | -3.73 (-11.23, -0.46) |
| NHW | | | | | | |
| Age groups | Men | | | Women | | |
|  | Case (%) | ASIR (95% CI) | AAPC (95% CI) | Case (%) | ASIR (95% CI) | AAPC (95% CI) |
| All | 65267 (64.83) | 24.77 (24.57, 24.97) | 1.26 (1.02, 1.88) | 35408 (35.17) | 12.11 (11.98, 12.25) | 1.19 (0.61, 2.05) |
| 0 to 39 | 2166 (2.15) | 2.26 (2.16, 2.35) | 0.57 (-1.62, 1.9) | 1620 (1.61) | 1.74 (1.66, 1.83) | 0.49 (-2, 1.86) |
| 40 to 54 | 10408 (10.34) | 24.9 (24.42, 25.39) | 2.14 (-0.16, 2.54) | 5194 (5.16) | 12.53 (12.19, 12.89) | 1.81 (0.74, 2.28) |
| 55 to 69 | 28594 (28.40) | 68.23 (67.44, 69.03) | 1.31 (0.63, 2.53) | 13808 (13.72) | 31.08 (30.56, 31.6) | 0.9 (0.36, 1.41) |
| 70 to 84 | 20778 (20.64) | 109.99 (108.49, 111.51) | 0.97 (0.66, 1.77) | 11801 (11.72) | 51.65 (50.72, 52.59) | 0.5 (-0.2, 1.9) |
| +85 | 3321 (3.30) | 88.45 (85.47, 91.52) | 0.17 (-0.38, 1.51) | 2985 (2.96) | 43.21 (41.67, 44.78) | -0.33 (-1.6, 0.29) |

Abbreviations: NHW: Non-Hispanic White; NHB: Non-Hispanic Black; ASIR: Age-standardized incidence rate; CI: Confidence interval, AAPC: Average annual percent change.

**Table S2.** Results of the tests of incidental for kidney and renal pelvis cancers incidence rate over 2000-2019 in the United States

| **Race / ethnicities** | **Sex** | **Subtype** | **Race / ethnicities** | **Sex** | **Subtype** | **P value** |
| --- | --- | --- | --- | --- | --- | --- |
| **Cohort 1** | | | **Cohort 2** | | |  |
| All | Female | Neuroendocrine tumor | NHW | Female | Neuroendocrine tumor | 0.09 |
| All | Female | RCC | NHW | Female | RCC | 0.08 |
| All | Male | Sarcoma | Hispanic | Male | Sarcoma | 0.91 |
| All | Male | Sarcoma | NHW | Male | Sarcoma | 0.07 |
| All | Both | Sarcoma | Hispanic | Both | Sarcoma | 0.51 |
| All | Both | Sarcoma | NHB | Both | Sarcoma | 0.13 |
| All | Female | Neuroendocrine tumor | All | Both | Neuroendocrine tumor | 0.06 |
| Hispanic | Male | RCC | NHW | Male | RCC | 0.43 |
| Hispanic | Female | renal pelvic | NHB | Female | renal pelvis | 0.1 |
| Hispanic | Male | Sarcoma | NHW | Male | Sarcoma | 0.67 |
| Hispanic | Both | Sarcoma | NHW | Both | Sarcoma | 0.24 |
| NHB | Male | Nephroblastoma | NHW | Both | Nephroblastoma | 0.1 |
| NHB | Both | Sarcoma | NHW | Both | Sarcoma | 0.06 |
| NHB | Female | Nephroblastoma | NHB | Male | Nephroblastoma | 0.08 |
| NHB | Female | Nephroblastoma | NHB | Both | Nephroblastoma | 0.18 |
| NHB | Male | Nephroblastoma | NHB | Both | Nephroblastoma | 0.17 |
| NHW | Female | Neuroendocrine tumor | NHW | Both | Neuroendocrine tumor | 0.07 |

Abbreviations: NHW: Non-Hispanic White; NHB: Non-Hispanic Black; RCC: renal cell carcinoma.

**Table S3**. Results of the tests of parallelism for kidney and pelvic cancers incidence rate over 2000-2019 in the United States.

| **Race/ ethnicities** | **sex** | **subtypes** | **Race/ ethnicities** | **sex** | **subtype** | **P value** |
| --- | --- | --- | --- | --- | --- | --- |
| **Cohort 1** | | | **Cohort 2** | | |  |
| All | Male | Kidney cancer | NHW | Male | Kidney cancer | 0.71 |
| All | Male | kidney and renal pelvis | NHW | Male | kidney and renal pelvis | 0.7 |
| All | Both | kidney and renal pelvis | NHW | Both | kidney and renal pelvis | 0.13 |
| All | Female | Nephroblastoma | Hispanic | Female | Nephroblastoma | 0.19 |
| All | Female | Nephroblastoma | NHB | Female | Nephroblastoma | 0.8 |
| All | Female | Nephroblastoma | NHW | Female | Nephroblastoma | 0.89 |
| All | Male | Nephroblastoma | Hispanic | Male | Nephroblastoma | 0.51 |
| All | Male | Nephroblastoma | NHB | Male | Nephroblastoma | 0.47 |
| All | Male | Nephroblastoma | NHW | Male | Nephroblastoma | 0.44 |
| All | Both | Nephroblastoma | Hispanic | Both | Nephroblastoma | 0.07 |
| All | Both | Nephroblastoma | NHB | Both | Nephroblastoma | 0.64 |
| All | Both | Nephroblastoma | NHW | Both | Nephroblastoma | 0.79 |
| All | Female | Neuroendocrine tumor | NHW | Female | Neuroendocrine tumor | 0.07 |
| All | Female | RCC | NHW | Female | RCC | 0.06 |
| All | Male | RCC | Hispanic | Male | RCC | 0.14 |
| All | Male | RCC | NHB | Male | RCC | 0.51 |
| All | Male | RCC | NHW | Male | RCC | 0.85 |
| All | Both | RCC | NHB | Both | RCC | 0.08 |
| All | Both | RCC | NHW | Both | RCC | 0.19 |
| All | Female | renal pelvis | Hispanic | Female | renal pelvis | 0.85 |
| All | Female | renal pelvis | NHB | Female | renal pelvis | 0.78 |
| All | Female | renal pelvis | NHW | Female | renal pelvis | 0.26 |
| All | Male | renal pelvis | Hispanic | Male | renal pelvis | 0.33 |
| All | Male | renal pelvis | NHB | Male | renal pelvis | 0.82 |
| All | Both | renal pelvis | Hispanic | Both | renal pelvis | 0.31 |
| All | Both | renal pelvis | NHB | Both | renal pelvis | 0.96 |
| All | Male | Sarcoma | Hispanic | Male | Sarcoma | 0.87 |
| All | Male | Sarcoma | NHW | Male | Sarcoma | 0.87 |
| All | Both | Sarcoma | Hispanic | Both | Sarcoma | 0.44 |
| All | Both | Sarcoma | NHB | Both | Sarcoma | 0.07 |
| All | Both | Sarcoma | NHW | Both | Sarcoma | 0.06 |
| All | Female | Kidney cancer | All | Male | Kidney cancer | 0.13 |
| All | Female | Kidney cancer | All | Both | Kidney cancer | 0.08 |
| All | Male | Kidney cancer | All | Both | Kidney cancer | 0.13 |
| All | Female | kidney and renal pelvis | All | Male | kidney and renal pelvis | 0.3 |
| All | Female | kidney and renal pelvis | All | Both | kidney and renal pelvis | 0.14 |
| All | Male | kidney and renal pelvis | All | Both | kidney and renal pelvis | 0.32 |
| All | Female | Nephroblastoma | All | Male | Nephroblastoma | 0.66 |
| All | Female | Nephroblastoma | All | Both | Nephroblastoma | 0.56 |
| All | Male | Nephroblastoma | All | Both | Nephroblastoma | 0.59 |
| All | Female | Neuroendocrine tumor | All | Male | Neuroendocrine tumor | 0.7 |
| All | Female | Neuroendocrine tumor | All | Both | Neuroendocrine tumor | 0.53 |
| All | Male | Neuroendocrine tumor | All | Both | Neuroendocrine tumor | 0.85 |
| All | Female | RCC | All | Male | RCC | 0.3 |
| All | Female | RCC | All | Both | RCC | 0.08 |
| All | Male | RCC | All | Both | RCC | 0.14 |
| All | Female | renal pelvis | All | Male | renal pelvis | 0.12 |
| All | Female | renal pelvis | All | Both | renal pelvis | 0.34 |
| All | Female | Sarcoma | All | Male | Sarcoma | 0.62 |
| All | Female | Sarcoma | All | Both | Sarcoma | 0.59 |
| All | Male | Sarcoma | All | Both | Sarcoma | 0.4 |
| Hispanic | Male | Kidney cancer | NHB | Male | Kidney cancer | 0.06 |
| Hispanic | Male | Kidney cancer | NHW | Male | Kidney cancer | 0.05 |
| Hispanic | Female | Nephroblastoma | NHB | Female | Nephroblastoma | 0.74 |
| Hispanic | Female | Nephroblastoma | NHW | Female | Nephroblastoma | 0.68 |
| Hispanic | Male | Nephroblastoma | NHB | Male | Nephroblastoma | 0.7 |
| Hispanic | Male | Nephroblastoma | NHW | Male | Nephroblastoma | 0.98 |
| Hispanic | Both | Nephroblastoma | NHB | Both | Nephroblastoma | 0.18 |
| Hispanic | Both | Nephroblastoma | NHW | Both | Nephroblastoma | 0.25 |
| Hispanic | Male | RCC | NHB | Male | RCC | 0.64 |
| Hispanic | Male | RCC | NHW | Male | RCC | 0.35 |
| Hispanic | Both | RCC | NHB | Both | RCC | 0.16 |
| Hispanic | Female | renal pelvis | NHB | Female | renal pelvis | 0.83 |
| Hispanic | Female | renal pelvis | NHW | Female | renal pelvis | 0.67 |
| Hispanic | Male | renal pelvis | NHB | Male | renal pelvis | 0.79 |
| Hispanic | Male | renal pelvis | NHW | Male | renal pelvis | 0.38 |
| Hispanic | Both | renal pelvis | NHB | Both | renal pelvis | 0.66 |
| Hispanic | Both | renal pelvis | NHW | Both | renal pelvis | 0.26 |
| Hispanic | Male | Sarcoma | NHW | Male | Sarcoma | 0.75 |
| Hispanic | Both | Sarcoma | NHW | Both | Sarcoma | 0.1 |
| Hispanic | Female | Kidney cancer | Hispanic | Male | Kidney cancer | 0.18 |
| Hispanic | Female | Kidney cancer | Hispanic | Both | Kidney cancer | 0.79 |
| Hispanic | Male | Kidney cancer | Hispanic | Both | Kidney cancer | 0.33 |
| Hispanic | Female | kidney and renal pelvis | Hispanic | Male | kidney and renal pelvis | 0.46 |
| Hispanic | Female | kidney and renal pelvis | Hispanic | Both | kidney and renal pelvis | 0.66 |
| Hispanic | Male | kidney and renal pelvis | Hispanic | Both | kidney and renal pelvis | 0.34 |
| Hispanic | Female | Nephroblastoma | Hispanic | Male | Nephroblastoma | 0.89 |
| Hispanic | Female | Nephroblastoma | Hispanic | Both | Nephroblastoma | 0.84 |
| Hispanic | Male | Nephroblastoma | Hispanic | Both | Nephroblastoma | 0.83 |
| Hispanic | Female | RCC | Hispanic | Male | RCC | 0.53 |
| Hispanic | Female | RCC | Hispanic | Both | RCC | 0.78 |
| Hispanic | Male | RCC | Hispanic | Both | RCC | 0.39 |
| Hispanic | Female | renal pelvis | Hispanic | Male | renal pelvis | 0.45 |
| Hispanic | Female | renal pelvis | Hispanic | Both | renal pelvis | 0.45 |
| Hispanic | Male | renal pelvis | Hispanic | Both | renal pelvis | 0.38 |
| Hispanic | Male | Sarcoma | Hispanic | Both | Sarcoma | 0.54 |
| NHB | Female | Nephroblastoma | NHW | Female | Nephroblastoma | 0.8 |
| NHB | Male | Nephroblastoma | NHW | Male | Nephroblastoma | 0.74 |
| NHB | Both | Nephroblastoma | NHW | Both | Nephroblastoma | 0.84 |
| NHB | Male | RCC | NHW | Male | RCC | 0.63 |
| NHB | Female | renal pelvis | NHW | Female | renal pelvis | 0.92 |
| NHB | Male | renal pelvis | NHW | Male | renal pelvis | 0.83 |
| NHB | Both | renal pelvis | NHW | Both | renal pelvis | 0.97 |
| NHB | Female | Kidney cancer | NHB | Male | Kidney cancer | 0.19 |
| NHB | Female | Kidney cancer | NHB | Both | Kidney cancer | 0.26 |
| NHB | Male | Kidney cancer | NHB | Both | Kidney cancer | 0.29 |
| NHB | Female | kidney and renal pelvis | NHB | Male | kidney and renal pelvis | 0.08 |
| NHB | Female | kidney and renal pelvis | NHB | Both | kidney and renal pelvis | 0.29 |
| NHB | Female | Nephroblastoma | NHB | Male | Nephroblastoma | 0.62 |
| NHB | Female | Nephroblastoma | NHB | Both | Nephroblastoma | 0.81 |
| NHB | Male | Nephroblastoma | NHB | Both | Nephroblastoma | 0.57 |
| NHB | Female | RCC | NHB | Male | RCC | 0.34 |
| NHB | Female | RCC | NHB | Both | RCC | 0.3 |
| NHB | Female | renal pelvis | NHB | Male | renal pelvis | 0.52 |
| NHB | Female | renal pelvis | NHB | Both | renal pelvis | 0.53 |
| NHB | Male | renal pelvis | NHB | Both | renal pelvis | 0.42 |
| NHW | Male | Kidney cancer | NHW | Both | Kidney cancer | 0.08 |
| NHW | Male | kidney and renal pelvis | NHW | Both | kidney and renal pelvis | 0.18 |
| NHW | Female | Nephroblastoma | NHW | Male | Nephroblastoma | 0.99 |
| NHW | Female | Nephroblastoma | NHW | Both | Nephroblastoma | 0.97 |
| NHW | Male | Nephroblastoma | NHW | Both | Nephroblastoma | 0.94 |
| NHW | Female | Neuroendocrine tumor | NHW | Male | Neuroendocrine tumor | 0.35 |
| NHW | Female | Neuroendocrine tumor | NHW | Both | Neuroendocrine tumor | 0.15 |
| NHW | Male | Neuroendocrine tumor | NHW | Both | Neuroendocrine tumor | 0.68 |
| NHW | Female | RCC | NHW | Male | RCC | 0.12 |
| NHW | Male | RCC | NHW | Both | RCC | 0.25 |
| NHW | Female | renal pelvis | NHW | Male | renal pelvis | 0.45 |
| NHW | Female | renal pelvis | NHW | Both | renal pelvis | 0.64 |
| NHW | Male | renal pelvis | NHW | Both | renal pelvis | 0.07 |
| NHW | Female | Sarcoma | NHW | Male | Sarcoma | 0.46 |
| NHW | Female | Sarcoma | NHW | Both | Sarcoma | 0.45 |
| NHW | Male | Sarcoma | NHW | Both | Sarcoma | 0.34 |
| all races | Female | Kidney cancer | all races | Female | kidney and renal pelvis | 0.06 |
| all races | Female | Kidney cancer | all races | Female | Nephroblastoma | 0.06 |
| all races | Female | Kidney cancer | all races | Female | Neuroendocrine tumor | 0.46 |
| all races | Female | Kidney cancer | all races | Female | Sarcoma | 0.09 |
| all races | Female | kidney and renal pelvis | all races | Female | Nephroblastoma | 0.09 |
| all races | Female | kidney and renal pelvis | all races | Female | Neuroendocrine tumor | 0.5 |
| all races | Female | kidney and renal pelvis | all races | Female | Sarcoma | 0.1 |
| all races | Female | Nephroblastoma | all races | Female | Neuroendocrine tumor | 0.15 |
| all races | Female | Nephroblastoma | all races | Female | renal pelvis | 0.61 |
| all races | Female | Nephroblastoma | all races | Female | Sarcoma | 0.07 |
| all races | Female | Neuroendocrine tumor | all races | Female | RCC | 0.32 |
| all races | Female | Neuroendocrine tumor | all races | Female | renal pelvis | 0.3 |
| all races | Female | Neuroendocrine tumor | all races | Female | Sarcoma | 0.1 |
| all races | Female | renal pelvis | all races | Female | Sarcoma | 0.14 |
| all races | Male | Kidney cancer | all races | Male | Neuroendocrine tumor | 0.28 |
| all races | Male | kidney and renal pelvis | all races | Male | Nephroblastoma | 0.06 |
| all races | Male | kidney and renal pelvis | all races | Male | Neuroendocrine tumor | 0.38 |
| all races | Male | Nephroblastoma | all races | Male | Neuroendocrine tumor | 0.61 |
| all races | Male | Nephroblastoma | all races | Male | renal pelvis | 0.51 |
| all races | Male | Nephroblastoma | all races | Male | Sarcoma | 0.29 |
| all races | Male | Neuroendocrine tumor | all races | Male | RCC | 0.14 |
| all races | Male | Neuroendocrine tumor | all races | Male | renal pelvis | 0.87 |
| all races | Male | Neuroendocrine tumor | all races | Male | Sarcoma | 0.4 |
| all races | Both | Kidney cancer | all races | Both | Neuroendocrine tumor | 0.86 |
| all races | Both | kidney and renal pelvis | all races | Both | Neuroendocrine tumor | 0.85 |
| all races | Both | Nephroblastoma | all races | Both | Neuroendocrine tumor | 0.36 |
| all races | Both | Nephroblastoma | all races | Both | renal pelvis | 0.31 |
| all races | Both | Nephroblastoma | all races | Both | Sarcoma | 0.05 |
| all races | Both | Neuroendocrine tumor | all races | Both | RCC | 0.9 |
| all races | Both | Neuroendocrine tumor | all races | Both | renal pelvis | 0.54 |
| all races | Both | Neuroendocrine tumor | all races | Both | Sarcoma | 0.11 |
| Hispanic | Female | Kidney cancer | Hispanic | Female | kidney and renal pelvis | 0.06 |
| Hispanic | Female | Kidney cancer | Hispanic | Female | Nephroblastoma | 0.06 |
| Hispanic | Female | kidney and renal pelvis | Hispanic | Female | Nephroblastoma | 0.05 |
| Hispanic | Female | Nephroblastoma | Hispanic | Female | renal pelvis | 0.44 |
| Hispanic | Male | Kidney cancer | Hispanic | Male | Sarcoma | 0.37 |
| Hispanic | Male | kidney and renal pelvis | Hispanic | Male | Nephroblastoma | 0.13 |
| Hispanic | Male | kidney and renal pelvis | Hispanic | Male | Sarcoma | 0.39 |
| Hispanic | Male | Nephroblastoma | Hispanic | Male | RCC | 0.07 |
| Hispanic | Male | Nephroblastoma | Hispanic | Male | renal pelvis | 0.34 |
| Hispanic | Male | Nephroblastoma | Hispanic | Male | Sarcoma | 0.36 |
| Hispanic | Male | RCC | Hispanic | Male | Sarcoma | 0.19 |
| Hispanic | Male | renal pelvis | Hispanic | Male | Sarcoma | 0.58 |
| Hispanic | Both | kidney and renal pelvis | Hispanic | Both | Sarcoma | 0.07 |
| Hispanic | Both | Nephroblastoma | Hispanic | Both | renal pelvis | 0.06 |
| Hispanic | Both | renal pelvis | Hispanic | Both | Sarcoma | 0.43 |
| NHB | Female | Kidney cancer | NHB | Female | Nephroblastoma | 0.25 |
| NHB | Female | Kidney cancer | NHB | Female | renal pelvis | 0.17 |
| NHB | Female | kidney and renal pelvis | NHB | Female | Nephroblastoma | 0.24 |
| NHB | Female | kidney and renal pelvis | NHB | Female | renal pelvis | 0.1 |
| NHB | Female | Nephroblastoma | NHB | Female | RCC | 0.3 |
| NHB | Female | Nephroblastoma | NHB | Female | renal pelvis | 0.92 |
| NHB | Female | RCC | NHB | Female | renal pelvis | 0.31 |
| NHB | Male | Kidney cancer | NHB | Male | kidney and renal pelvis | 0.17 |
| NHB | Male | Kidney cancer | NHB | Male | Nephroblastoma | 0.22 |
| NHB | Male | kidney and renal pelvis | NHB | Male | Nephroblastoma | 0.13 |
| NHB | Male | Nephroblastoma | NHB | Male | RCC | 0.29 |
| NHB | Male | Nephroblastoma | NHB | Male | renal pelvis | 0.13 |
| NHB | Both | Kidney cancer | NHB | Both | Nephroblastoma | 0.15 |
| NHB | Both | Kidney cancer | NHB | Both | Sarcoma | 0.35 |
| NHB | Both | kidney and renal pelvis | NHB | Both | Nephroblastoma | 0.14 |
| NHB | Both | kidney and renal pelvis | NHB | Both | Sarcoma | 0.28 |
| NHB | Both | Nephroblastoma | NHB | Both | RCC | 0.23 |
| NHB | Both | Nephroblastoma | NHB | Both | renal pelvis | 0.45 |
| NHB | Both | Nephroblastoma | NHB | Both | Sarcoma | 0.26 |
| NHB | Both | RCC | NHB | Both | Sarcoma | 0.56 |
| NHB | Both | renal pelvis | NHB | Both | Sarcoma | 0.12 |
| NHW | Female | Kidney cancer | NHW | Female | kidney and renal pelvis | 0.06 |
| NHW | Female | Kidney cancer | NHW | Female | Nephroblastoma | 0.07 |
| NHW | Female | Kidney cancer | NHW | Female | Neuroendocrine tumor | 0.29 |
| NHW | Female | Kidney cancer | NHW | Female | Sarcoma | 0.12 |
| NHW | Female | kidney and renal pelvis | NHW | Female | Nephroblastoma | 0.13 |
| NHW | Female | kidney and renal pelvis | NHW | Female | Neuroendocrine tumor | 0.27 |
| NHW | Female | Nephroblastoma | NHW | Female | renal pelvis | 0.97 |
| NHW | Female | Neuroendocrine tumor | NHW | Female | RCC | 0.14 |
| NHW | Female | Neuroendocrine tumor | NHW | Female | renal pelvis | 0.09 |
| NHW | Female | Neuroendocrine tumor | NHW | Female | Sarcoma | 0.11 |
| NHW | Male | Kidney cancer | NHW | Male | Nephroblastoma | 0.17 |
| NHW | Male | Kidney cancer | NHW | Male | Neuroendocrine tumor | 0.72 |
| NHW | Male | Kidney cancer | NHW | Male | Sarcoma | 0.09 |
| NHW | Male | kidney and renal pelvis | NHW | Male | Nephroblastoma | 0.28 |
| NHW | Male | kidney and renal pelvis | NHW | Male | Neuroendocrine tumor | 0.61 |
| NHW | Male | Nephroblastoma | NHW | Male | Neuroendocrine tumor | 0.33 |
| NHW | Male | Nephroblastoma | NHW | Male | RCC | 0.1 |
| NHW | Male | Nephroblastoma | NHW | Male | renal pelvis | 0.72 |
| NHW | Male | Nephroblastoma | NHW | Male | Sarcoma | 0.21 |
| NHW | Male | Neuroendocrine tumor | NHW | Male | RCC | 0.84 |
| NHW | Male | Neuroendocrine tumor | NHW | Male | renal pelvis | 0.67 |
| NHW | Male | Neuroendocrine tumor | NHW | Male | Sarcoma | 0.32 |
| NHW | Male | RCC | NHW | Male | Sarcoma | 0.08 |
| NHW | Both | Kidney cancer | NHW | Both | Nephroblastoma | 0.09 |
| NHW | Both | Kidney cancer | NHW | Both | Neuroendocrine tumor | 0.56 |
| NHW | Both | kidney and renal pelvis | NHW | Both | Nephroblastoma | 0.1 |
| NHW | Both | kidney and renal pelvis | NHW | Both | Neuroendocrine tumor | 0.47 |
| NHW | Both | Nephroblastoma | NHW | Both | Neuroendocrine tumor | 0.19 |
| NHW | Both | Nephroblastoma | NHW | Both | renal pelvis | 0.54 |
| NHW | Both | Neuroendocrine tumor | NHW | Both | RCC | 0.47 |
| NHW | Both | Neuroendocrine tumor | NHW | Both | renal pelvis | 0.35 |

Abbreviations: NHW: Non-Hispanic White; NHB: Non-Hispanic Black; RCC: renal cell carcinoma.

**Table S4.** Counts and age-standardized rate of kidney cancer incidence per 100,000 and average annual percent change from 2015 to 2019 in the United States, by age, sex, and race.

| **All race/ethnicities** | | | | | | |
| --- | --- | --- | --- | --- | --- | --- |
| **Age group (years)** | Men | | | Women | | |
|  | **Case (%)** | **ASIR (95% CI)** | **AAPC (95% CI)** | **Case (%)** | **ASIR (95% CI)** | **AAPC (95% CI)** |
| **All** | 93226 (63.77) | 22.66 (22.51, 22.81) | 1.2 (0.94, 1.82) | 52958 (36.23) | 11.35 (11.26, 11.45) | 0.76 (0.44, 1.03) |
| **0 to 39** | 4248 (2.91) | 2.1 (2.03, 2.16) | 1.18 (0.15, 1.94) | 3290 (2.25) | 1.64 (1.59, 1.7) | 1.24 (-0.4, 2.22) |
| **40 to 54** | 18034 (12.34) | 23.44 (23.1, 23.79) | 2.04 (1.29, 2.39) | 9610 (6.57) | 12.27 (12.03, 12.52) | 2.06 (-0.55, 2.53) |
| **55 to 69** | 41403 (28.32) | 64.62 (64, 65.25) | 1.24 (0.82, 2.01) | 21505 (14.71) | 30.71 (30.3, 31.12) | 0.8 (0.54, 1.02) |
| **70 to 84** | 25847 (17.68) | 96.67 (95.49, 97.87) | 0.94 (0.67, 1.49) | 15204 (10.40) | 45.39 (44.67, 46.12) | 1.34 (0.03, 2.35) |
| **+85** | 3694 (2.53) | 72.58 (70.26, 74.96) | -0.85 (-1.62, -0.31) | 3349 (2.29) | 35.52 (34.33, 36.75) | -1.08 (-2.05, -0.41) |
| **Hispanic** | | | | | | |
| **Age groups** | **Men** | | | **Women** | | |
|  | **Case (%)** | **ASIR (95% CI)** | **AAPC (95% CI)** | **Case (%)** | **ASIR (95% CI)** | **AAPC (95% CI)** |
| **All** | 15763 (60.01) | 23.45 (23.06, 23.85) | 1.31 (0.75, 1.63) | 10505 (39.33) | 13.53 (13.27, 13.8) | 1.66 (-0.63, 2.34) |
| **0 to 39** | 1184 (4.51) | 2 (1.88, 2.12) | 4.24 (3.61, 5) | 1034 (3.94) | 1.82 (1.71, 1.93) | 0.64 (-5.39, 4.41) |
| **40 to 54** | 4237 (16.13) | 23.02 (22.33, 23.72) | 2.47 (2.17, 2.82) | 2732 (10.40) | 15.01 (14.46, 15.59) | 3.12 (2.56, 3.81) |
| **55 to 69** | 6592 (25.10) | 67.02 (65.4, 68.67) | 1.46 (0.07, 1.93) | 4109 (15.64) | 38 (36.85, 39.19) | 2.04 (1.56, 2.67) |
| **70 to 84** | 3302 (12.57) | 103.31 (99.78, 106.93) | 3 (1.38, 5.02) | 2238 (8.52) | 52.21 (50.06, 54.43) | 0.84 (0.24, 1.56) |
| **+85** | 448 (1.71) | 81.74 (74.35, 89.67) | -0.09 (-1.26, 1.48) | 392 (1.49) | 40.22 (36.33, 44.4) | 0.71 (-0.17, 1.87) |
| **NHB** | | | | | | |
| **Age groups** | **Men** | | | **Women** | | |
|  | **Case (%)** | **ASIR (95% CI)** | **AAPC (95% CI)** | **Case (%)** | **ASIR (95% CI)** | **AAPC (95% CI)** |
| **All** | 10499 (61.02) | 25.95 (25.43, 26.48) | 0.81 (0.15, 1.29) | 6717 (38.98) | 13.02 (12.7, 13.34) | 0.46 (-0.41, 1.1) |
| **0 to 39** | 572 (3.32) | 2.32 (2.13, 2.52) | 2.37 (1.23, 3.66) | 424 (2.46) | 1.62 (1.47, 1.78) | 1.45 (0.42, 2.58) |
| **40 to 54** | 2376 (13.80) | 27.13 (26.04, 28.25) | 2.48 (1.78, 3.28) | 1209 (7.02) | 12.32 (11.63, 13.04) | 1.69 (0.8, 2.66) |
| **55 to 69** | 5070 (29.45) | 78.75 (76.59, 80.96) | 0.35 (-0.71, 1.06) | 3025 (17.57) | 38.22 (36.87, 39.61) | 1.96 (0.64, 3.94) |
| **70 to 84** | 2258 (13.12) | 105.7 (101.33, 110.21) | 0.62 (-0.72, 1.26) | 1756 (10.20) | 54.21 (51.7, 56.82) | -0.16 (-4.03, 1.21) |
| **+85** | 223 (1.30) | 69.6 (60.77, 79.36) | -0.56 (-2.47, 1.77) | 303 (1.76) | 40.49 (36.06, 45.32) | -4.17 (-12.56, -0.71) |
| **NHW** | | | | | | |
| **Age groups** | **Men** | | | **Women** | | |
|  | **Case (%)** | **ASIR (95% CI)** | **AAPC (95% CI)** | **Case (%)** | **ASIR (95% CI)** | **AAPC (95% CI)** |
| **All** | 61825 (65.24) | 23.21 (23.02, 23.4) | 0.94 (0.73, 1.12) | 32935 (34.76) | 11.23 (11.11, 11.36) | 0.49 (0.14, 0.77) |
| **0 to 39** | 2156 (2.28) | 2.22 (2.13, 2.32) | 0.43 (-1.63, 1.74) | 1615 (1.70) | 1.72 (1.64, 1.81) | 0.31 (-2.12, 1.71) |
| **40 to 54** | 10236 (10.80) | 24.26 (23.78, 24.74) | 2.09 (0.89, 2.47) | 5099 (5.38) | 12.19 (11.85, 12.54) | 1.75 (0.91, 2.2) |
| **55 to 69** | 27511 (29.03) | 64.97 (64.2, 65.74) | 0.73 (-0.03, 1.07) | 13236 (13.97) | 29.5 (28.99, 30) | 0.8 (0.35, 1.64) |
| **70 to 84** | 19061 (20.12) | 99.5 (98.08, 100.93) | 0.92 (0.57, 1.77) | 10481 (11.06) | 45.31 (44.44, 46.19) | -0.22 (-0.7, 0.16) |
| **+85** | 2861 (3.02) | 75.35 (72.62, 78.16) | -0.64 (-1.74, -0.11) | 2504 (2.64) | 35.84 (34.45, 37.27) | -0.8 (-1.99, -0.14) |

Abbreviations: NHW: Non-Hispanic White; NHB: Non-Hispanic Black; ASIR: Age-standardized incidence rate; CI: Confidence interval, AAPC: Average annual percent change.

**Table S5.** Counts and age-standardized rate of renal pelvis cancer incidence per 100,000 and average annual percent change from 2015 to 2019 in the United States, by age, sex, and race.

| **All race/ethnicities** | | | | | | |
| --- | --- | --- | --- | --- | --- | --- |
| **Age group (years)** | Men | | | Women | | |
|  | **Case (%)** | **ASIR (95% CI)** | **AAPC (95% CI)** | **Case (%)** | **ASIR (95% CI)** | **AAPC (95% CI)** |
| **All** | 4379 (57.82) | 1.14 (1.11, 1.18) | -1.31 (-5.4, -0.49) | 3195 (42.18) | 0.66 (0.64, 0.68) | -0.12 (-0.55, 0.33) |
| **0 to 39** | 18 (0.24) | 0.01 (0.01, 0.01) | -2.94 (-7.15, 0.61) | 16 (0.21) | 0.01 (0, 0.01) | -0.72 (-4.46, 3.25) |
| **40 to 54** | 276 (3.64) | 0.35 (0.31, 0.4) | -2.19 (-3.33, -1.09) | 153 (2.02) | 0.19 (0.16, 0.22) | -0.71 (-1.96, 0.54) |
| **55 to 69** | 1418 (18.72) | 2.21 (2.1, 2.33) | -1.65 (-2.35, -0.9) | 782 (10.32) | 1.11 (1.03, 1.19) | -1.9 (-2.73, -1.01) |
| **70 to 84** | 2127 (28.08) | 8.22 (7.87, 8.58) | -1.12 (-4.27, -0.39) | 1664 (21.97) | 5.03 (4.79, 5.27) | 0.28 (-0.41, 1.02) |
| **+85** | 540 (7.13) | 10.61 (9.73, 11.54) | -0.44 (-4.23, 0.66) | 580 (7.66) | 6.15 (5.66, 6.67) | 1.47 (0.23, 2.91) |
| **Hispanic** | | | | | | |
| **Age groups** | **Men** | | | **Women** | | |
|  | **Case (%)** | **ASIR (95% CI)** | **AAPC (95% CI)** | **Case (%)** | **ASIR (95% CI)** | **AAPC (95% CI)** |
| **All** | 465 (60.23) | 0.9 (0.81, 0.99) | -0.86 (-2.3, 0.89) | 307 (39.77) | 0.47 (0.42, 0.53) | -0.24 (-1.67, 1.47) |
| **0 to 39** | 5 (0.65) | 0.01 (0, 0.02) | N/A | 8 (1.04) | 0.01 (0.01, 0.03) | N/A |
| **40 to 54** | 57 (7.38) | 0.31 (0.23, 0.4) | 1.83 (-0.68, 10.67) | 22 (2.85) | 0.12 (0.08, 0.18) | -0.89 (-5.21, 4.04) |
| **55 to 69** | 158 (20.47) | 1.61 (1.37, 1.89) | -1.64 (-3.99, 1.26) | 83 (10.75) | 0.78 (0.62, 0.96) | -1.17 (-3.15, 1.16) |
| **70 to 84** | 199 (25.78) | 6.49 (5.61, 7.47) | -1 (-3.14, 1.53) | 150 (19.43) | 3.57 (3.02, 4.19) | -0.14 (-1.67, 1.66) |
| **+85** | 46 (5.96) | 8.39 (6.14, 11.2) | N/A | 44 (5.70) | 4.51 (3.28, 6.06) | N/A |
| **NHB** | | | | | | |
| **Age groups** | **Men** | | | **Women** | | |
|  | **Case (%)** | **ASIR (95% CI)** | **AAPC (95% CI)** | **Case (%)** | **ASIR (95% CI)** | **AAPC (95% CI)** |
| **All** | 199 (50.77) | 0.57 (0.49, 0.66) | -0.66 (-2.16, 0.99) | 193 (49.23) | 0.4 (0.34, 0.46) | 0.03 (-1.9, 2.21) |
| **0 to 39** | 2 (0.51) | 0.01 (0, 0.03) | N/A | 3 (0.77) | 0.01 (0, 0.03) | N/A |
| **40 to 54** | 30 (7.65) | 0.34 (0.23, 0.49) | -0.75 (-3.85, 2.56) | 20 (5.10) | 0.2 (0.12, 0.31) | N/A |
| **55 to 69** | 80 (20.41) | 1.27 (1.01, 1.58) | -2.18 (-4.69, 0.56) | 60 (15.31) | 0.75 (0.57, 0.97) | -1.9 (-5.98, 3.05) |
| **70 to 84** | 78 (19.90) | 3.77 (2.98, 4.72) | 0.06 (-2.05, 2.52) | 86 (21.94) | 2.73 (2.18, 3.37) | 0.34 (-1.47, 2.41) |
| **+85** | 9 (2.30) | 2.81 (1.28, 5.33) | N/A | 24 (6.12) | 3.21 (2.06, 4.77) | N/A |
| **NHW** | | | | | | |
| **Age groups** | **Men** | | | **Women** | | |
|  | **Case (%)** | **ASIR (95% CI)** | **AAPC (95% CI)** | **Case (%)** | **ASIR (95% CI)** | **AAPC (95% CI)** |
| **All** | 3442 (58.19) | 1.29 (1.25, 1.34) | -1.11 (-6.8, 0.56) | 2473 (41.81) | 0.75 (0.72, 0.78) | 0.09 (-0.53, 0.75) |
| **0 to 39** | 10 (0.17) | 0.01 (0.01, 0.02) | N/A | 5 (0.08) | 0.01 (0, 0.01) | N/A |
| **40 to 54** | 172 (2.91) | 0.39 (0.34, 0.46) | -2.17 (-3.43, -1.07) | 95 (1.61) | 0.21 (0.17, 0.26) | -0.11 (-1.83, 1.57) |
| **55 to 69** | 1083 (18.31) | 2.53 (2.39, 2.69) | -1.45 (-2.19, -0.67) | 572 (9.67) | 1.25 (1.15, 1.36) | -2.08 (-2.97, -1.17) |
| **70 to 84** | 1717 (29.03) | 9.23 (8.8, 9.68) | -0.9 (-4.64, 0.04) | 1320 (22.31) | 5.75 (5.44, 6.07) | 0.65 (-0.1, 1.45) |
| **+85** | 460 (7.78) | 12.12 (11.03, 13.27) | 0.2 (-7.54, 2.81) | 481 (8.13) | 6.89 (6.28, 7.53) | 1.79 (0.7, 3.05) |

Abbreviations: NHW: Non-Hispanic White; NHB: Non-Hispanic Black; ASIR: Age-standardized Incidence rate; CI: Confidence interval, AAPC: Average annual percent change; N/A: Not available.


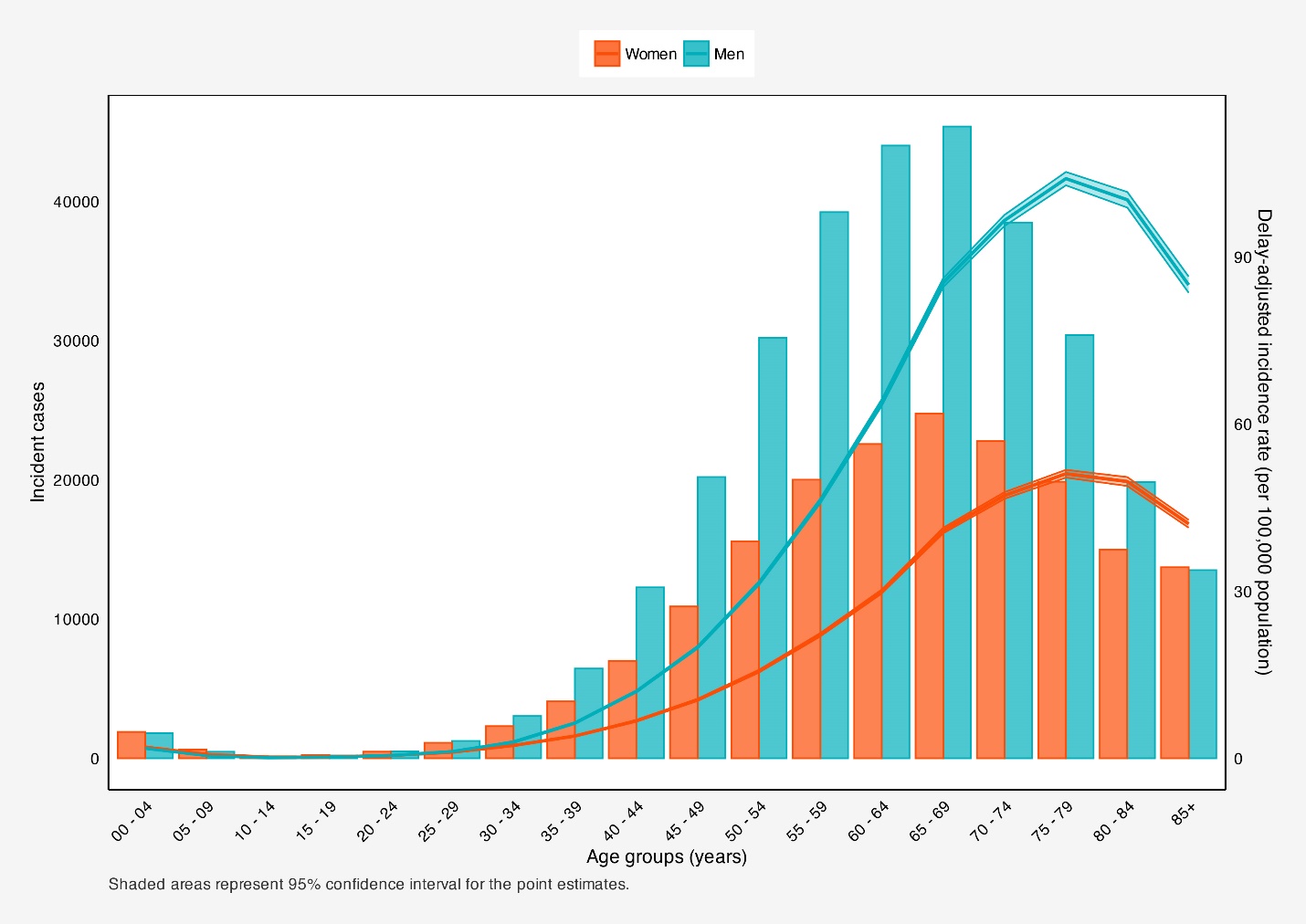
**Figure S1.** Incident cases and incidence rate of kidney and renal pelvis cancers in the United States among males and females in each age group. Shaded areas are the confidence interval range for the point estimates.


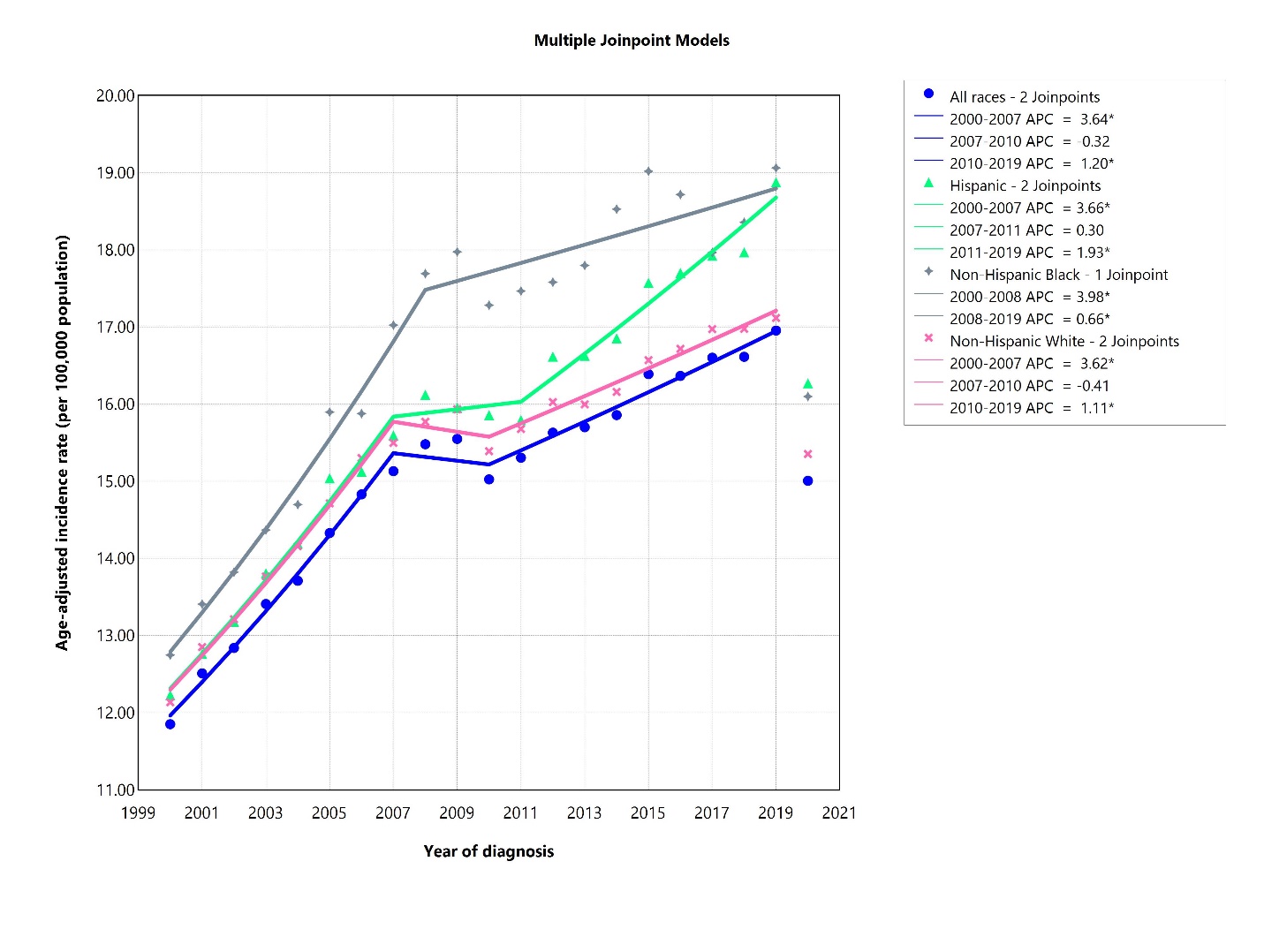
**Figure S2.** Age-adjusted incidence rate of kidney cancer over 2000-2019 and in 2020 in the United States, by race. APC: annual percent change. * Represent p-value less than 0.05.


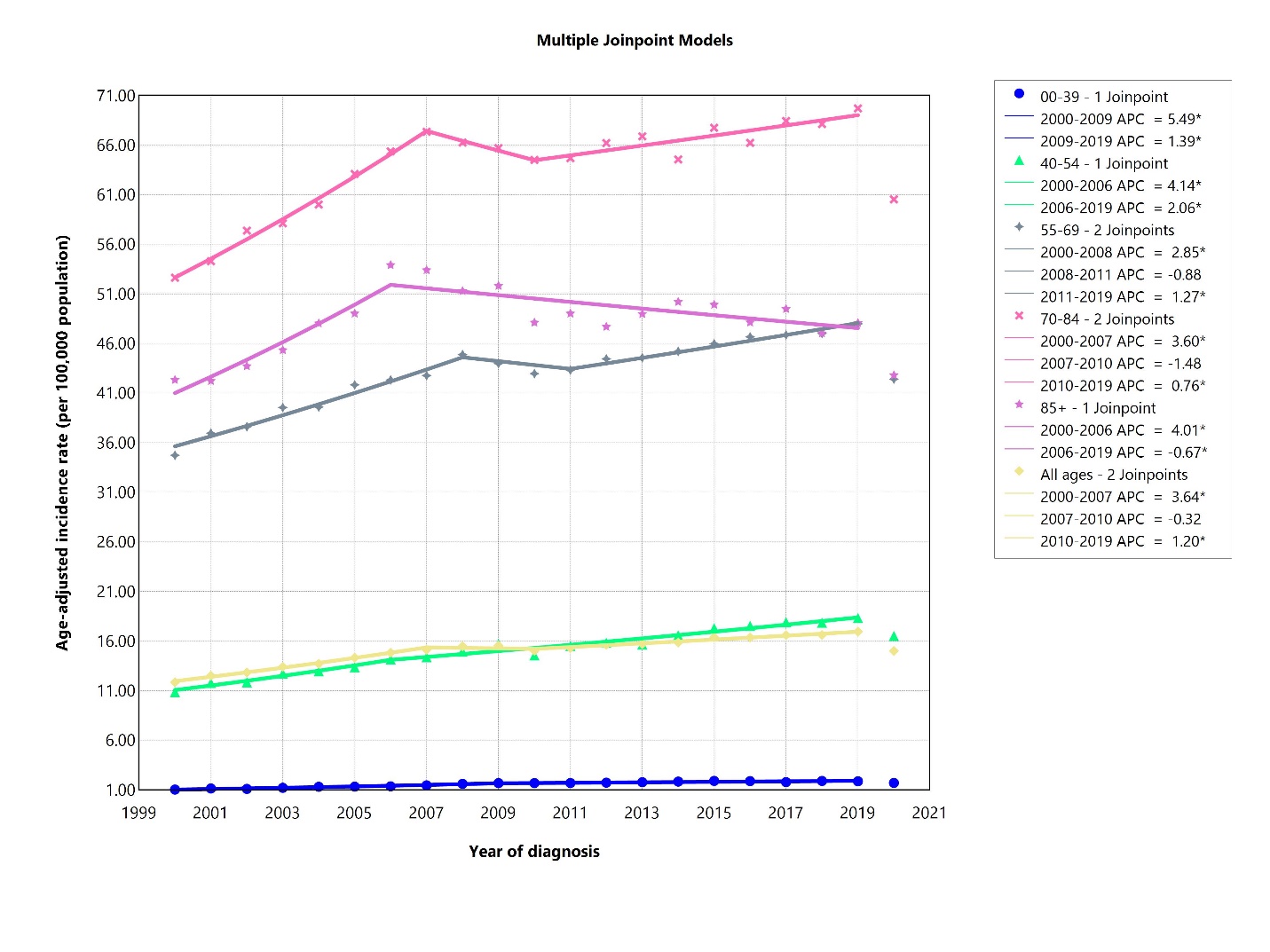
**Figure S3.** Age-adjusted incidence rate of kidney cancer over 2000-2019 and in 2020 in the United States, by age. APC: annual percent change. * Represent p-value less than 0.05.


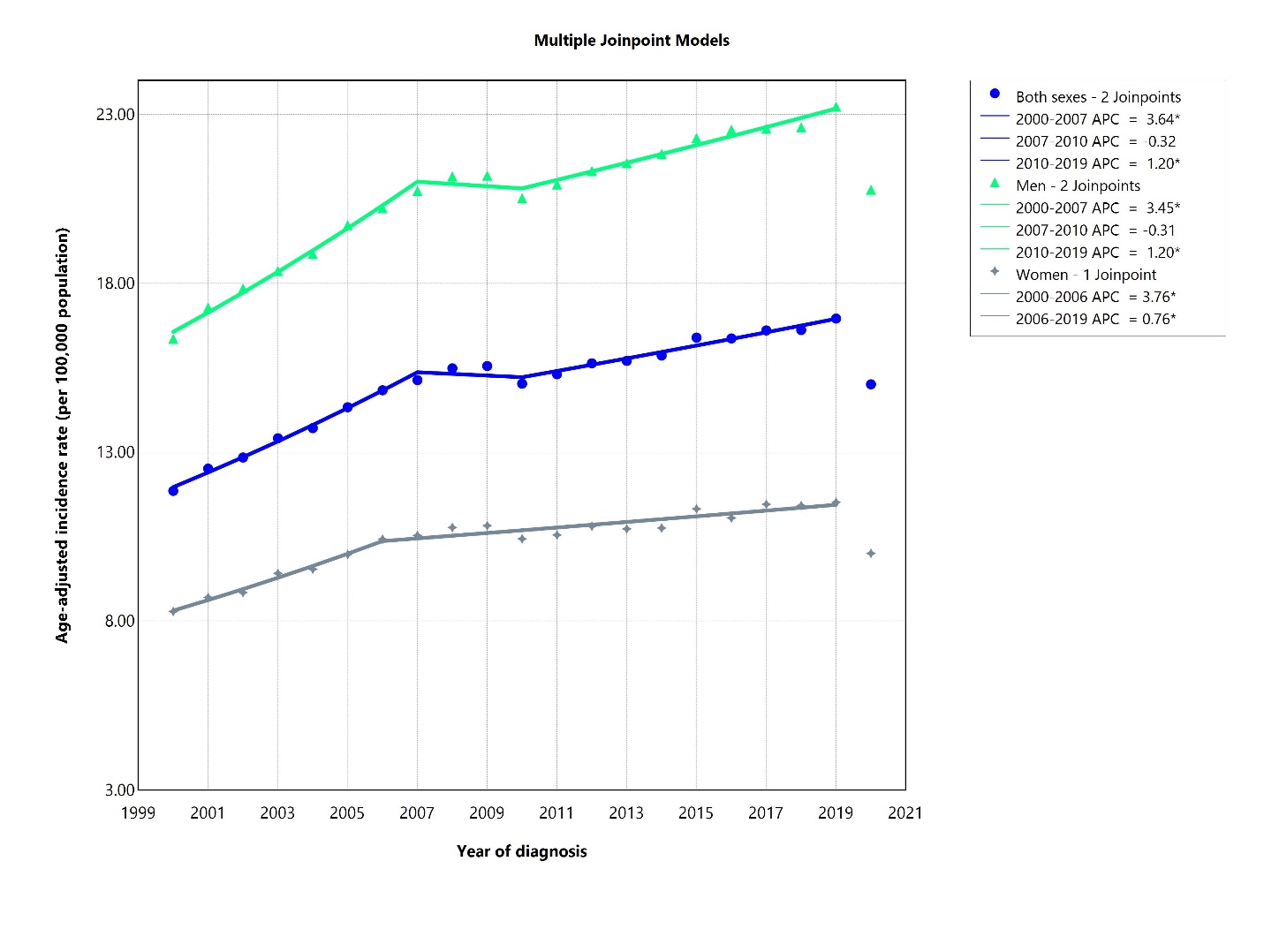
 **Figure S4.** Age-adjusted incidence rate of kidney cancer over 2000-2019 and in 2020 in the United States, by sex. APC: annual percent change. * Represent p-value less than 0.05.


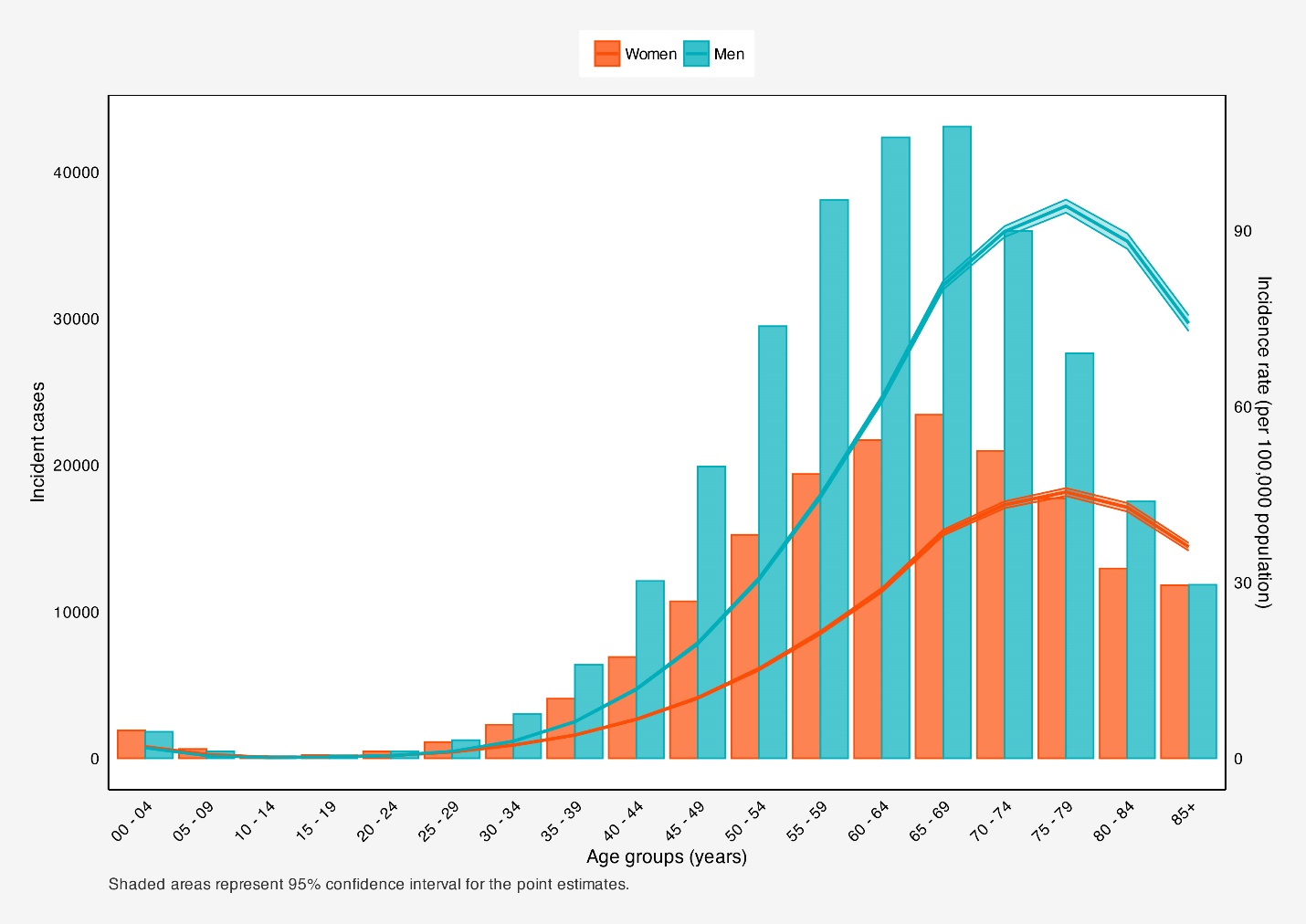


**Figure S5.** Incident cases and incidence rate of kidney cancer in the United States among males and females in each age group. Shaded areas are the confidence interval range for the point estimates.


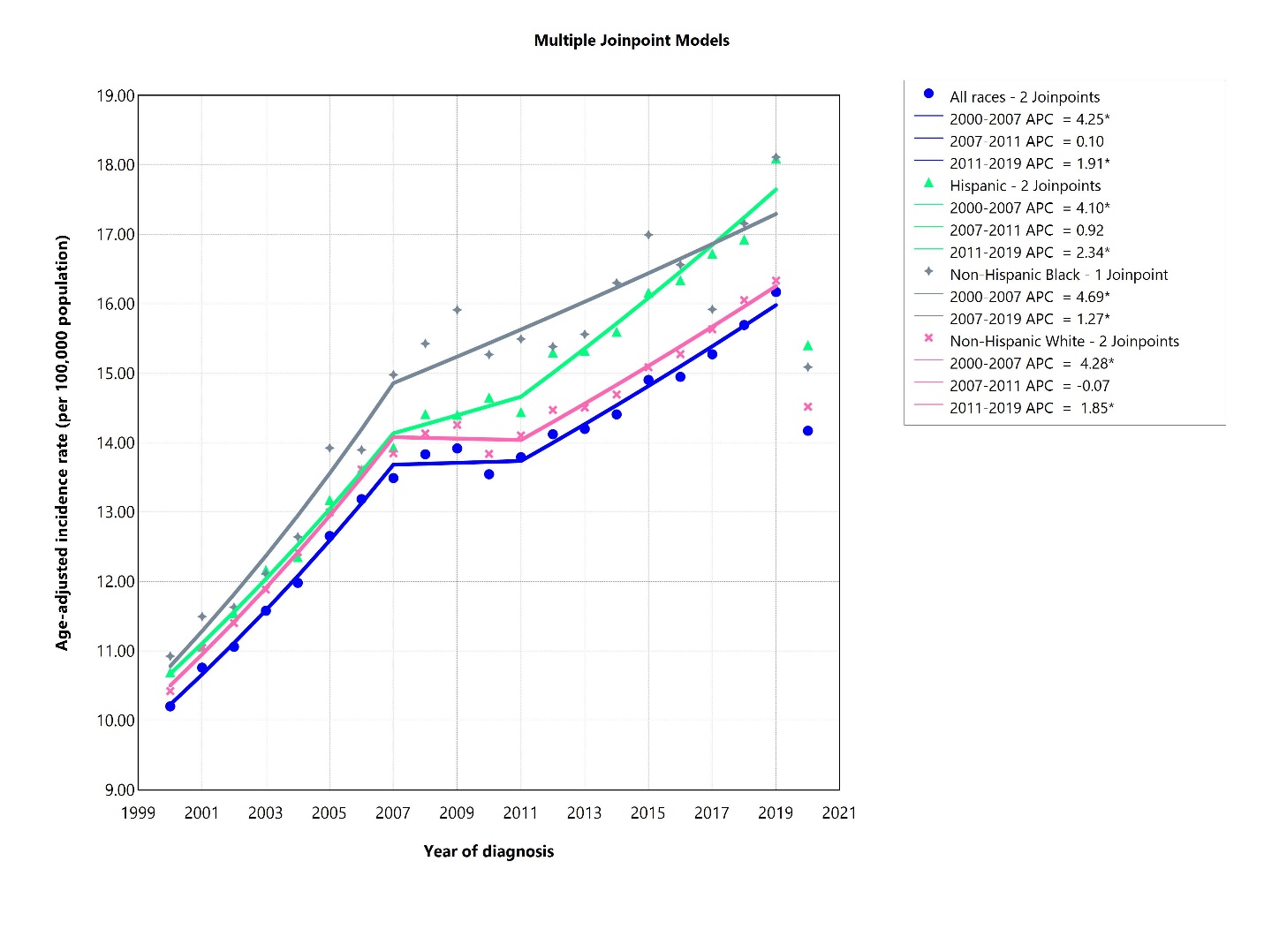
 **Figure S6.** Age-adjusted incidence rate of renal cell carcinoma over 2000-2019 and in 2020 in the United States, by race. APC: annual percent change. * Represent p-value less than 0.05.


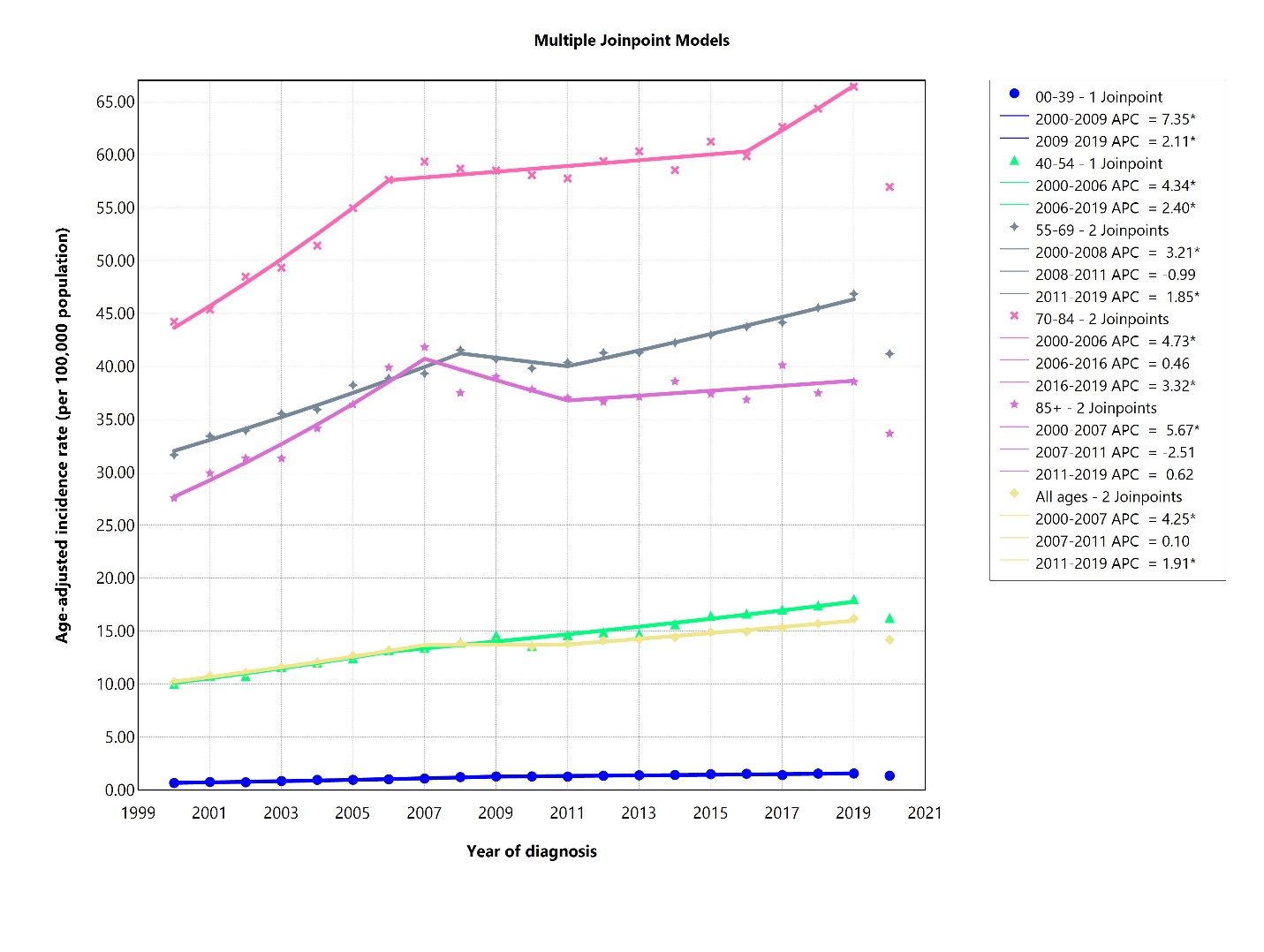


**Figure S7.** Age-adjusted incidence rate of renal cell carcinoma over 2000-2019 and in 2020 in the United States, by age. APC: annual percent change. * Represent p-value less than 0.05.


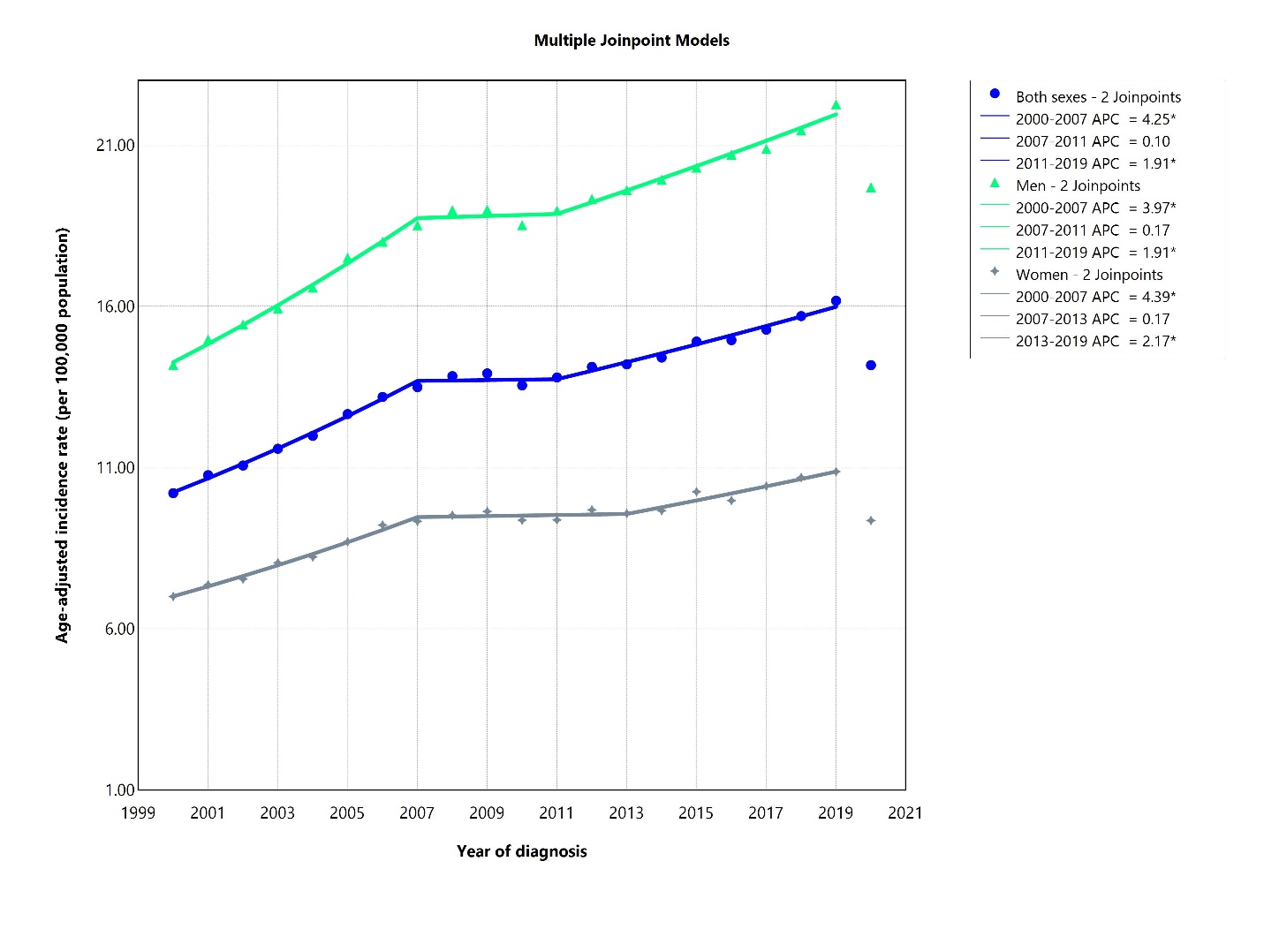
**Figure S8.** Age-adjusted incidence rate of renal cell carcinoma over 2000-2019 and in 2020 in the United States, by sex. APC: annual percent change. * Represent p-value less than 0.05.


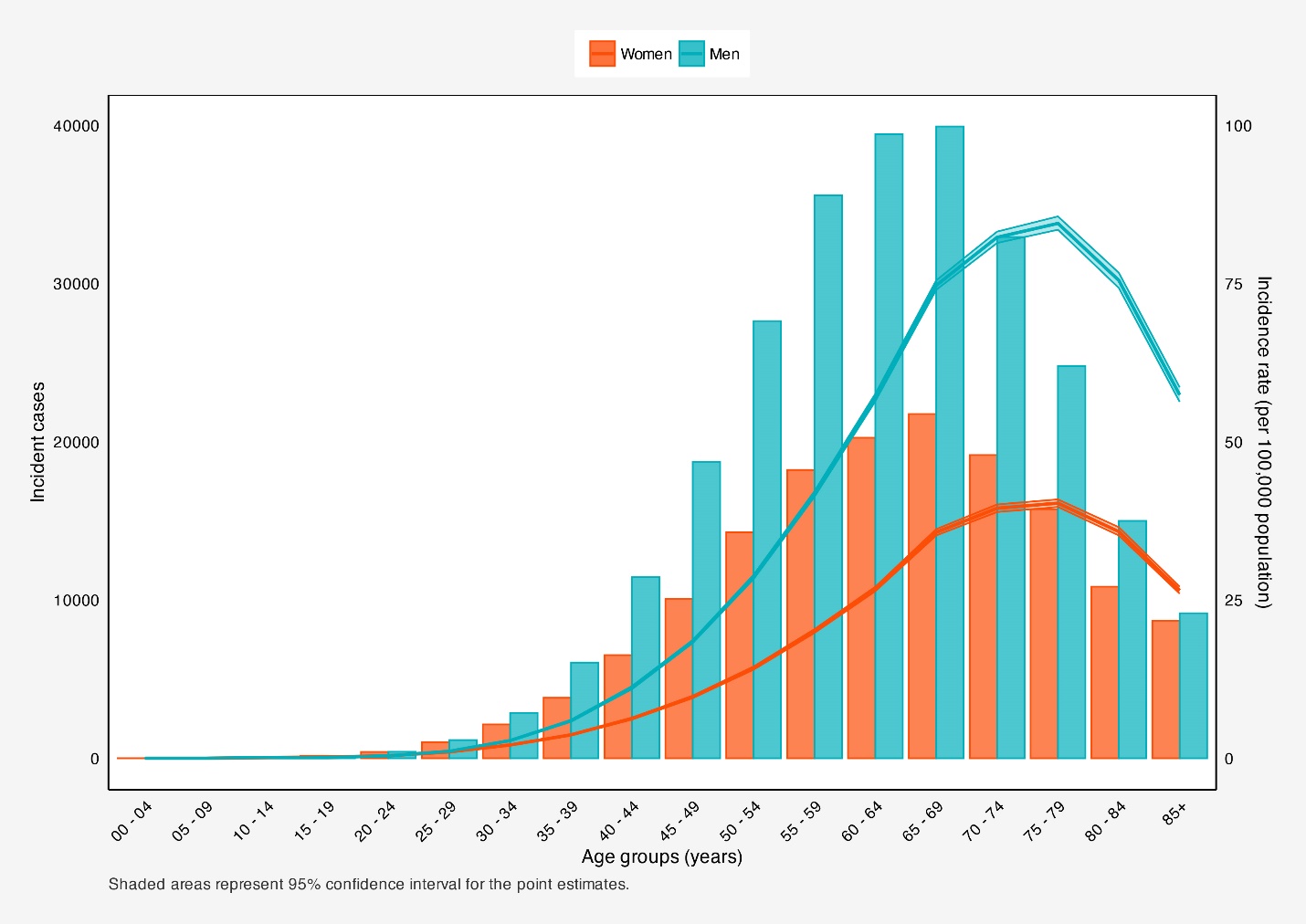


**Figure S9.** Incident cases and incidence rate of renal cell carcinoma in the United States among males and females in each age group. Shaded areas are the confidence interval range for the point estimates.


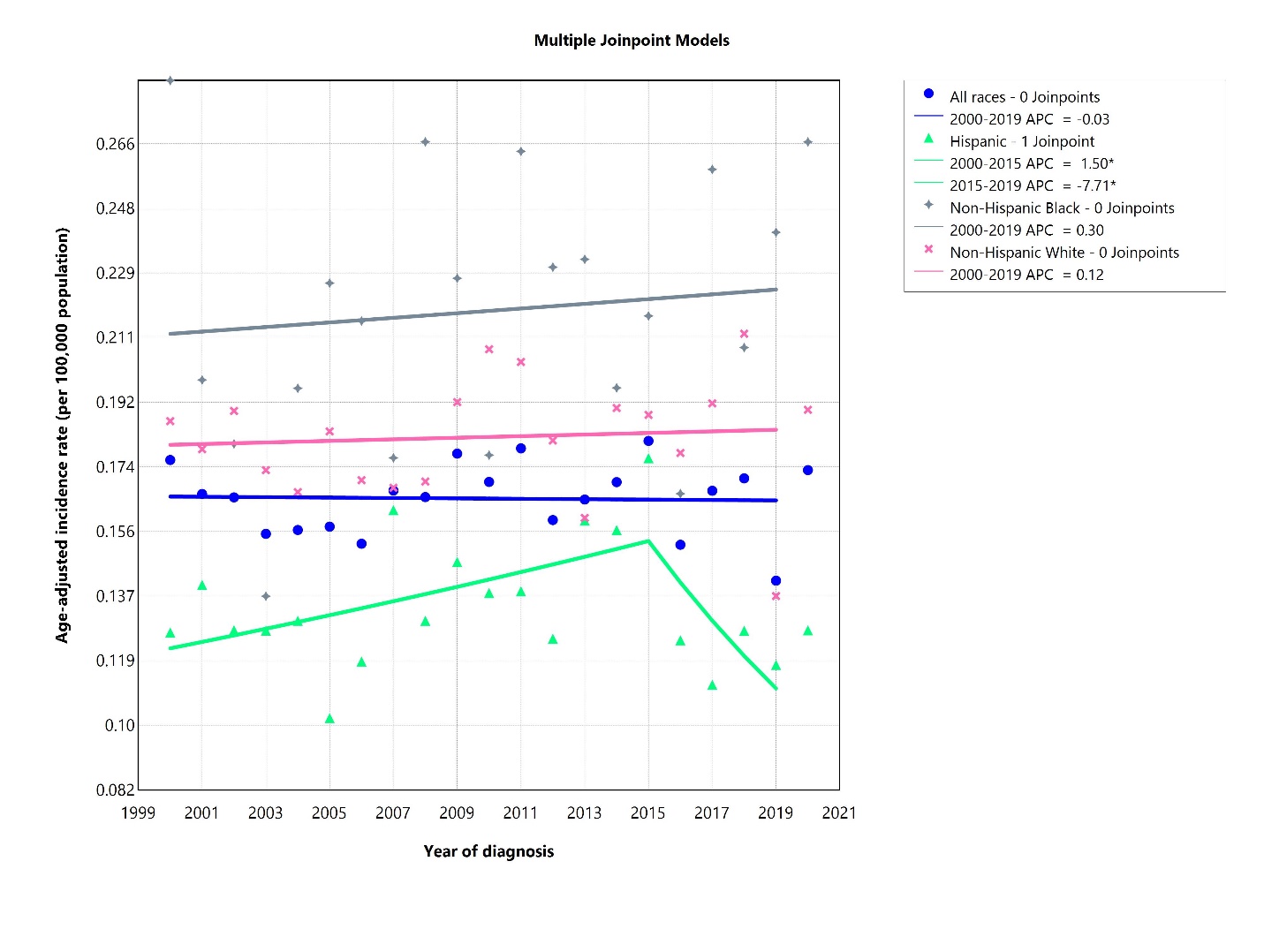


**Figure S10.** Age-adjusted incidence rate of nephroblastoma over 2000-2019 and in 2020 in the United States, by race. APC: annual percent change. * Represent p-value less than 0.05.


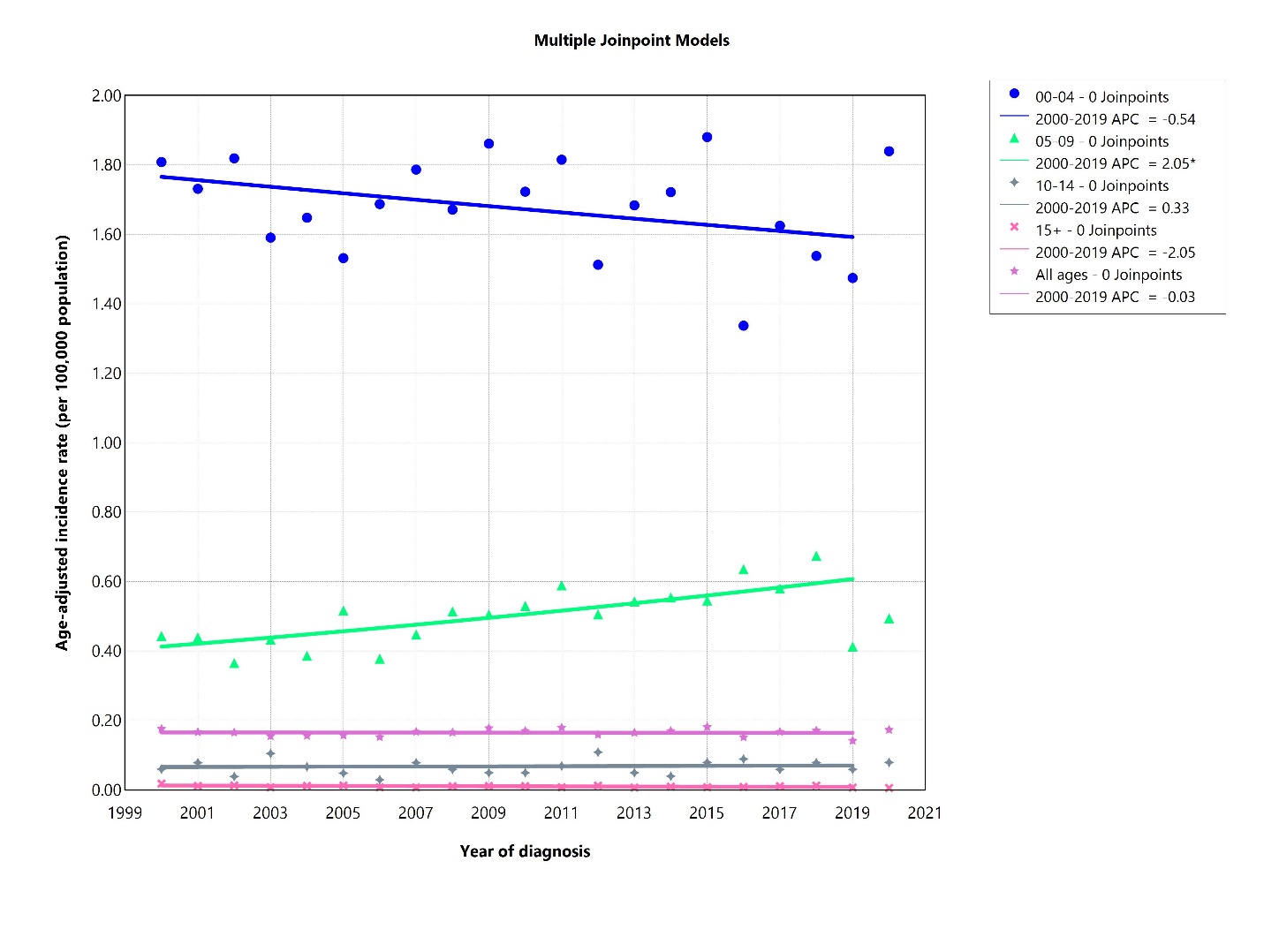
**Figure S11.** Age-adjusted incidence rate of nephroblastoma over 2000-2019 and in 2020 in the United States, by age. APC: annual percent change. * Represent p-value less than 0.05.


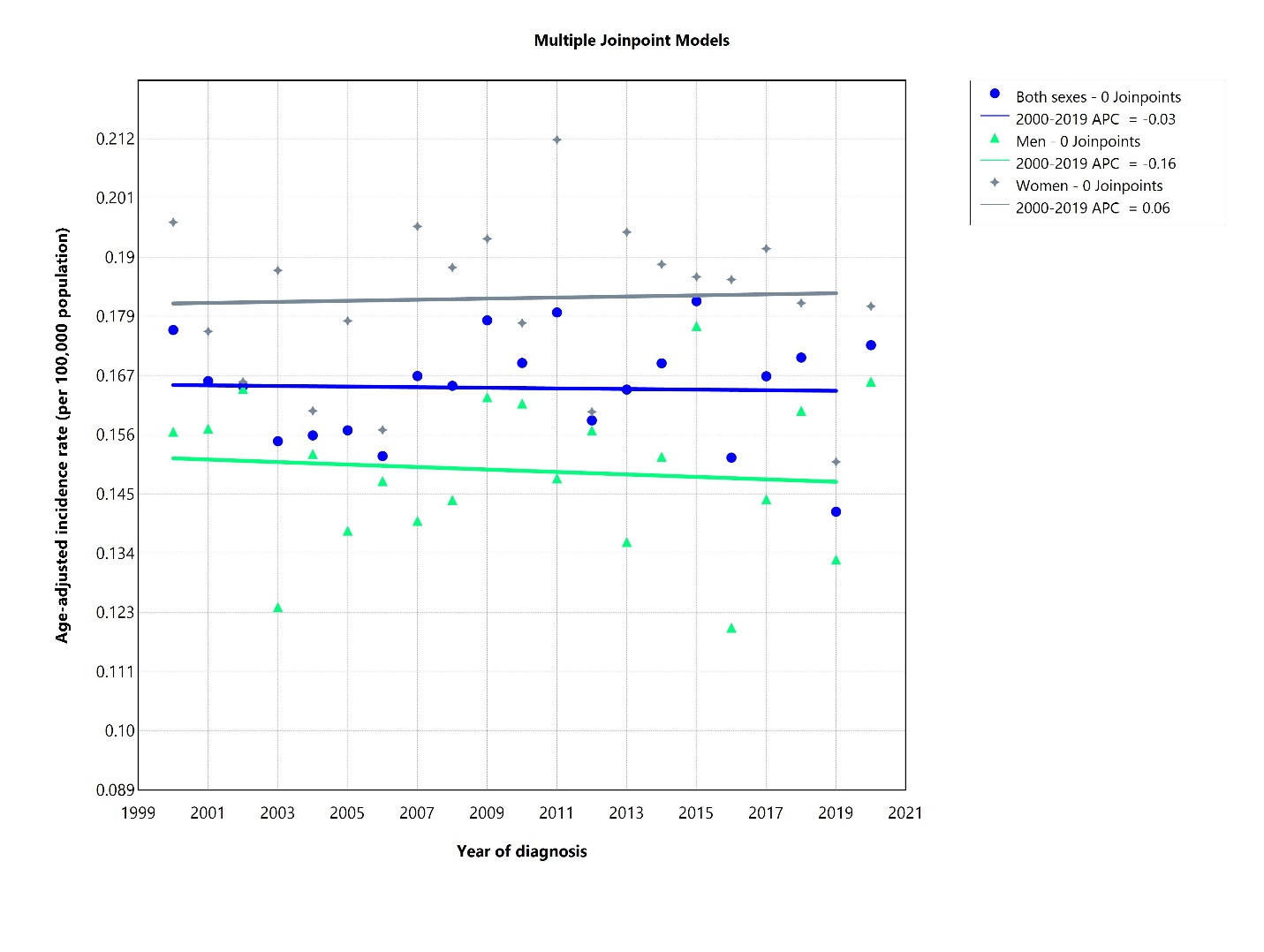
**Figure S12.** Age-adjusted incidence rate of nephroblastoma over 2000-2019 and in 2020 in the United States, by sex. APC: annual percent change.


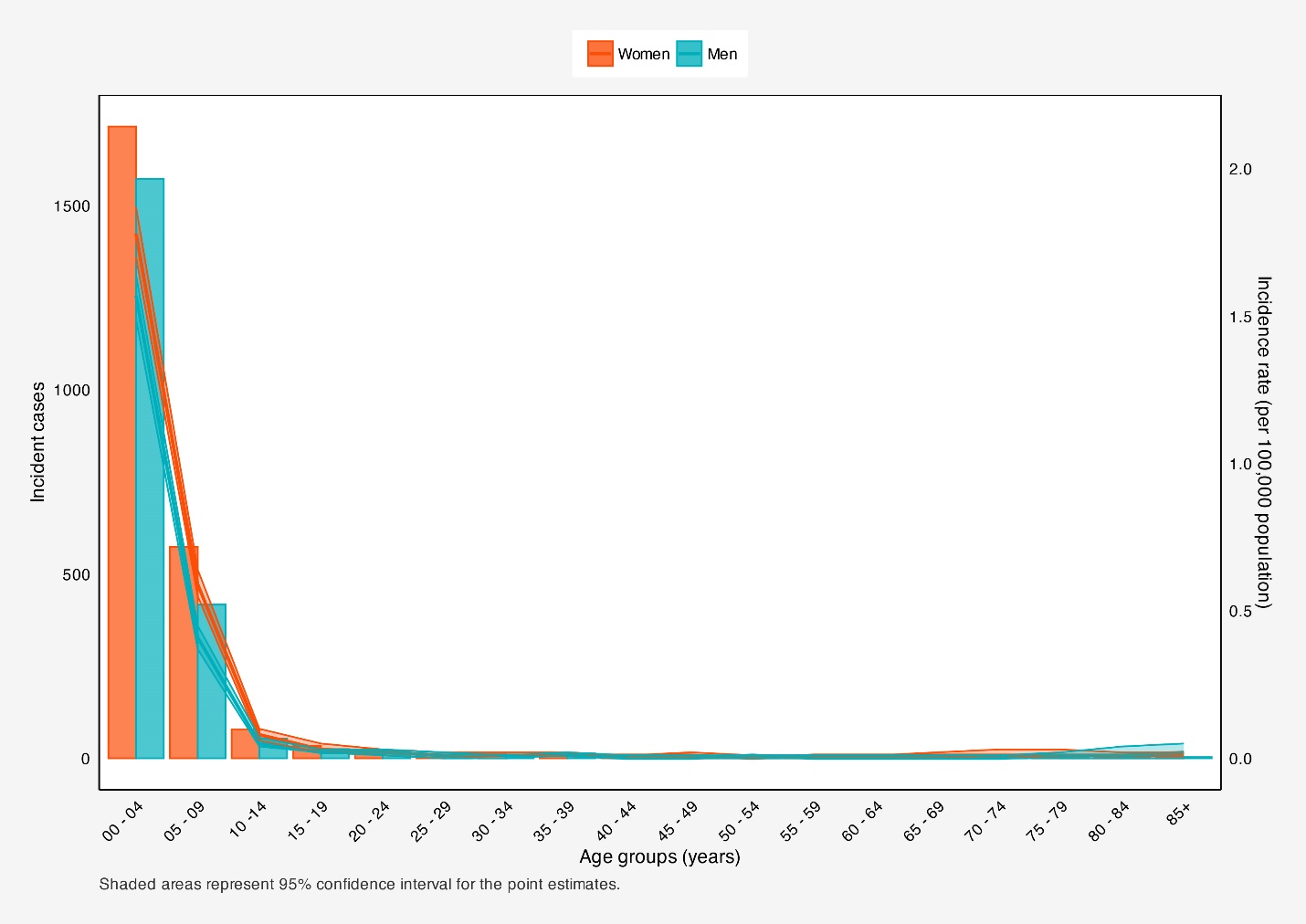


**Figure S13.** Incident cases and incidence rate of nephroblastoma in the United States among males and females in each age group. Shaded areas are the confidence interval range for the point estimates.


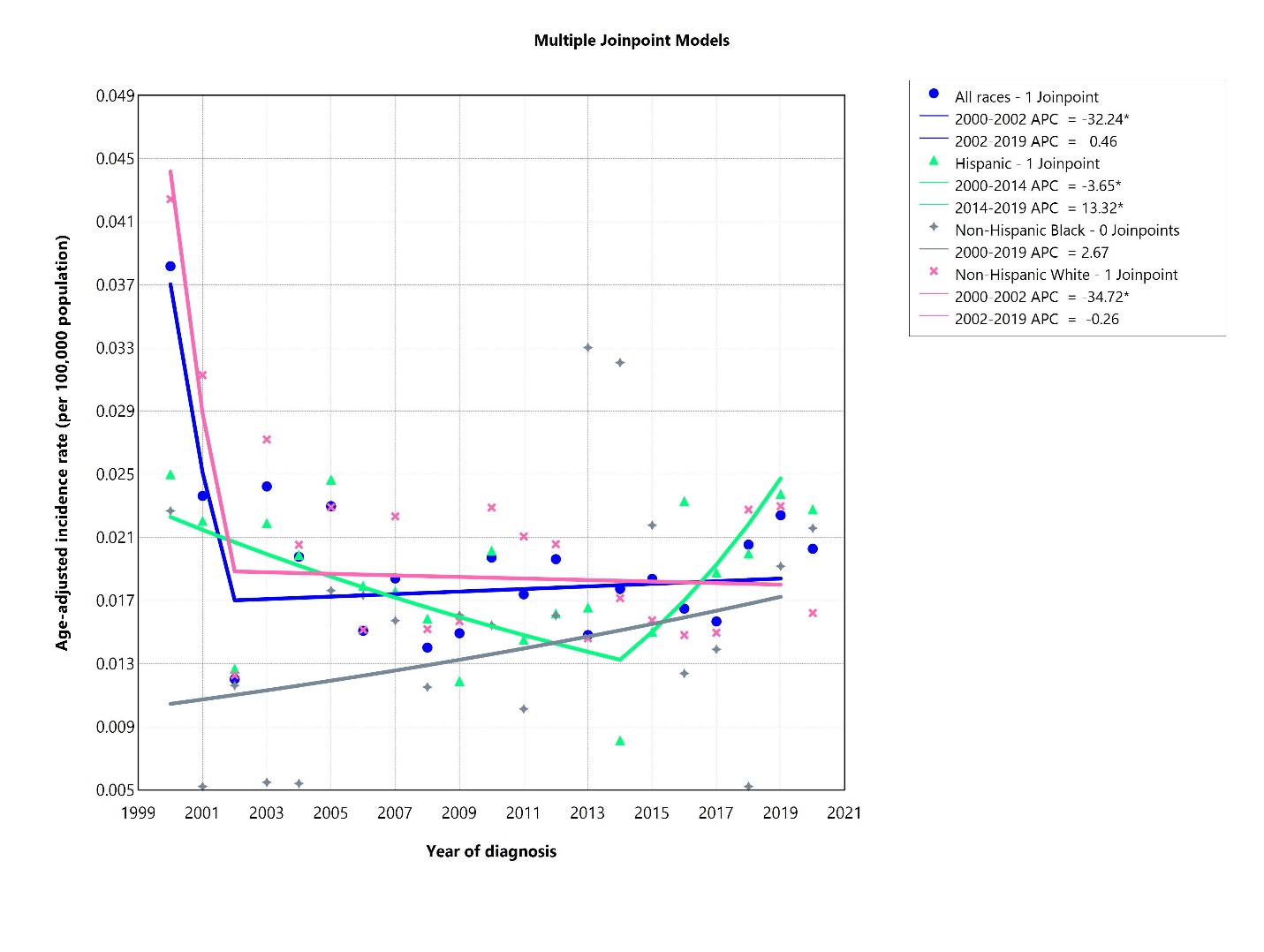
**Figure S14.** Age-adjusted incidence rate of sarcoma over 2000-2019 and in 2020 in the United States, by race. APC: annual percent change. * Represent p-value less than 0.05.


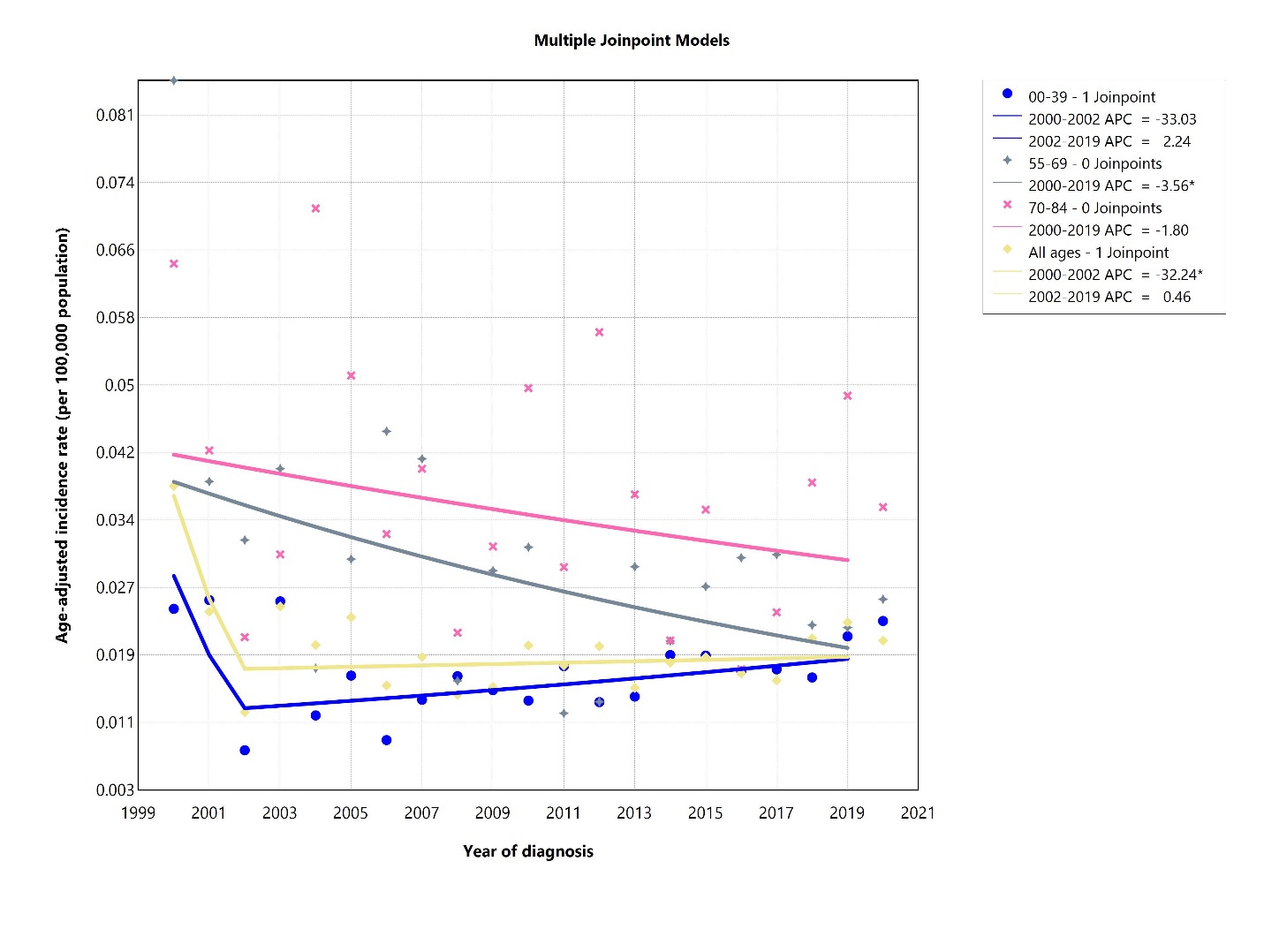
**Figure S15.** Age-adjusted incidence rate of sarcoma over 2000-2019 and in 2020 in the United States, by age. APC: annual percent change. * Represent p-value less than 0.05.


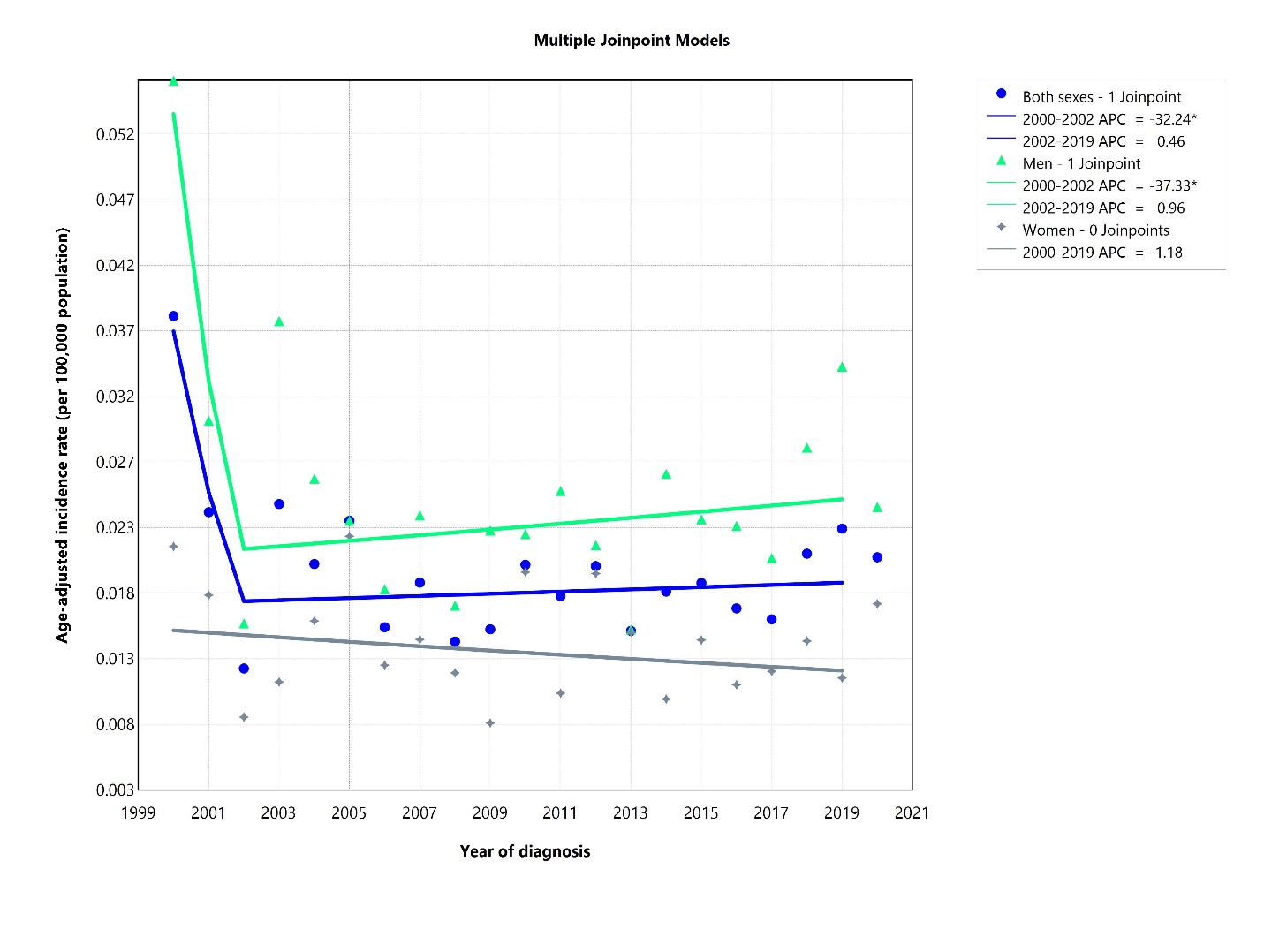
**Figure S16.** Age-adjusted incidence rate of sarcoma over 2000-2019 and in 2020 in the United States, by sex. APC: annual percent change. * Represent p-value less than 0.05.


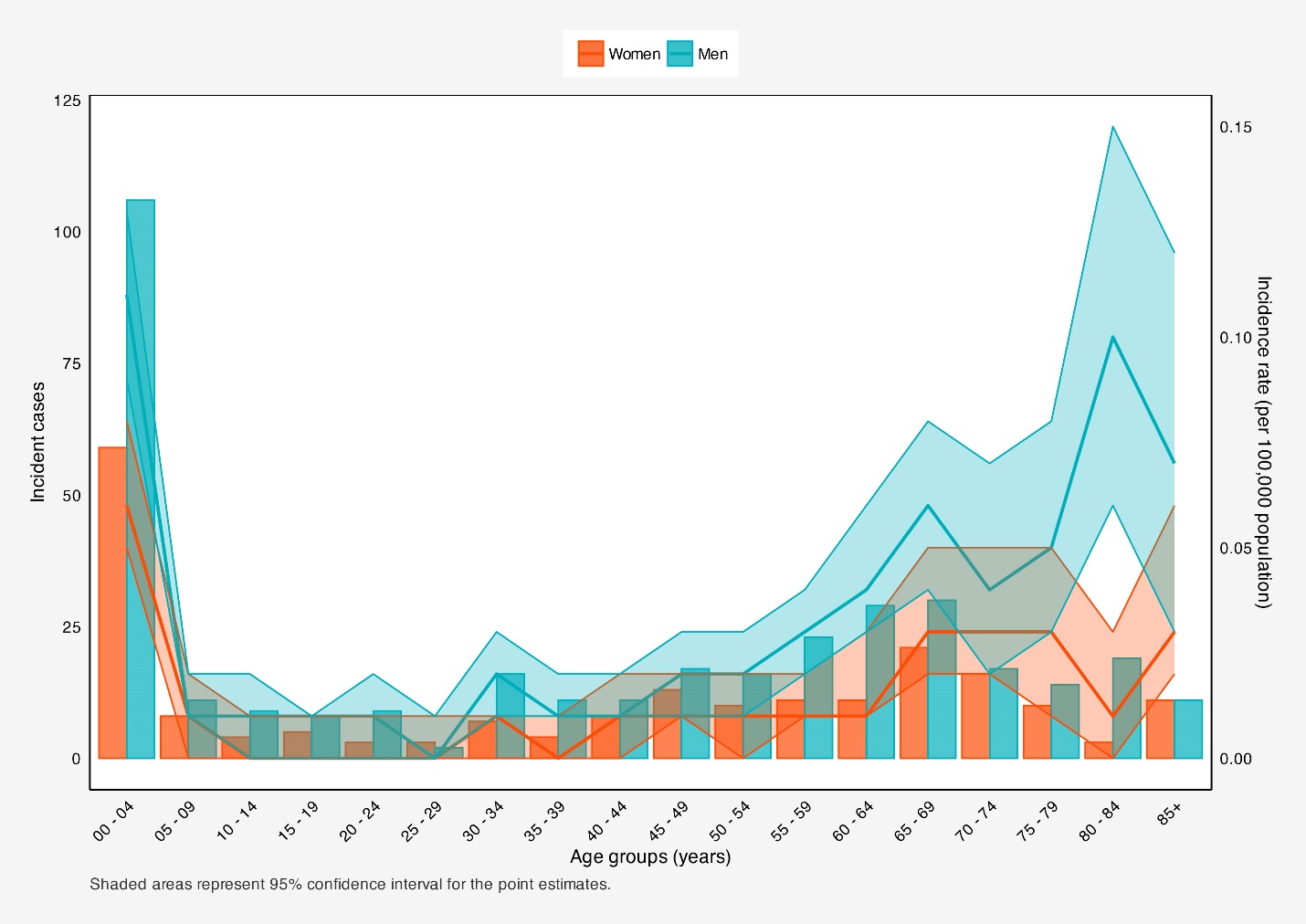


**Figure S17.** Incident cases and incidence rate of sarcoma in the United States among males and females in each age group. Shaded areas are the confidence interval range for the point estimates


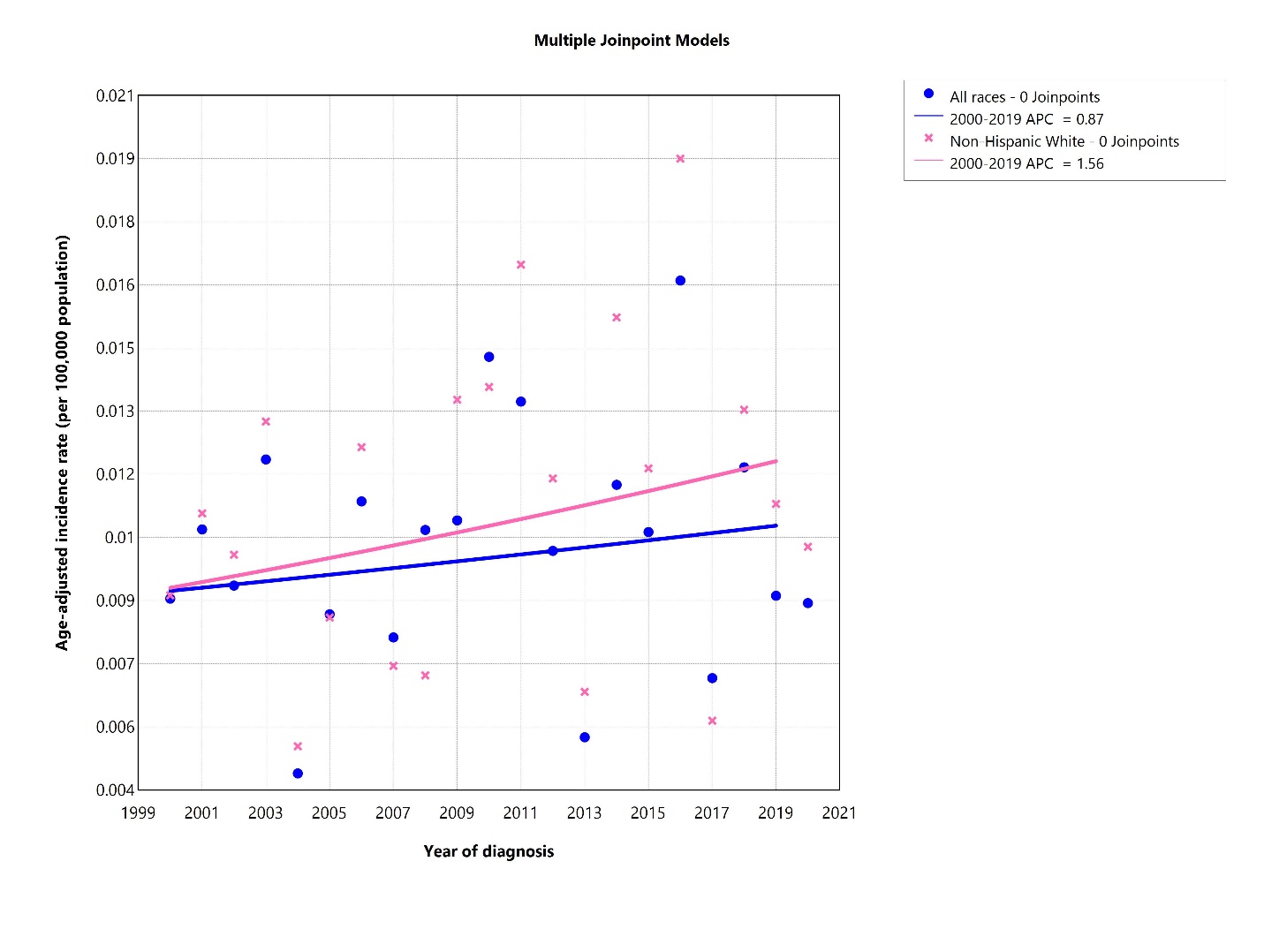
**Figure S18.** Age-adjusted incidence rate of neuroendocrine tumor over 2000-2019 and in 2020 in the United States, by race. APC: annual percent change.

**
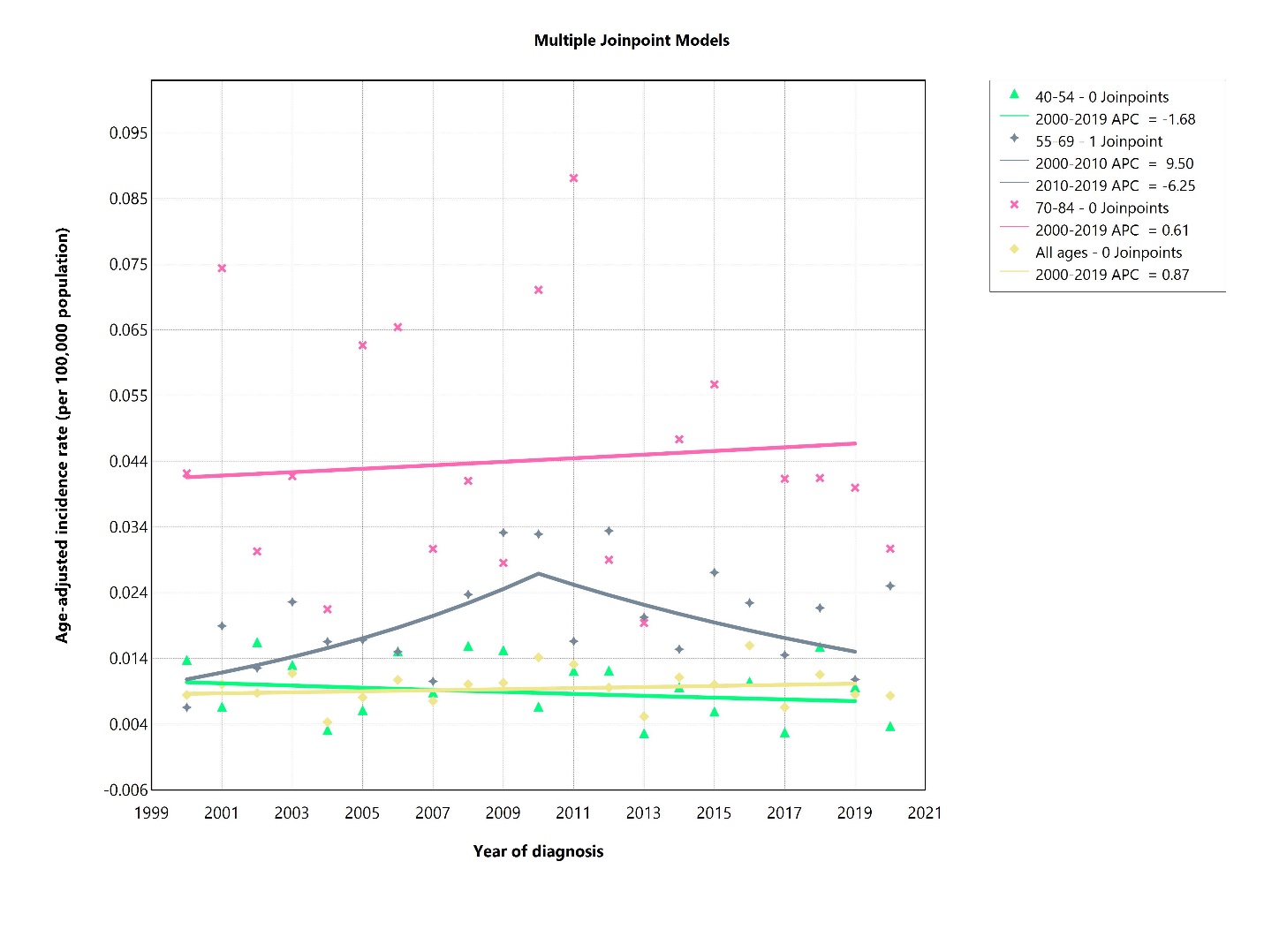
Figure S19.** Age-adjusted incidence rate of neuroendocrine tumor over 2000-2019 and in 2020 in the United States, by age. APC: annual percent change.

**
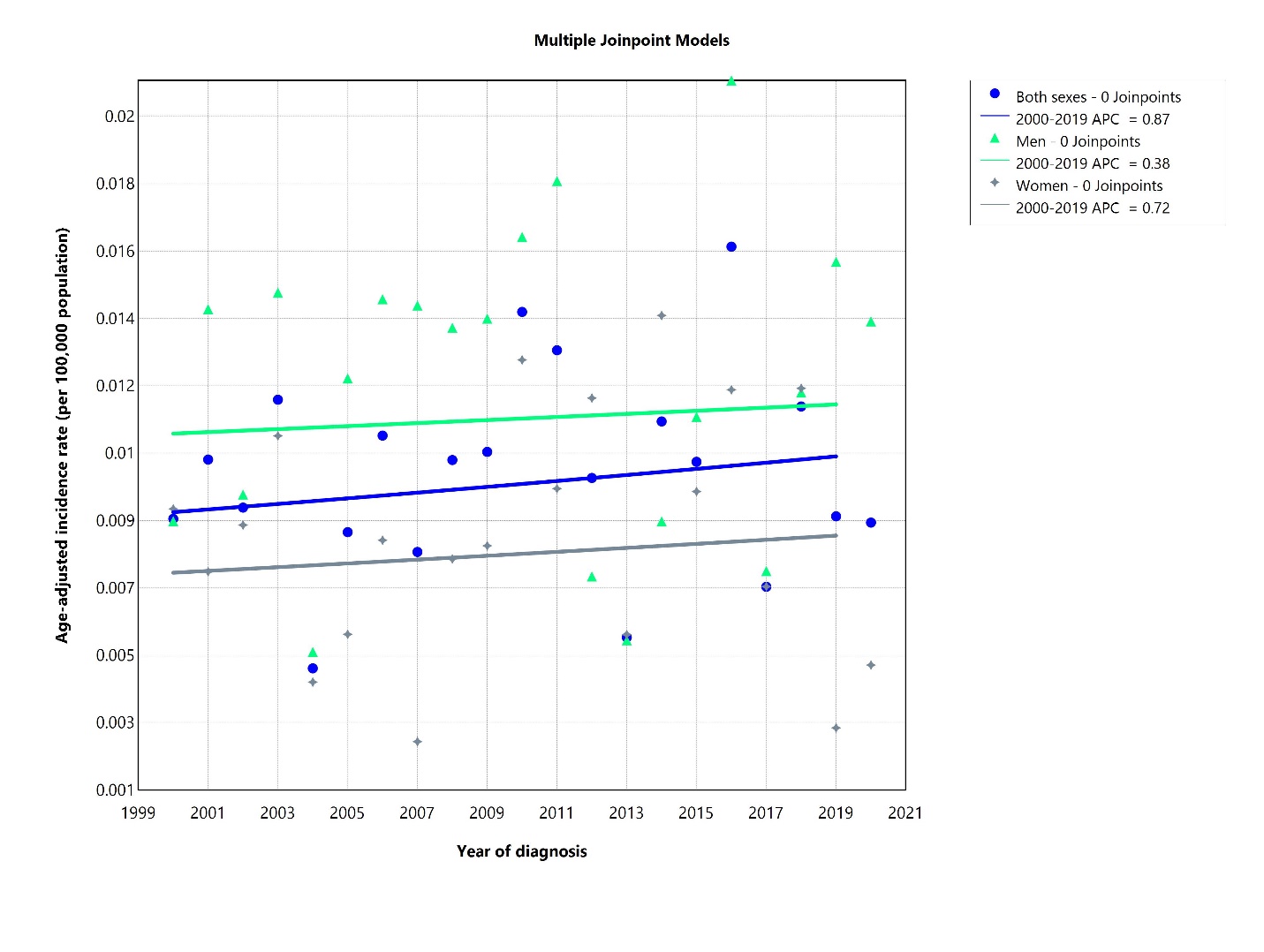
Figure S20.** Age-adjusted incidence rate of neuroendocrine tumor over 2000-2019 and in 2020 in the United States, by sex. APC: annual percent change.


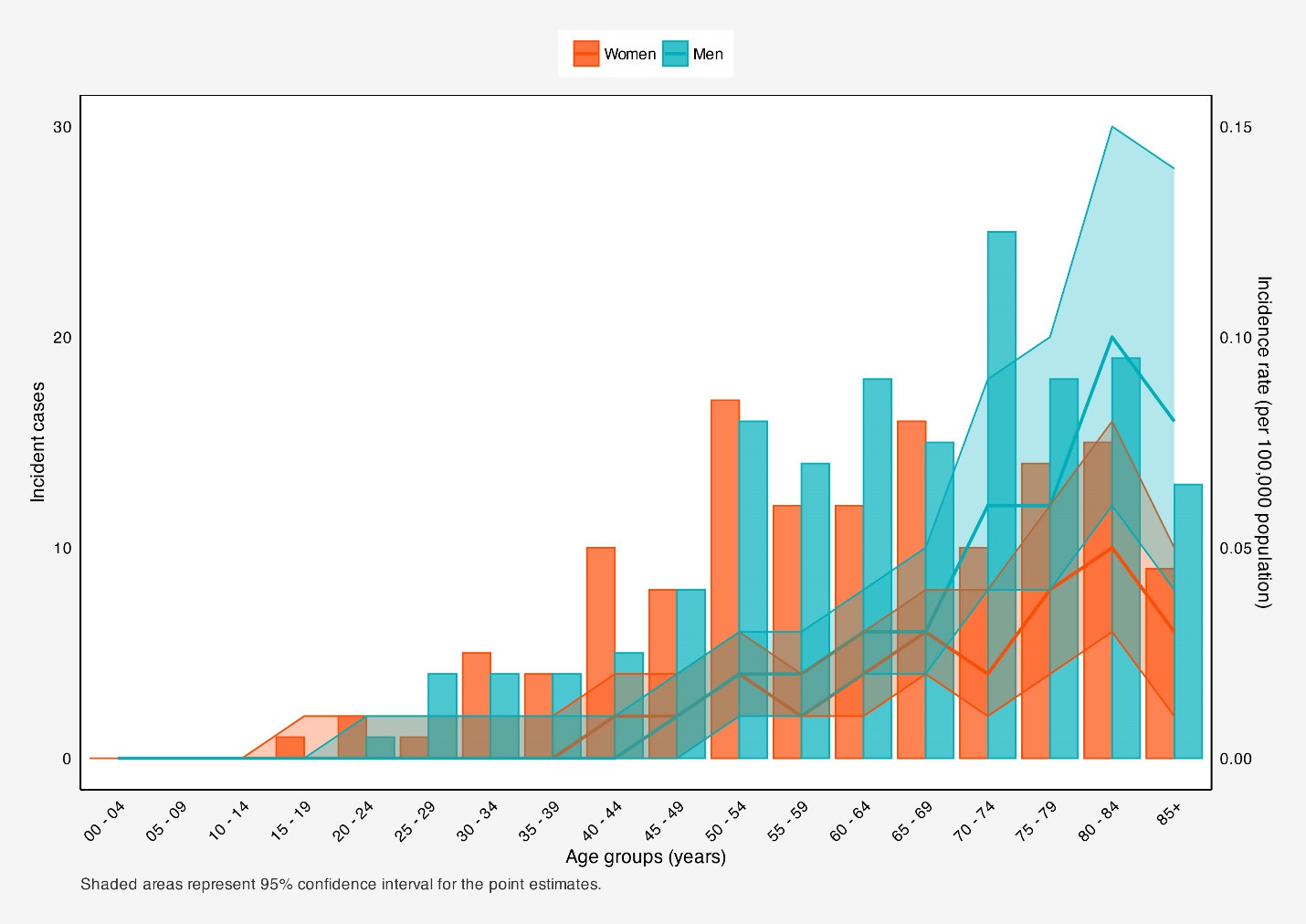


**Figure S21.** Incident cases and incidence rate of neuroendocrine tumor in the United States among males and females in each age group. Shaded areas are the confidence interval range for the point estimates


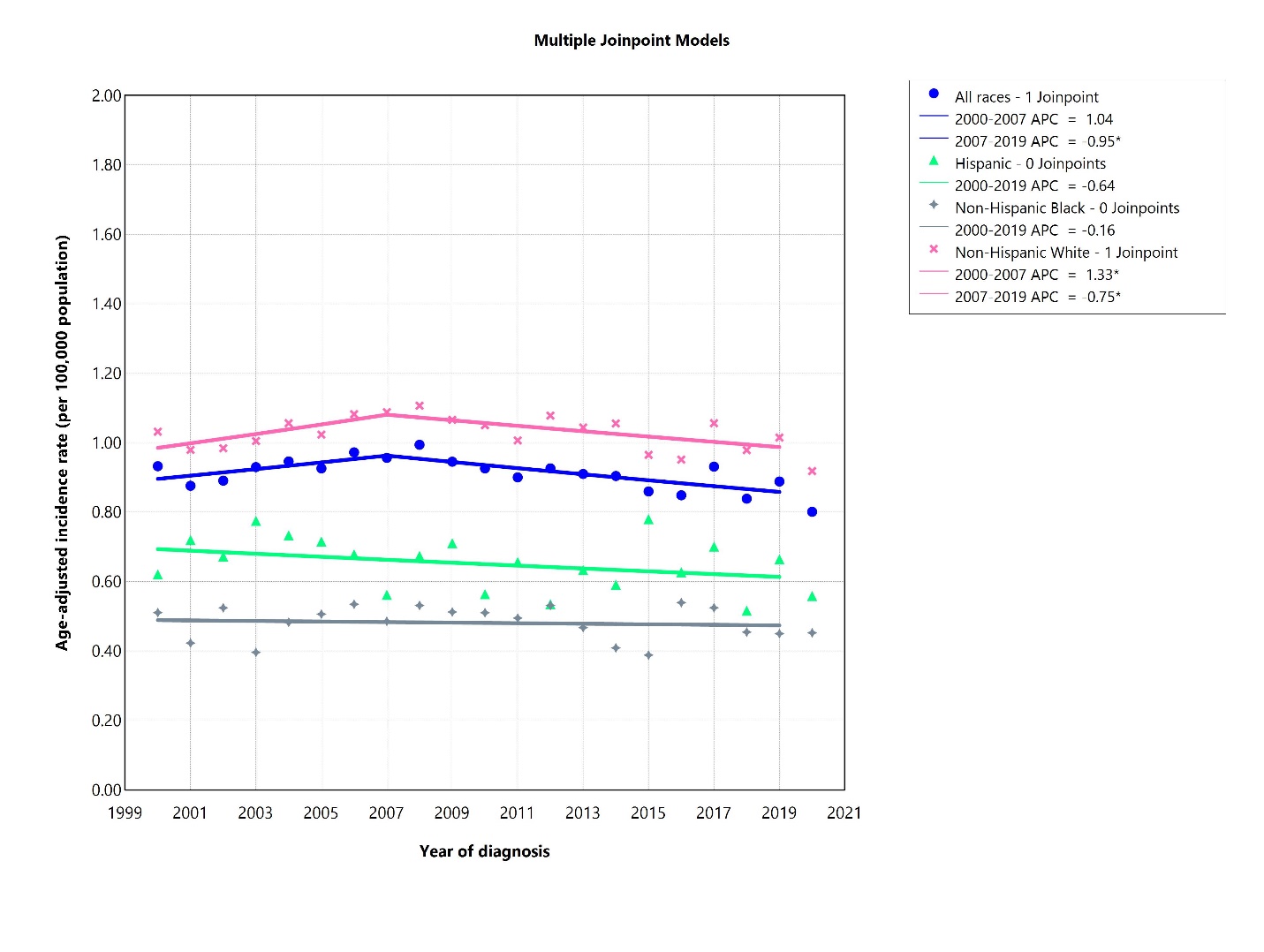


**Figure S22.** Age-adjusted incidence rate of renal pelvis cancer over 2000-2019 and in 2020 in the United States, by race. APC: annual percent change. * Represent p-value less than 0.05.


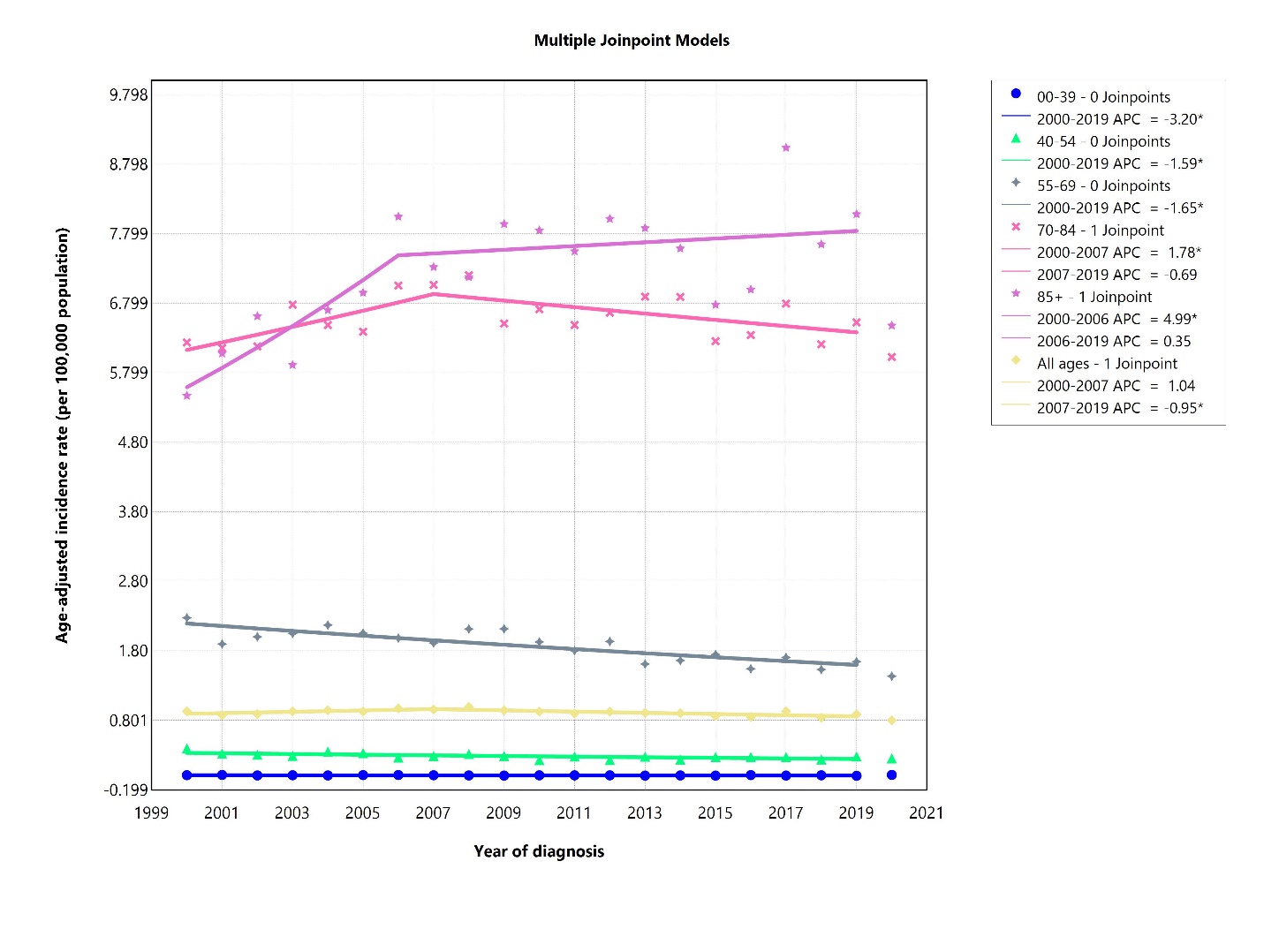
**Figure S23.** Age-adjusted incidence rate of renal pelvis cancer over 2000-2019 and in 2020 in the United States, by age. APC: annual percent change. * Represent p-value less than 0.05.


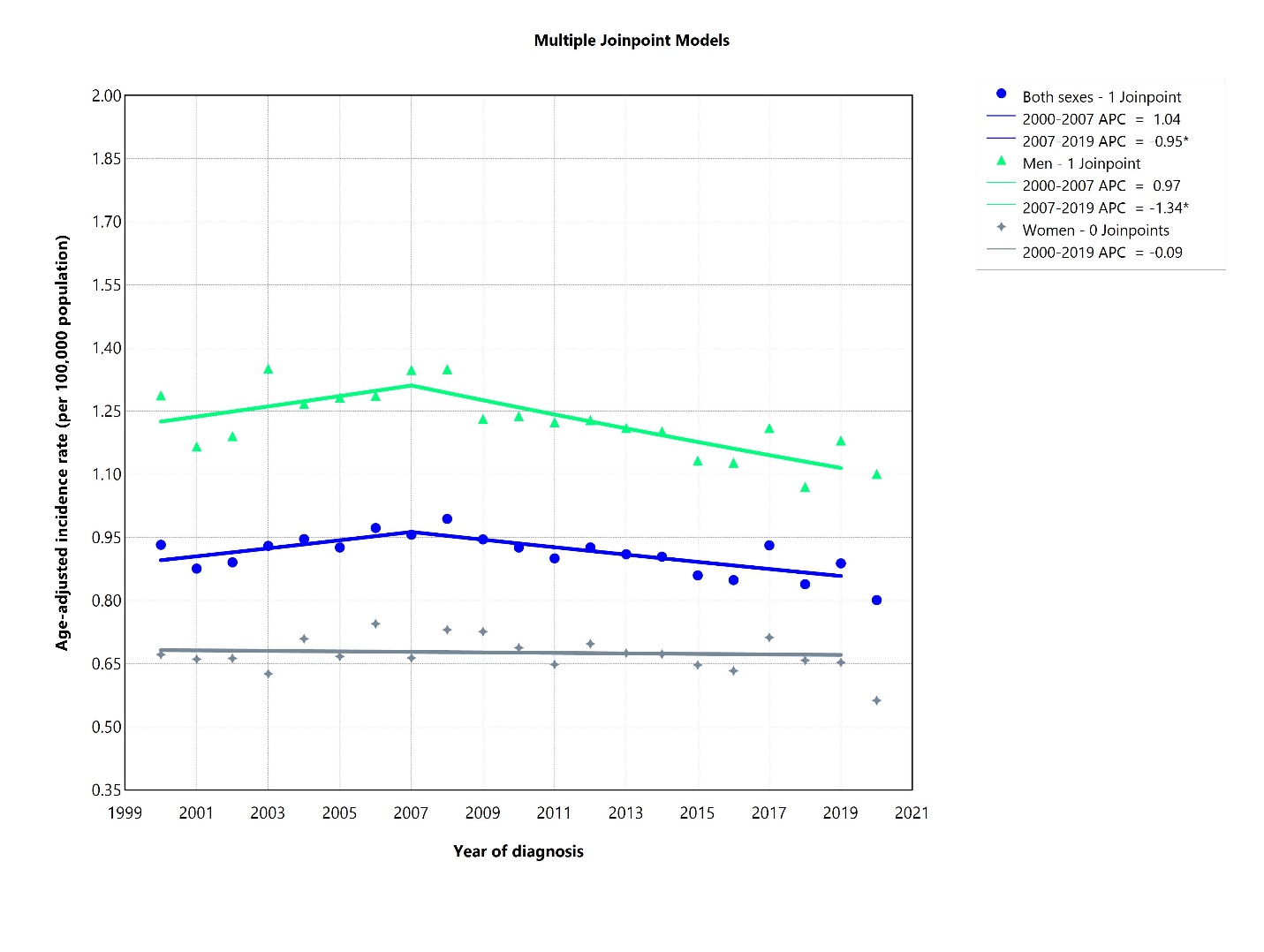
**Figure S24.** Age-adjusted incidence rate of renal pelvis cancer over 2000-2019 and in 2020 in the United States, by sex. APC: annual percent change. * Represent p-value less than 0.05.


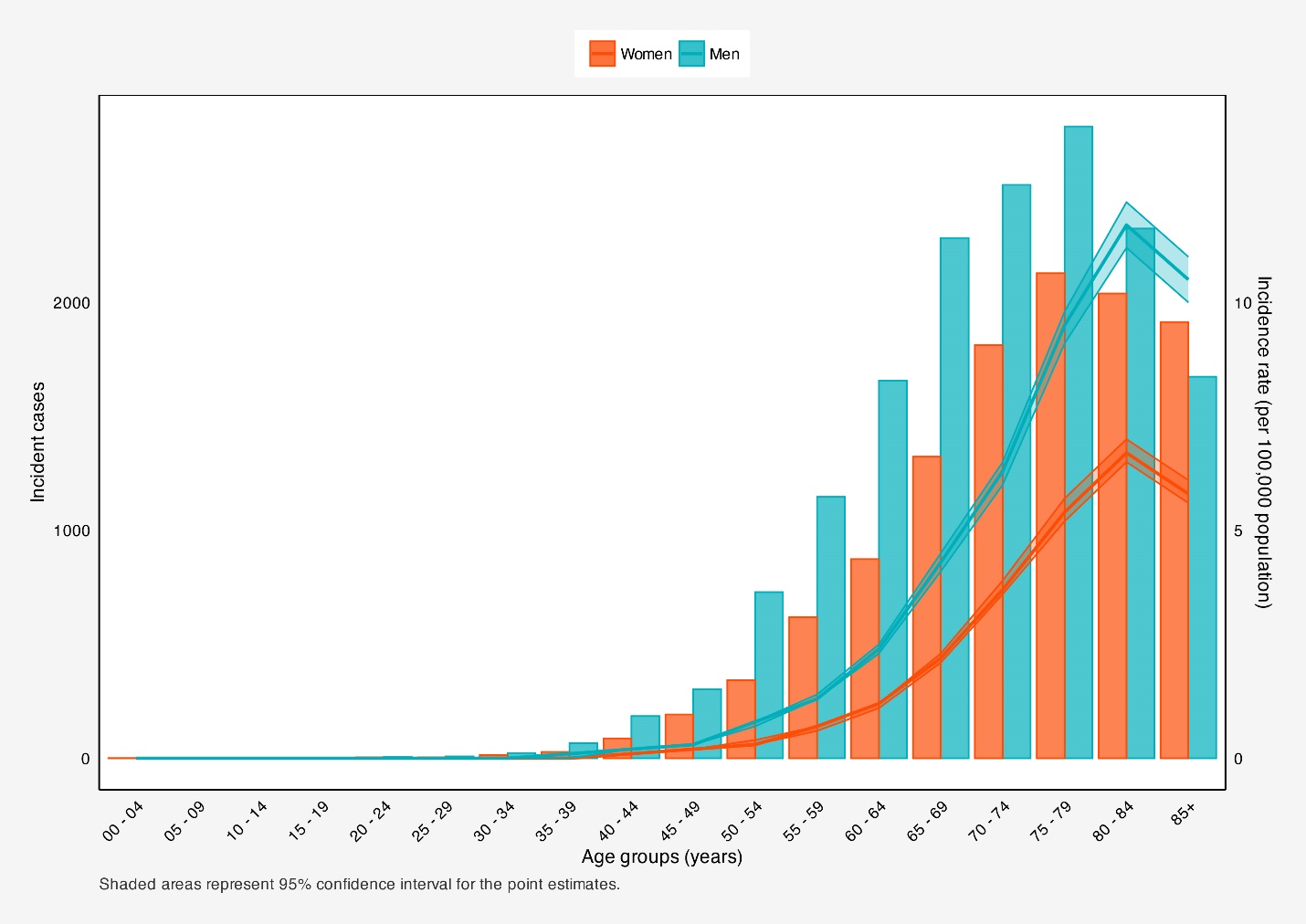


**Figure S25.** Incident cases and incidence rate of renal pelvis cancer in the United States among males and females in each age group. Shaded areas are the confidence interval range for the point estimates.
